# *M*/*P* Helicity Switching and Chiral Amplification in Double-Helical Monometallofoldamers

**DOI:** 10.1021/jacs.4c06560

**Published:** 2024-07-19

**Authors:** Kotaro Matsumura, Keigo Kinjo, Kotaro Tateno, Kosuke Ono, Yoshitaka Tsuchido, Hidetoshi Kawai

**Affiliations:** † Department of Chemistry, Faculty of Science, Tokyo University of Science, 1-3 Kagurazaka, Shinjuku-ku, Tokyo 162-8601, Japan; ‡ School of Science, Tokyo Institute of Technology, Tokyo 152-8551, Japan

## Abstract

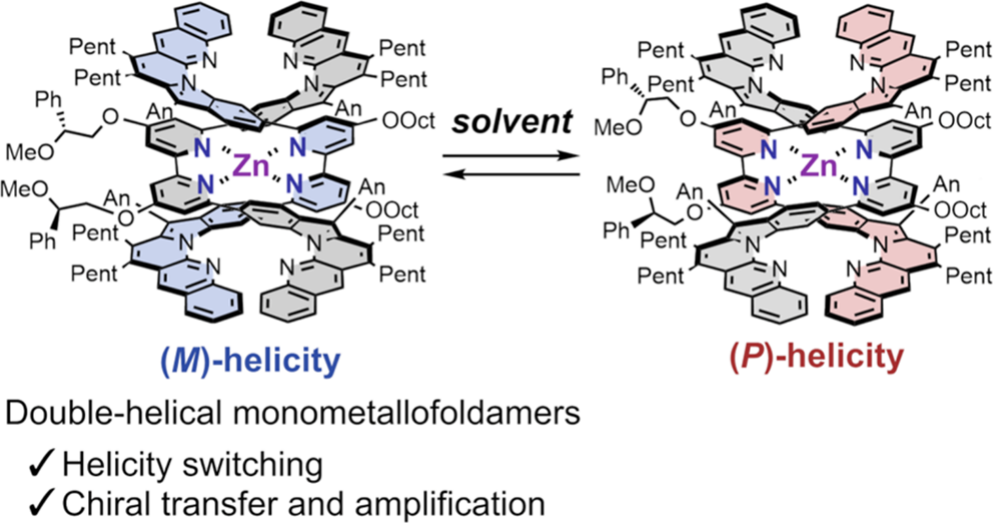

Short-stranded double-helical monometallofoldamers capable of *M*/*P*-switching were constructed by the complexation of two strands, each with two L-shaped units linked by a 2,2′-bipyridine, with a Zn(II) cation. The helix terminals of the “double-helical form” folded by π–π interactions can unfold in solution to equilibrate with the “open forms” that are favored at higher temperatures. Interestingly, the helical chirality of the monometallofoldamers with chiral side chains induced a single-handed helix sense and controlled *M*/*P*-switching depending on achiral solvent stimuli. For instance, the (*M*)-helicity was favored in nonpolarized solvents (toluene, hexane, Et_2_O), whereas the (*P*)-helicity was favored in Lewis basic solvents (acetone, DMSO). Circular dichroism (CD) and rotating-frame overhauser enhancement spectroscopy (ROESY) measurements revealed that the conformational change of the chiral side chains due to interaction of Lewis basic solvents with the double helices induced helicity bias. These novel double-helical monometallofoldamers possessed a stable helical structure and exhibited switchable chiroptical properties (*g*_abs_ ∼ 10^–3^–10^–2^). In addition, the chiral strand exhibited chiral transfer and amplification abilities through the formation of chiral heteroleptic double-helical monometallofoldamers when mixed with an achiral strand.

## Introduction

Helical foldamers^1−16^ have attracted attention as stimuli-responsive switchable molecules,^3−8^ tunable chiral materials,^9,10^ guest-selective receptors,^11−14^ and cooperative supramolecular systems^15,16^ due to their chiral and conformational switching properties. In particular, double-helical foldamers are kinetically stable and exhibit stronger chiral properties compared to single helices.^2^ Furthermore, double helices can exhibit distinctive properties,^17,18^ such as the transfer and transcription of chiral information from one chiral strand to the other achiral strand,^18^ and are expected to be applied to higher-order structural control related to replication, similar to nucleic acids.

**Scheme 1:**
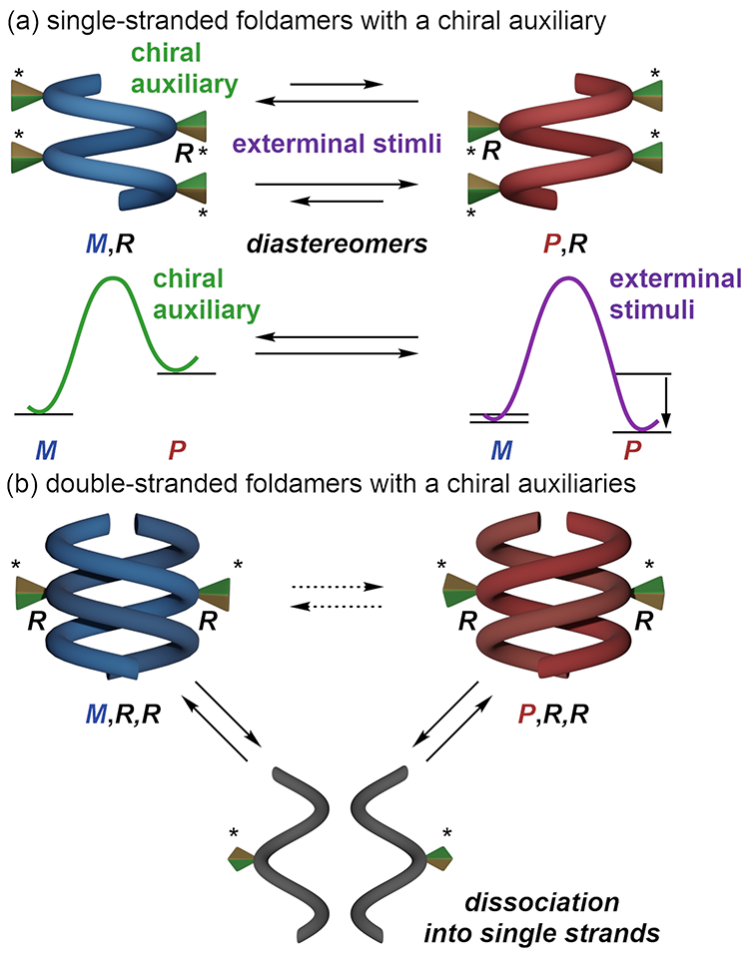
Helicity Inversion Induced by External Stimuli Diastereomeric pairs of dynamic helices induced by chiral auxiliaries and helicity inversion induced by external stimuli in (a) single-stranded foldamers and (b) double-stranded foldamers.

For switching and output chiral information, the development of chiral reversal systems (− → + or + → −) that exhibit larger output changes compared with just chiral induction (0 → ±) and the establishment of design guidelines for smaller molecules are very important. Along these lines, the reversal of chirality by achiral stimuli without exchanges of chiral auxiliaries is more attractive for switchable chiral materials.^19−34^ For example, some helical polymers and oligomers containing single-stranded foldamers (Scheme 1a),^20−25^ supramolecular gels,^26^ complexes,^27^ propeller-shaped molecules,^28,29^ and macrocycles^30,31^ exhibit chirality switching induced by achiral stimuli. Such switching has been reported in many helical polymers through cooperative effects of chiral auxiliaries toward external stimuli.^20−23^ However, switching of helicity in small molecules and double-helical foldamers is difficult. In the former, the helix sense stabilized by chiral auxiliaries must be destabilized without the assistance of cooperative effects, which are defined by the sum of small energy differences between side chains as in polymers. In the latter, due to the stability loss by dissociation into a single helix and the difficulty in balancing stability and dynamic properties, helicity inversion in double helices has been limited.^32−34^ Therefore, the compatibility of helicity inversion and amplification in double helices is still unexplored. For example, double-helical structures bridged by covalent bonds or coordination bonds to restrict dissociation into single helices have been developed to stabilize the double helix^35−38^ (Scheme 1b),^39−44^ while the switching properties are restricted in these more stable multibridged double helices.^45,46^

**Scheme 2:**
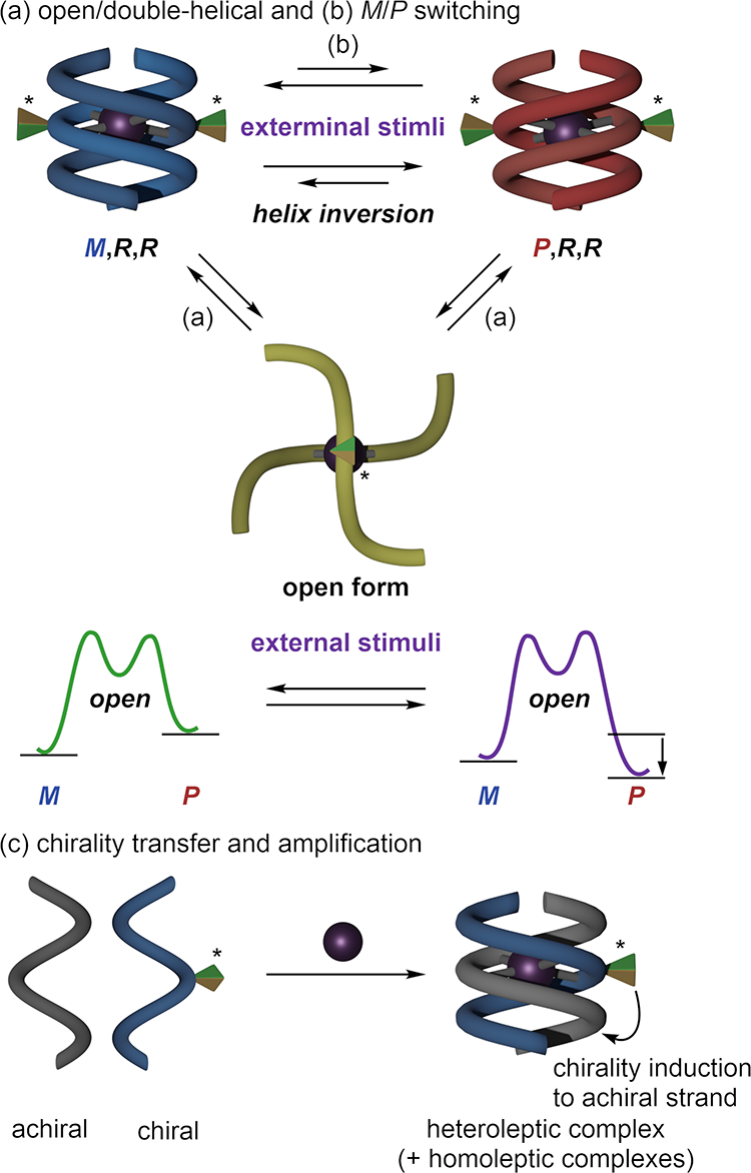
Dynamic and Chiral Properties of Double-Helical Monometallofoldamers (a) Open/double-helical and (b) *M*/*P* helicity switching of double-helical monometallofoldamers; (c) chirality transfer between a chiral strand and an achiral strand.

**Scheme 3:**
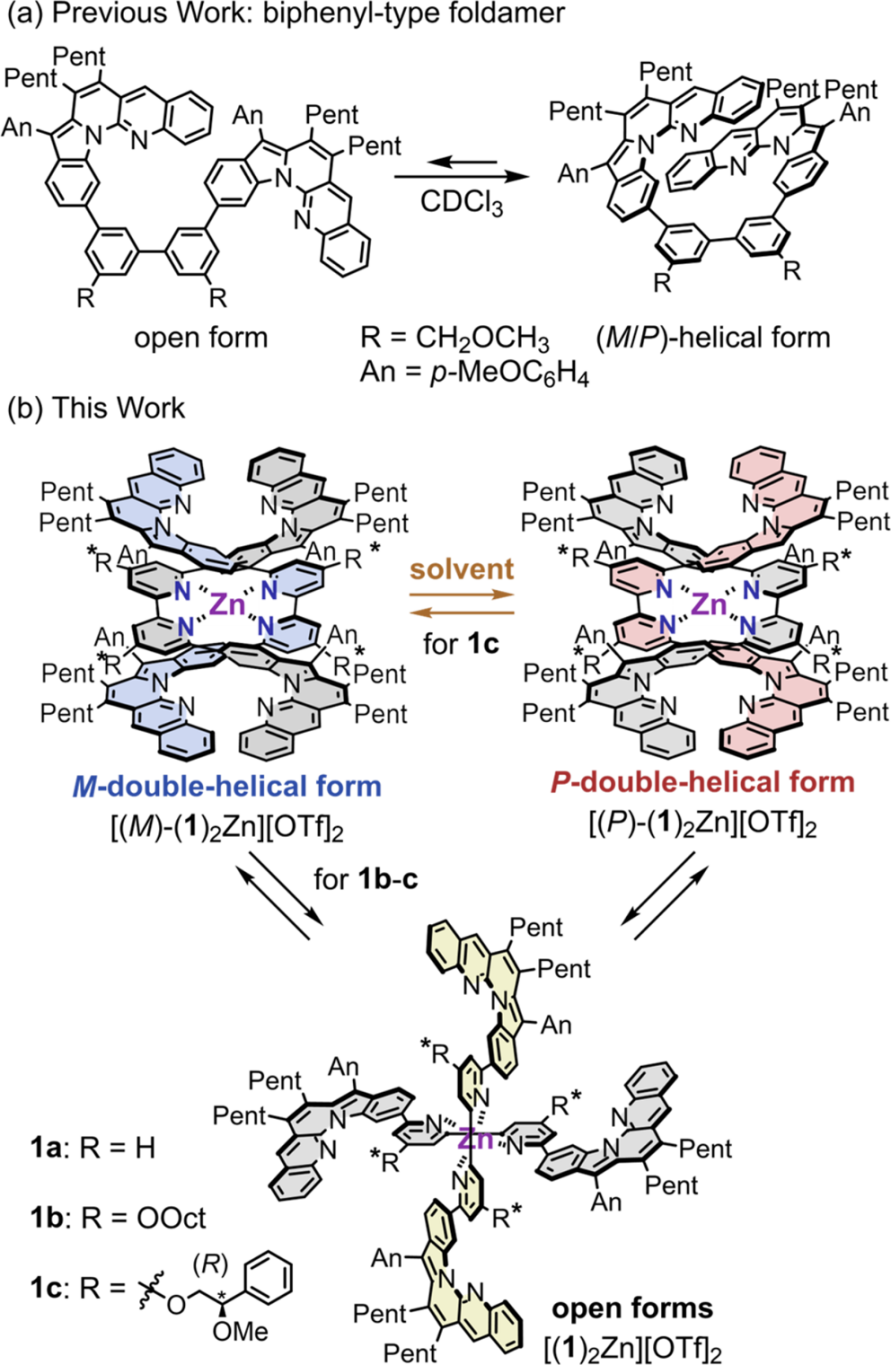
Helical Foldamers Based on L-shaped Dibenzopyrrolo[1,2-*a*][1,8]naphthyridine Double-helical/open type and *M*/*P*-double-helical type chirality switching in double-helical monometallofoldamers. (a) Biphenyl-type helical foldamer with two L-shaped units linked by a biphenyl spacer; (b) synthesis of metallofoldamers by complexation of bipyridine-type strands **1a**–**1c** with a zinc(II) cation and double-helical/open and *M*/*P*-switching of monometallofoldamers.

In this study, we designed the attractive mechanical motif, double-helical monometallofoldamers bridged with a single metal cation in the center of the helices, to balance both the stability and dynamic properties (Scheme 2). These monometallofoldamers would adopt a “double-helical form” bridged with a single cation at the central coordination site. The folded helix terminals could unfold in solution to equilibrate with the “open forms” (Scheme 2a) and refold to a double-helical form with opposite helicity, allowing *M*/*P*-switching control. Herein, we synthesized double-helical monometallofoldamers by bridging two strands **1a**–**1c** consisting of two L-shaped units and a 2,2′-bipyridine moiety with a single Zn(II) cation (Scheme 3). The monometallofoldamers [(**1c**)_2_Zn][OTf]_2_ with chiral side chains not only induced a single-handed helix sense in the double helix but also showed controllable *M*/*P*-switching in response to achiral solvent stimuli (Scheme 2b). In addition, the chiral strand exhibited chiral transfer and amplification abilities through the formation of chiral heteroleptic double-helical monometallofoldamers when mixed with achiral strands (Scheme 2c).

## Results and Discussion

### Synthesis of Foldamers **1a**–**c**

**Scheme 4:**
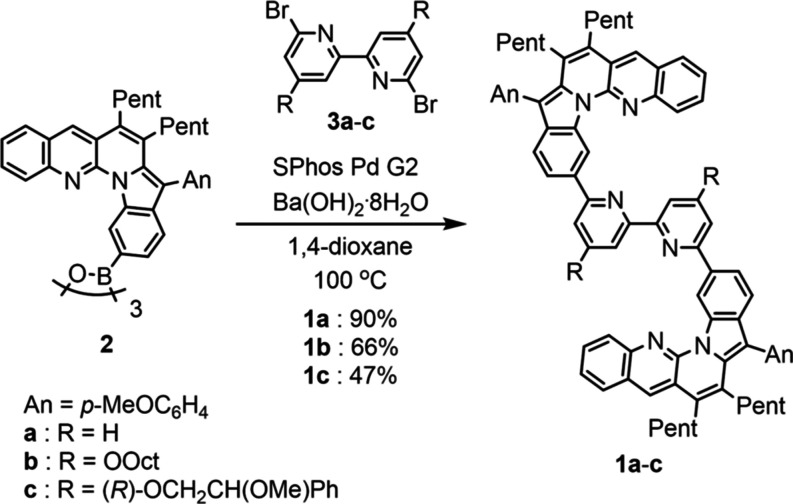
Synthesis of Bipyridine-Type Foldamers **1a**–**c** Reagents and conditions for **1a**: **2** (0.8 equiv), **3a** (1.0 equiv), SPhos Pd G2 (10 mol %), Ba(OH)_2_·8H_2_O (12 equiv), 1,4-dioxane, 100 °C, 1 d; for **1b**: **2** (0.8 equiv), **3b** (1.0 equiv), SPhos Pd G2 (5 mol %), Ba(OH)_2_·8H_2_O (6.0 equiv), 1,4-dioxane, 100 °C, 10 h; and for **1c**: **2** (0.8 equiv), **3c** (1.0 equiv), SPhos Pd G2 (10 mol %), Ba(OH)_2_·8H_2_O (6.0 equiv), 1,4-dioxane, 100 °C, 1 d.

We previously developed short-stranded helical foldamers containing L-shaped dibenzopyrrolo[1,2-*a*][1,8]naphthyridine^47,48^ units that folded into helical form in CDCl_3_ (Scheme 3).^49,50^ Based on this motif, three types of bipyridine-type strands **1a**–**c** with two L-shaped units were designed by incorporating a 2,2′-bipyridine spacer as a coordination site. Strands **1a**–**c** were prepared by Suzuki–Miyaura coupling of L-shaped unit **2**(49) with dibromobipyridines **3a**, **3b**, and **3c**, as shown in Scheme 4. Strand **1a** without substituents on the bipyridyl unit was prepared for X-ray structural analysis of the double-helical complex [(**1a**)_2_Zn][OTf]_2_ (see below). The complex [(**1a**)_2_Zn][OTf]_2_ was limited in solubility; therefore, **1b** with long octyloxy chains was prepared. Strand **1c** with (*R*)-2-methoxy-2-phenylethoxy groups as chiral auxiliaries was prepared to induce a single-handed helicity upon helix formation and study the inversion of the helicity by various external stimuli.

### Complexation and X-ray Structures of [(1)_2_Zn][OTf]_2_

**Figure 1:**
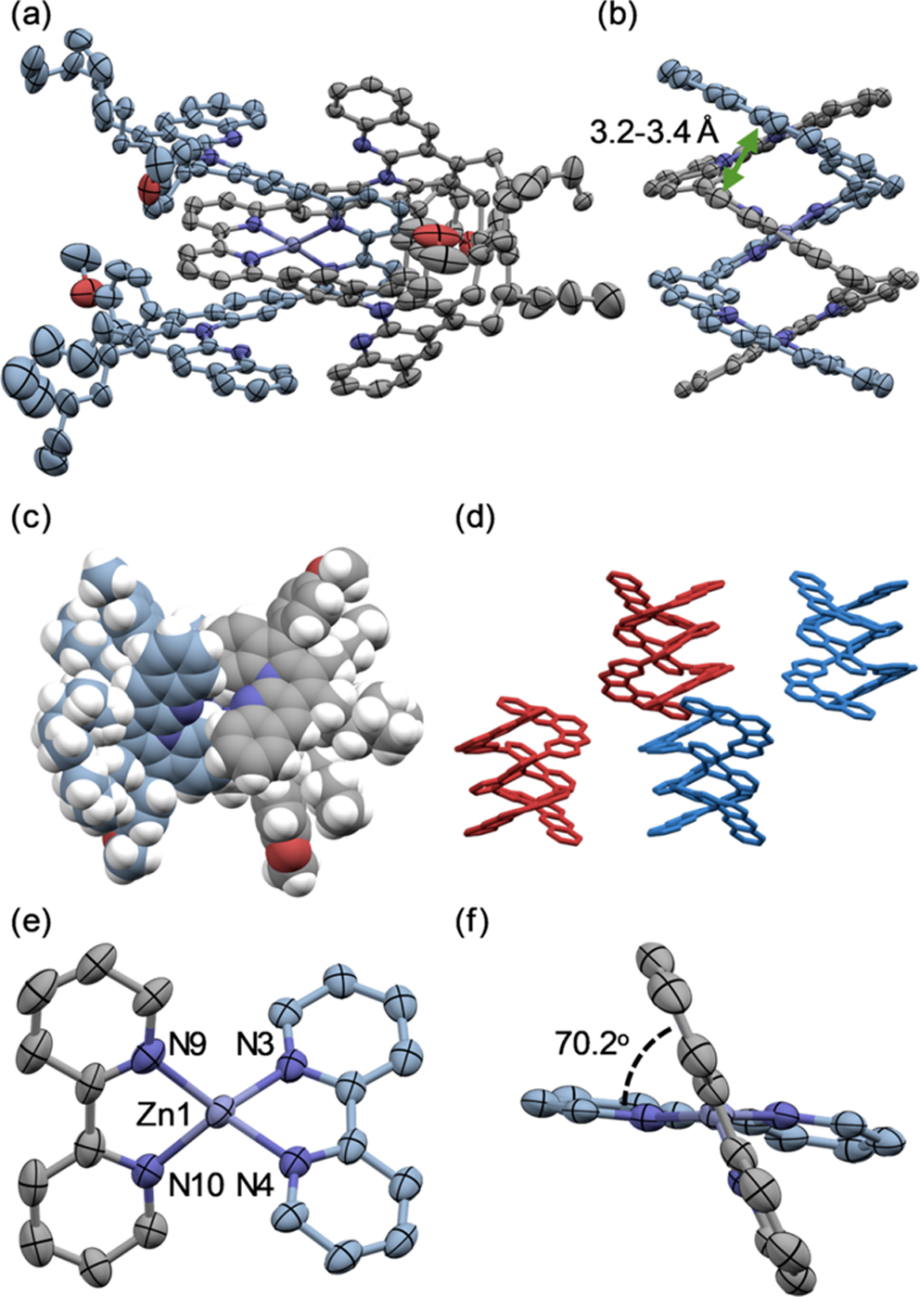
ORTEP drawings of X-ray structures with thermal ellipsoids at 50% probability; minor disordered parts and hydrogen atoms are omitted for clarity. Solvents and counteranions could not be analyzed sufficiently due to the disorder and were addressed through the implementation of the Solvent Mask routine. (a) Front view and (b) Side view (pentyl groups and anisyl groups are omitted for clarity) of [(**1a**)_2_Zn][OTf]_2_ (solvent mask); (c) top view (space-filling model); (d) crystal packing of complex [(**1a**)_2_Zn][OTf]_2_; [(*P*)-(**1a**)_2_Zn]^2+^ are shown in red and [(*M*)-(**1a**)_2_Zn]^2+^ are shown in blue (pentyl groups and anisyl groups are omitted for clarity); (e, f) coordination geometry of [(**1a**)_2_Zn]^2+^; selected atom distances and angles: N3–Zn1 2.023(3) Å, N4–Zn1 2.039(3) Å, N9–Zn1 2.030(3) Å, N10–Zn1 2.024(3) Å, N3–Zn1–N4 82.4(1)°, N4–Zn1–N9 117.3(1)°, N9–Zn1–N10 83.9(1)°, N10–Zn1–N3 111.1(1)°.

**Scheme 5:**
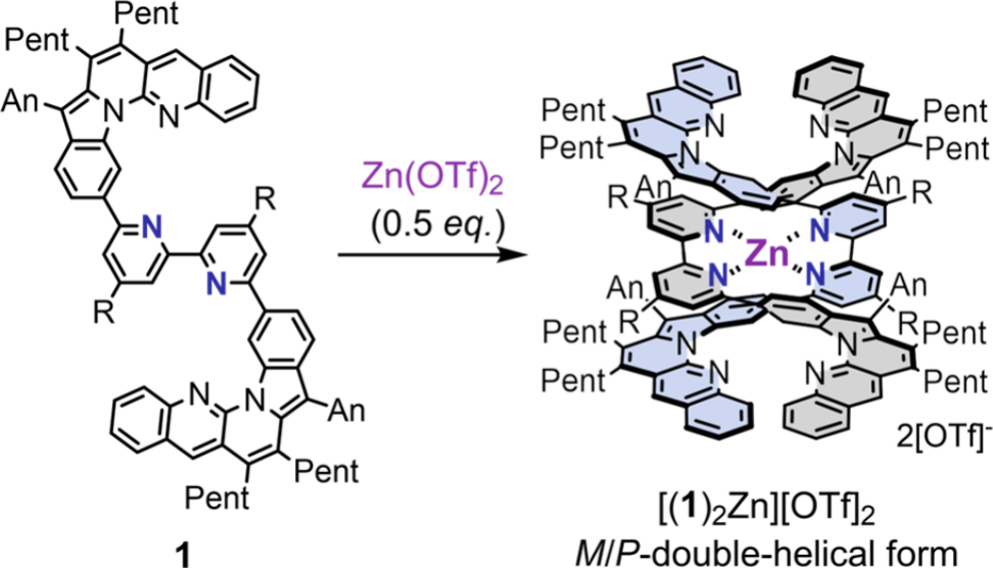
Complexation of Double-Helical Monometallofoldamer [(**1**)_2_Zn][OTf]_2_

Foldamers **1a**–**c** themselves did not form helical structures in solution or in the solid state due to the preference for the transoid conformation of the bipyridine spacer (Figure S5). Addition of zinc triflate [Zn(OTf)_2_] to foldamers **1a**–**c** promoted the formation of the double-helical complexes (Scheme 5). The zinc complex of **1a** was difficult to characterize in solution due to its poor solubility. However, single crystals of complex [(**1a**)_2_Zn][OTf]_2_ were obtained by the diffusion method from solutions, in which **1a** and a Zn(II) cation source were combined and sonicated for several hours. The structures of the complex [(**1a**)_2_Zn][OTf]_2_ was revealed by X-ray crystallography (Figure 1). [(**1a**)_2_Zn][OTf]_2_ adopted a double-helical mononuclear structure with two bipyridines coordinated to the zinc cation skewed tetrahedrally (Figure 1a,e,f). The two strands completely enclosed the Zn(II) cation (Figure 1c). The bipyridine spacer was stacked between the two L-shaped units with a stacking distance of 3.2–3.4 Å (Figure 1b). Furthermore, the crystals contained (*M*)- and (*P*)-double helices, and those *M*/*P*-forms were stacked alternately (Figure 1d).

### ^1^H NMR Studies of Complex [(1b)_2_Zn][OTf]_2_

**Figure 2:**
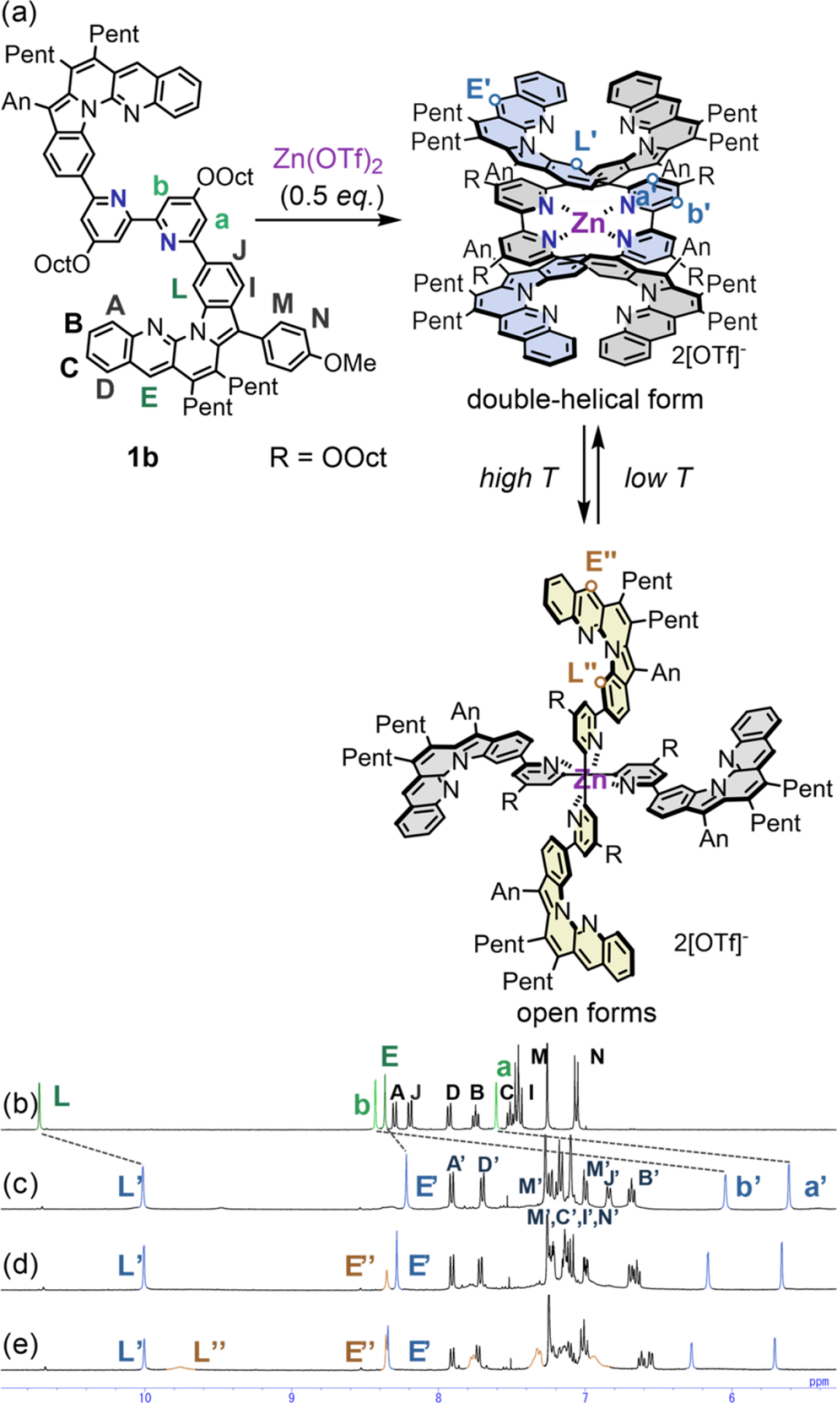
(a) Complexation of **1b** with Zn(II), dynamic behavior of monometallofoldamer [(**1b**)_2_Zn][OTf]_2_ and ^1^H NMR analysis (5–11ppm) of (b) **1b** in CDCl_3_ at 298 K, (c) [(**1b**)_2_Zn][OTf]_2_ in CDCl_3_ at 273 K, (d) 298 K, and (e) 323 K ([**1b**] = 2.0 mM, [(**1b**)_2_Zn][OTf]_2_ = 1.0 mM).

To examine the conformation of double-helical monometallofoldamers, ^1^H NMR spectra of [(**1b**)_2_Zn][OTf]_2_, which had better solubility than [(**1a**)_2_Zn][OTf]_2_, were measured upon the addition of Zn(OTf)_2_ (0.5 equiv) to **1b** in CDCl_3_. The complex [(**1b**)_2_Zn][OTf]_2_ adopted both double-helical and open form conformations, which were in equilibrium (Figure 2a). The bipyridine protons H_a_’ and H_b_’ of the complex shifted significantly upfield (5.6–6.2ppm) compared to **1b**, despite coordination to a zinc cation, suggesting the stacking of the L-shaped unit and two bipyridine units (shown in blue in Figure 2b). We assigned the species with these peaks to the double-helical form of [(**1b**)_2_Zn][OTf]_2_ comparable to that observed in the X-ray structure of [(**1a**)_2_Zn][OTf]_2_. Interestingly, NMR studies revealed additional conformations of [(**1b**)_2_Zn][OTf]_2_. In CDCl_3_, the chemical species exhibited broad peaks (shown in orange in Figure 2c–e) that intensified with increasing temperature, suggesting the formation of another complex.

DOSY analyses revealed that the hydrodynamic radius of this complex was approximately equivalent to the double-helical form (*D* = 5.55 × 10^–10^ m^2^·s^–1^ obtained from peak E″, *D* = 5.61 × 10^–10^ m^2^·s^–1^ obtained from peak E′ of the double-helical form), suggesting this complex could have the same composition as the double-helical form of [(**1b**)_2_Zn][OTf]_2_ (Figure S15). EXSY analyses suggested a chemical exchange between the double-helical form of [(**1b**)_2_Zn][OTf]_2_ and this complex (Figure S14), suggesting that this complex is a conformational isomer of the double-helical form. As a candidate for this conformational isomer, the existence of an “open form” with four L-shaped units facing outward was suggested from the preliminary X-ray structure of [(**1a**)_2_Ag][PF_6_] (Figure S7). The remarkably broad peaks of the open forms suggested that multiple conformations were in equilibrium on the NMR time scale, making them indistinguishable at 258–323 K, at least in CDCl_3_ (Figure S18). Therefore, all conformers with one or more L-shaped units facing outward, except the double-helical form, were defined as open forms (Figure S16).

We expected that the two forms were in equilibrium and interconvertible by rotation of the L-shaped units. Therefore, we investigated the equilibrium behavior and thermodynamic parameters of [(**1b**)_2_Zn][OTf]_2_. Variable-temperature ^1^H NMR revealed that the double-helical form with dense π-stacking was enthalpically favored, while the open forms with a high mobility of the L-shaped units were entropically favored (Figure S18). The van’t Hoff plots revealed that Δ*H*_open→helix_ and Δ*S*_open→helix_ for the conversion of open forms to the double-helical form were both negative [Δ*S*_open→helix_ = −7.1 ± 1.0 cal·mol^–1^·K^–1^, Δ*H*_open→helix_ = −2.4 ± 0.3 kcal·mol^–1^ in CDCl_3_] (Figure S19).

The equilibrium between the double-helical form and the open forms in various solvents was investigated, revealing that the effect of the solvents was not simple (Table 1 and Figure S17). In CD_3_OD/CDCl_3_ or CD_3_CN/CDCl_3_, the enthalpy difference Δ*H* increased negatively, suggesting that the double-helical form was favored due to solvation of polar solvents to the double-helical form. The entropy difference Δ*S*_open→helix_ also increased due to the decreasing degrees of freedom of the solvated double-helical form and solvating solvents. Interestingly, in bulky THF-*d*_8_ and nonpolar toluene-*d*_8_, Δ*H*_open→helix_ and Δ*S*_open→helix_ decreased, suggesting that bulky solvents provided less solvation.

**Table 1:** Solvent-Dependent Thermodynamic Parameters in [(1b)_2_Zn][OTf]_2_^a^

solvent(s)	Δ*H*_open→helix_ (kcal·mol^–1^)	Δ*S*_open→helix_ (cal·mol^–1^·K^–1^)	Δ*G*_open→helix_^b^ (kcal·mol^–1^)
CD_3_OD/CDCl_3_ = 1:2	–8.2 ± 2.0	–25.0 ± 6.5	–0.7 ± 2.8
CD_3_CN/CDCl_3_ = 1:2	–5.2 ± 1.0	–15.3 ± 3.0	–0.7 ± 1.3
CDCl_3_	–2.4 ± 0.3	–7.1 ± 1.0	–0.3 ± 0.4
THF-*d*_8_	–1.9 ± 0.3	–5.5 ± 1.0	–0.3 ± 0.4
toluene-*d*_8_	–1.7 ± 0.2	–5.1 ± 0.7	–0.2 ± 0.3

a Thermodynamic parameters for conversion of the open form to the double-helical form calculated by variable-temperature ^1^H NMR of [(**1b**)_2_Zn][OTf]_2_.

b Δ*G*_open→helix_ at 298 K.

### Chirality Switching of Monometallofoldamers

**Figure 3:**
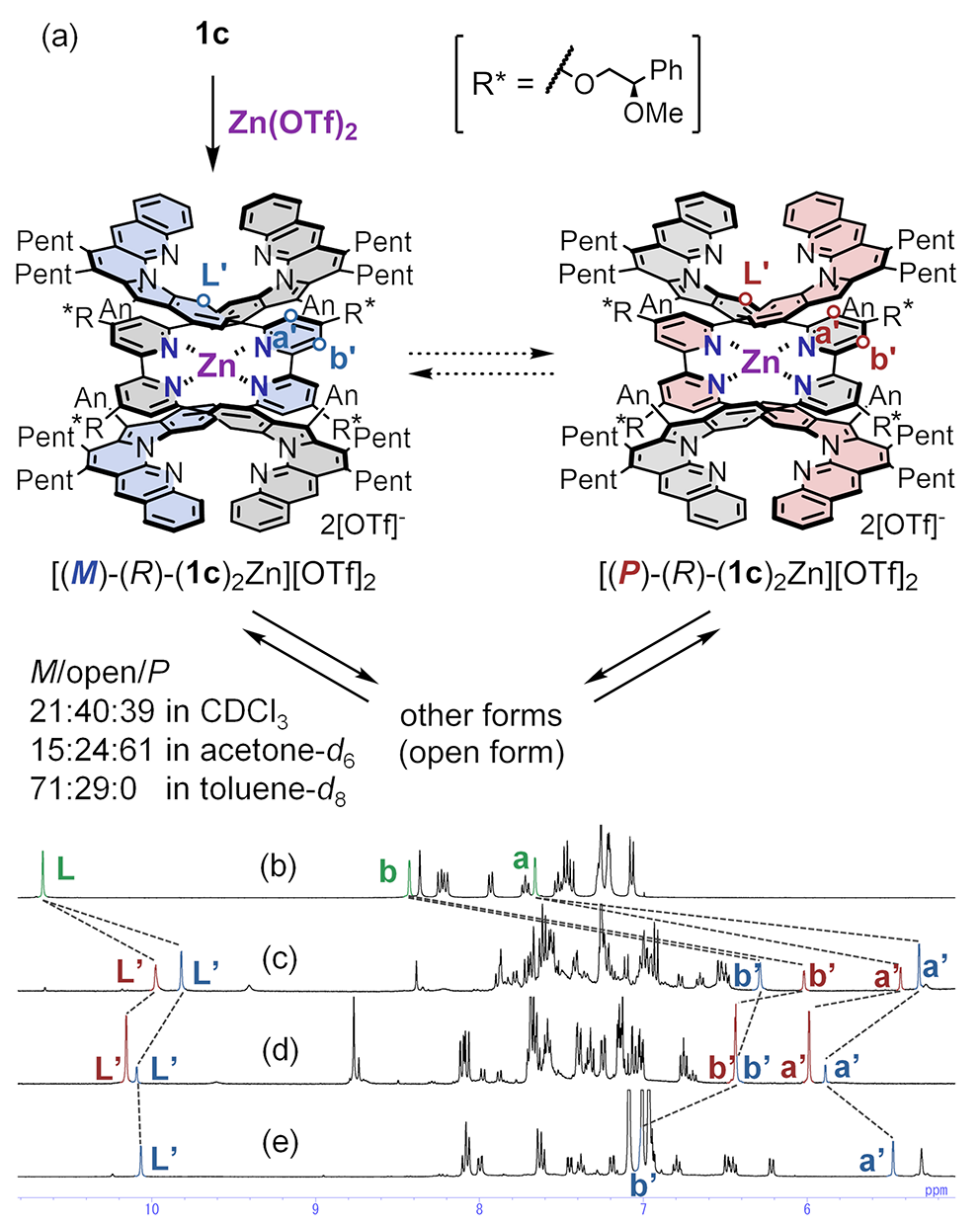
(a) Complexation of **1c** with Zn(OTf)_2_, the equilibrium between *M*-double-helical form, *P*-double-helical form, and other forms (open forms) and ^1^H NMR spectra of (b) **1c** in CDCl_3_, (c) monometallofoldamer [(**1c**)_2_Zn][OTf]_2_ in CDCl_3_, (d) in acetone-*d*_6_, and (e) in toluene-*d*_8_ (1.0 mM, 298 K).

The monometallofoldamers were kinetically stable and not in intercomplex equilibrium (or were in very slow intercomplex equilibrium), as revealed in the ligand exchange experiment (see Supporting Information, Section 5). This suggested that the switching property between the double-helical form and the open forms could be applied to *M*/*P* helicity switching via open forms as intermediate structures (Figure 3). Therefore, the control of the chirality of the monometallofoldamer was investigated by using [(**1c**)_2_Zn][OTf]_2_ with chiral auxiliaries containing the diastereomeric pair [(*M*)-(*R*)-(**1c**)_2_Zn][OTf]_2_ and [(*P*)-(*R*)-(**1c**)_2_Zn][OTf]_2_ in solution. Notably, since the helical structure of the double-helical complex [(**1b**)_2_Zn][OTf]_2_ was affected by the solvent, as discussed above, the helicity of [(**1c**)_2_Zn][OTf]_2_ could also be controlled by solvents.

In CDCl_3_, the double-helical form of [(*R*)-(**1c**)_2_Zn][OTf]_2_ gave two sets of peaks derived from two diastereomers of the double-helical form, [(*M*)-(*R*)-(**1c**)_2_Zn][OTf]_2_ and [(*P*)-(*R*)-(**1c**)_2_Zn][OTf]_2_ (Figure 3). In addition, 40% of the open forms existed in CDCl_3_ (the ratio of the open forms was obtained using 1,1,2,2-C_2_H_2_Cl_4_ as an internal standard due to the too-broad peaks of the open forms at 298 K) (Figures 3b and S67). Interestingly, the equilibrium ratio between *M*/*P* diastereomers significantly depended on the solvent. In CDCl_3_, the equilibrium ratio bias was small (39:21 and 40% of open forms). In contrast, in acetone-*d*_6_, one diastereomer predominated (61:15, and 24% of open forms), and in toluene-*d*_8_, the diastereomer with opposite helicity was exclusive (∼0:71, and 29% of open forms) (Figures 3c–e and S67–69). We assigned two sets of peaks ((*M*)-helical form shown in blue and (*P*)-helical form shown in red in Figure 3c–e) based on the helicity preferences revealed from CD measurements and time-dependent density functional theory (TD-DFT) calculations described below.

**Figure 4:**
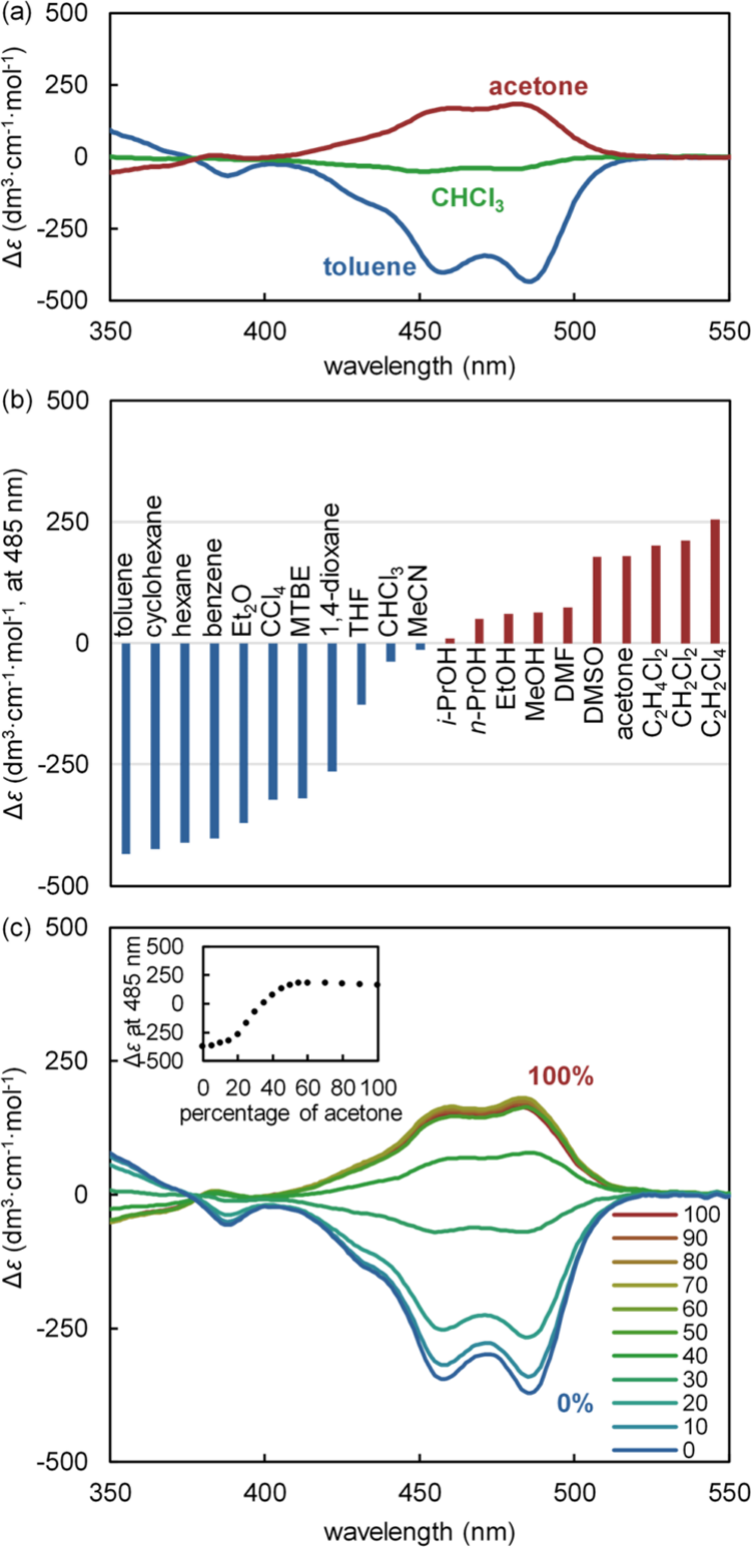
CD spectra of monometallofoldamer [(*R*)-(**1c**)_2_Zn][OTf]_2_ (r.t., [(*R*)-(**1c**)_2_Zn][OTf]_2_ = 5.0 μM) (a) in acetone (red line), in CHCl_3_ (green line), in toluene (blue line); (b) Δε at 485 nm of [(*R*)-(**1c**)_2_Zn][OTf]_2_ in various solvents; (c) CD spectra of [(*R*)-(**1c**)_2_Zn][OTf]_2_ in acetone/toluene mixed solvent (r.t., [(*R*)-(**1c**)_2_Zn][OTf]_2_ = 5.0 μM); inset: plot of percentage by volume in acetone/toluene (vol%).

The diastereomeric bias was investigated by CD measurements (Figure 4 and Table 2). In the 250–600 nm region, the strand (*R*)-**1c** showed a marginal Cotton effect (Figures S30–S33), whereas [(*R*)-(**1c**)_2_Zn][OTf]_2_ exhibited a large Cotton effect, attributed to the double-helical structure (Figure 4a). In the 400–530 nm region, a negative Cotton effect was observed in toluene, whereas a positive Cotton effect was observed in acetone. In CHCl_3_, only a small negative Cotton effect was observed. These results were consistent with the reversed diastereomeric bias observed in the ^1^H NMR spectra.^51^ TD-DFT calculations for model complexes [(*M*)-(**1d**)_2_Zn]^2+^ and [(*P*)-(**1d**)_2_Zn]^2+^ (with R = OMe and methyl groups instead of pentyl groups) attributed the negative Cotton effect to the (*M*)-double helix and the positive Cotton effect to the (*P*)-double helix (Figure S65). Therefore, the preference for (*M*)-helicity in toluene and (*P*)-helicity in acetone was suggested. There was no significant difference in the conformational preference of [(**1c**)_2_Zn][OTf]_2_ in the concentration range of 100 μM to 1 mM by ^1^H NMR spectroscopy (Figures S70 and S71) and 5 μM to 100 μM by CD spectroscopy (Figure S61). The temperature dependence of [(**1c**)_2_Zn][OTf]_2_ was investigated by CD spectroscopy and revealed that the Cotton effect decreases at higher temperatures, probably due to an increase in the ratio of the open forms (Figure S60).

**Table 2:** Molar Circular Dichroism Δε and Dissymmetry Factors *g*_abs_ at 485 nm of Monometallofoldamer [(*R*)-(**1c**)_2_Zn][OTf]_2_ in Various Solvents

solvent	λ_max_ (nm)	Δε (dm^3^·cm^–1^·mol^–1^)^a^	*g*_abs_ (10^–3^)^a^
toluene	458, 485	–434	–9.7
cyclohexane	458, 486	–424	–12
hexane	457, 485	–411	–12
benzene	458, 485	–402	–10
Et_2_O	456, 484	–371	–10
CCl_4_	457, 485	–322	–8.2
MTBE	456, 483	–320	–9.1
1,4-dioxane	456, 483	–264	–6.8
THF	456, 483	–126	–3.3
CHCl_3_	452, 479	–38	–0.91
MeCN	450, 478	–14	–0.50
*i*-PrOH	464, 496	10	0.28
*n*-PrOH	464, 491	51	1.6
EtOH	465, 490	61	1.8
MeOH	462, 487	64	2.2
DMF	461, 485	74	2.0
DMSO	462, 484	178	4.7
acetone	460, 482	179	5.3
1,2-C_2_H_4_Cl_2_	462, 486	202	5.3
CH_2_Cl_2_	462, 486	211	5.0
1,1,2,2-C_2_H_2_Cl_4_	462, 487	255	6.1

a Δε and *g*_abs_ (=Δε/ε) were measured at 485 nm.

The diastereomeric *M*/*P*-helix pair was expected to invert the apparent equilibrium via the open forms depending on the solvation to the double-helical form. In fact, (*M*)-helicity was favored in nonpolar solvents [e.g., hexane, cyclohexane, benzene, toluene, Et_2_O, MTBE (*t*-BuOMe)], and (*P*)-helicity was favored in Lewis basic solvents (e.g., acetone, DMSO) (Figure 4b, Table 2, and Figures S34 and S35). Acetone and DMSO exhibited a large positive Cotton effect, suggesting that carbonyl and sulfoxide (C=O, S=O) groups were biased toward (*P*)-helicity. Alcohols (R–OH) were also biased toward (*P*)-helicity (Figure 4b, Table 2, and Figure S36).

Interestingly, halogenated solvents exhibited remarkable differences in the *M*/*P* preferences. (*M*)-helicity was favored in CCl_4_ and CHCl_3_, but (*P*)-helicity was increasingly favored in 1,1,2,2-C_2_H_2_Cl_4_, CH_2_Cl_2_ and 1,2-C_2_H_4_Cl_2_ in that order (Figure 4b, Table 2, and Figure S37). These results indicate that the (*P*)-helicity was stabilized depending on Lewis basicity, which was consistent with the trend in cation solvation capacity reported by Swain et al. (CCl_4_: 0.34; CHCl_3_: 0.73; 1,2-C_2_H_4_Cl_2_: 0.82; CH_2_Cl_2_: 0.82; and 1,1,2,2-C_2_H_2_Cl_4_: n/a).^52^ In contrast to acetone and DMSO, CH_3_CN and DMF exhibited limited effects on the preference for (*P*)-helicity, affording a relatively equal diastereomeric ratio (*M*/*P*) (Figure 4b and Table 2). These results demonstrated that the helicity of the double-helical complex [(*R*)-(**1c**)_2_Zn][OTf]_2_ with a chiral side chain can be controlled based on the Lewis basicity of achiral solvents. In fact, reversible switching of the chiral properties by solvents was demonstrated in acetone/toluene (Figures 4c and S59). The plot of Δε against the ratio of acetone/toluene showed an S-shaped nonlinear curve, suggesting that acetone molecules are solvating cooperatively.^50,53,54^

Examples of chirality switching induced by achiral solvents in small molecules are limited.^28,29,32^ In terms of chiral information output, to the best of our knowledge, the monometallofoldamer [(*R*)-(**1c**)_2_Zn][OTf]_2_ exhibited one of the largest solvent-responsive Δε inversions in small molecules, probably due to the stability of the double-helical structure with metal bridging and dense π-stacking. Furthermore, the intensity of *g*_abs_ (= Δε/ε ∼ −1.2 × 10^–2^ to +6.1 × 10^–3^ at 485 nm) in [(*R*)-(**1c**)_2_Zn][OTf]_2_ was comparable to or greater than that of representative switchable helical polymers reported by Suginome et al.^21−23^ (*g*_abs_ ∼ −3 × 10^–3^ to +2.5 × 10^–3^).

### Mechanism of Helicity Inversion

**Scheme 6:**
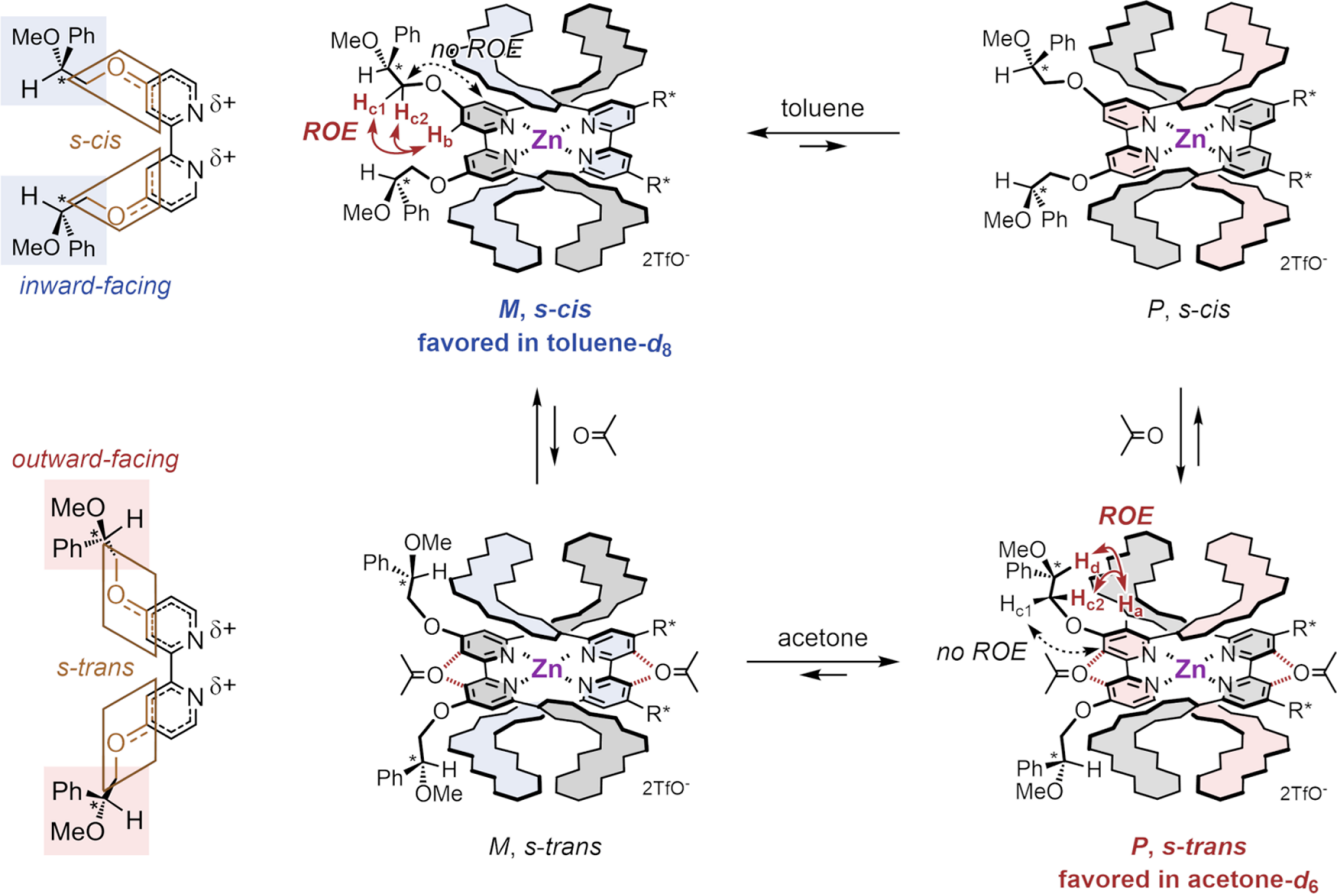
Solvent-Dependent Conformational Change of the Chiral Side Chains and Helicity Preference of [(*R*)-(**1c**)_2_Zn][OTf]_2_ The probable conformations of monometallofoldamer [(*R*)-(**1c**)_2_Zn][OTf]_2_ based on ROESY data.

Measurement of ROESY spectra in order to investigate the origin of helicity preference of [(*R*)-(**1c**)_2_Zn][OTf]_2_ revealed that a solvent-dependent conformational change of the chiral side chains was important for the preference (Scheme 6, Figures S73 and S75). In toluene-*d*_8_ (in which [(*M*)-(*R*)-(**1c**)_2_Zn][OTf]_2_ was favored), the protons H_b_ at the 3,3′-position of the bipyridine spacers correlated with the protons H_c1_ and H_c2_ of the chiral side chains, suggesting the *s*-*cis* conformation with inward-facing chiral side chains (Figure S73). The δ+ charge on the bipyridines due to complexation with a cation induced a double bonding character to the C–O bonds of the chiral side chains, resulting in the fixation of the conformation of the chiral side chains. On the other hand, in acetone-*d*_6_ (in which [(*P*)-(*R*)-(**1c**)_2_Zn][OTf]_2_ was favored), the protons H_a_ at the 5,5′-position of the bipyridine spacers correlated with the protons H_c2_ and H_d_ of the chiral side chains, suggesting the *s*-*trans* conformation with outward-facing chiral side chains (Figure S75). The cooperative interaction of acetone molecules with the bipyridyl protons H_b_ might induce a conformational reversal of the chiral side chains to *s*-*trans*. Furthermore, VT NMR and the van’t Hoff plot revealed that the (*P*)-helix was enthalpically stabilized in acetone-*d*_6_, although it was entropically unstabilized [Δ*H_M_*_→_*_P_* = −1.2 ± 0.2 kcal·mol^–1^, Δ*S_M_*_→_*_P_* = −1.5 ± 0.8 cal·mol^–1^·K^–1^, Δ*G_M_*_→_*_P_* = −0.8 ± 0.5 kcal·mol^–1^] (Figures S76 and 77). The enthalpic preference of the (*P*)-helicity in acetone-*d*_6_ showed that stabilization by solvation was more effective for the (*P*)-helicity than for the (*M*)-helicity, probably due to the additional CH−π interactions between L-shaped units and chiral side chains.

The effects of the conformation of the side chains on the preference of the helix sense are summarized as follows. In toluene, the chiral side chains were directed inward, and [(*M*)-(*R*)-(**1c**)_2_Zn][OTf]_2_ was favored. Interactions such as hydrogen bonds and CH−π interactions between the methoxy or phenyl groups of the side chains and the double helix could stabilize the (*M*)-helicity. Experimentally, protons located on the outside of the double helix shifted upfield in toluene-*d*_8_, indicating CH−π interactions between phenyl groups of chiral side chains and the double helix (Figures S72 and S73). On the other hand, in acetone, the chiral side chains were directed outward, and the (*P*)-helicity was enthalpically favored. The conformational change of the chiral side chains by interaction with acetone could cause a reversal of both the polarity and the interaction with the double helix of the side chain, resulting in stabilization of the (*P*)-helicity.

### Chiral Transfer and Amplification in Monometallofoldamers

**Figure 5:**
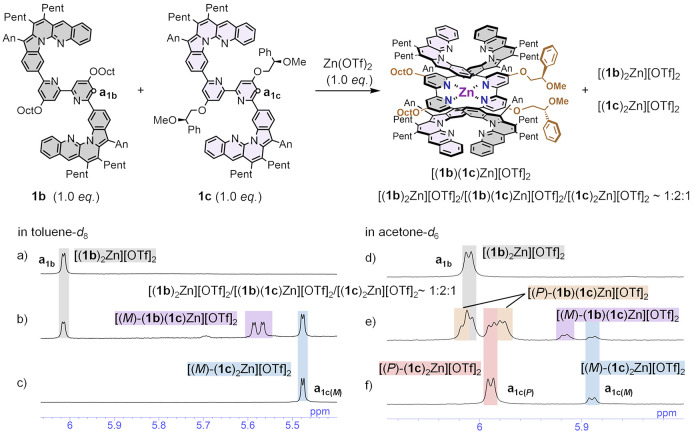
Preparation of hetero complex [(**1b**)(**1c**)Zn][OTf]_2_ and ^1^H NMR spectra (5.4–6.0ppm) of (a) [(**1b**)_2_Zn][OTf]_2_, (b) mixture [(**1b**)_2_Zn][OTf]_2_/[(**1b**)(**1c**)Zn][OTf]_2_/[(**1c**)_2_Zn][OTf]_2_ ∼ 1:2:1, and (c) [(**1c**)_2_Zn][OTf]_2_ in toluene-*d*_8_ at 298 K; ^1^H NMR spectra (5.8–6.0 ppm) of (d) [(**1b**)_2_Zn][OTf]_2_, (e) [(**1b**)_2_Zn][OTf]_2_/[(**1b**)(**1c**)Zn][OTf]_2_/[(**1c**)_2_Zn][OTf]_2_ ∼ 1:2:1, and (f) [(**1b**)_2_Zn][OTf]_2_ in acetone-*d*_6_ at 298 K ([(**1b**)_2_Zn][OTf]_2_ + [(**1b**)(**1c**)Zn][OTf]_2_ + [(**1c**)_2_Zn][OTf]_2_ = 1.0 mM).

Cooperativity and information transfer between two strands are the most remarkable features of double helices. Therefore, the transfer of chirality from one strand to the other in the double-helical monometallofoldamers was examined. Upon addition of Zn(OTf)_2_ to a mixture of **1b** and **1c** (**1b**/**1c** = 1:1), a mixture of [(**1b**)_2_Zn][OTf]_2_, [(**1b**)(**1c**)Zn][OTf]_2_, and [(**1c**)_2_Zn][OTf]_2_ was kinetically produced in a ratio of 1:2:1 (Figure 5). The diastereomeric ratio of heteroleptic complex [(**1b**)(**1c**)Zn][OTf]_2_ in the mixture was investigated by ^1^H NMR spectroscopy in toluene-*d*_8_ and acetone-*d*_6_ (Figure 5). In toluene, the heteroleptic complex [(**1b**)(**1c**)Zn][OTf]_2_ exhibited only one set of peaks from one diastereomer (consisting of 2-fold peaks derived from **1b** and **1c** compared to the homoleptic complex), suggesting [(*M*)-(**1b**)(**1c**)Zn][OTf]_2_ with (*M*)-helicity to the achiral strands was exclusive, similar to the homoleptic complex [(**1c**)_2_Zn][OTf]_2_ (Figures 5a–c and S78). The exclusive bias favoring the (*M*)-helicity indicated that the chirality of chiral strand **1c** has been completely transferred to achiral strand **1b**. In acetone, one diastereomer [(*P*)-(**1b**)(**1c**)Zn][OTf]_2_ with (*P*)-helicity was prevalent, probably biased toward (*P*)-helicity, similar to the homoleptic complex [(**1c**)_2_Zn][OTf]_2_ (Figures 5d–f and S79).

**Figure 6:**
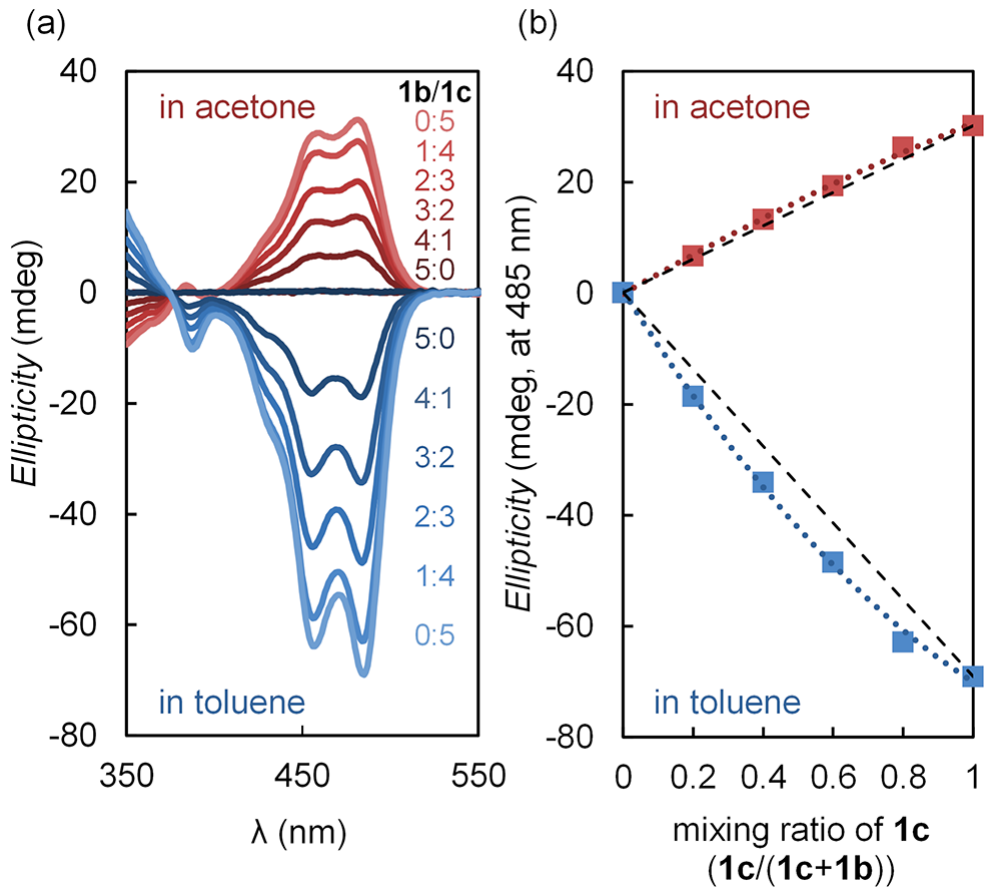
(a) CD spectra upon complexing **1b** and **1c** with Zn(OTf)_2_ in various ratios of toluene and acetone; ([(**1b**)_2_Zn][OTf]_2_ + [(**1b**)(**1c**)Zn][OTf]_2_ + [(**1c**)_2_Zn][OTf]_2_) = 5.0 μM, r.t., in acetone (shown in red) and in toluene (shown in blue); (b) CD intensities at 485 nm in mixtures of [(**1b**)_2_Zn][OTf]_2_/[(**1b**)(**1c**)Zn][OTf]_2_/[(**1c**)_2_Zn][OTf]_2_ at various ratios of **1b**/**1c**.

**Figure 7:**
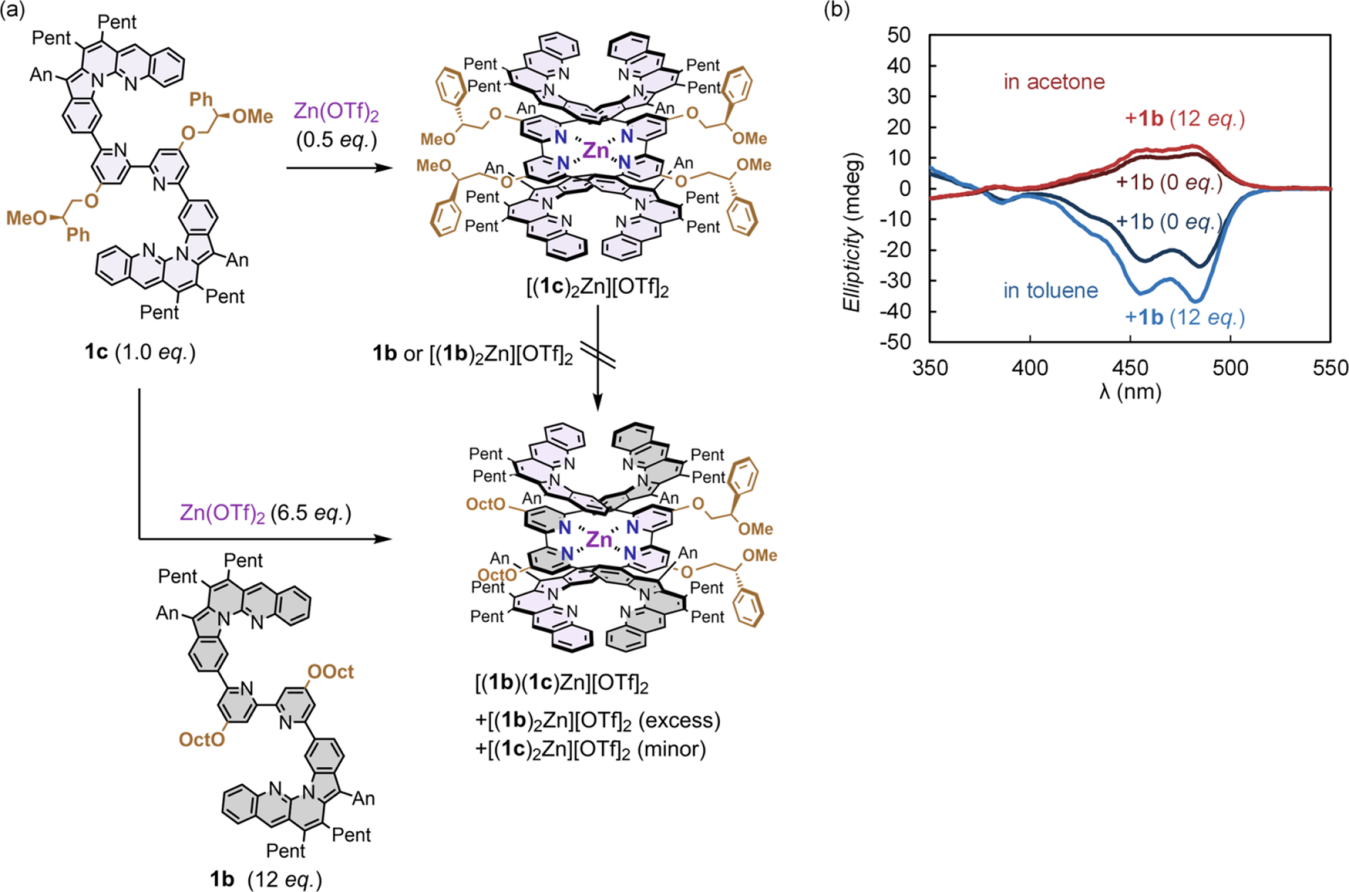
(a) Scheme of chirality amplification by formation of heteroleptic complexes; (b) CD spectra of [(*R*)-(**1c**)_2_Zn][OTf]_2_ and a mixture of [(**1b**)_2_Zn][OTf]_2_, [(**1b**)(**1c**)Zn][OTf]_2_, and [(**1c**)_2_Zn][OTf]_2_ in acetone (shown in red) and in toluene (shown in blue) (r.t., the concentration of ligand **1c** was 4 μM).

Information transfer and replication between two strands in double helices are central dogmas in nature. The exclusive formation of the chiral heteroleptic complex with achiral strands observed in toluene suggested the possibility of chiral transfer^15,18,21,55^ and amplification.^100^ Therefore, we measured and compared the nonlinearity in CD spectra of mixtures of homoleptic and heteroleptic complexes prepared with different ratios of **1b** and **1c** (Figure 6). No Cotton effect was observed under conditions where only [(**1b**)_2_Zn][OTf]_2_ was present (Figure 6, **1b**/**1c** = 5:0), while the Cotton effect increased with the addition of **1c**, due to the increase in the ratio of [(**1b**)(**1c**)Zn][OTf]_2_ and [(**1c**)_2_Zn][OTf]_2_ (Figure 6, **1b**/**1c** = 4:1–1:4). Although the plot in acetone was nearly linear, the plot in toluene was nonlinear, indicating that both [(**1c**)_2_Zn][OTf]_2_ and [(**1b**)(**1c**)Zn][OTf]_2_ produced with statistical probability were biased toward (*M*)-helicity. This result could be regarded as the nonlinear chiral amplification, such as in the sergeants-and-soldiers principle.^15,21,56,57^ In other words, this allows chiral amplification of up to 2 equiv of chiral double helices from 1 equiv of chiral strands by adding excess achiral strands. We subsequently demonstrated such chiral amplification upon adding 12 equiv of **1b** and Zn(OTf)_2_ to 1 equiv of **1c**. The CD spectra of the mixture of homoleptic and heteroleptic complexes in toluene exhibited an amplified Cotton effect compared with that of the complexes under the conditions without **1b** (Figures 7b and S81–S83). The increase in Cotton effects was 1.4 times greater in toluene and 1.2 times greater in acetone, indicating a clear chiral amplification due to the formation of a heteroleptic complex. Chiral induction to an achiral strand and solvent-dependent helicity inversion in these heteroleptic complexes of [(**1b**)(**1c**)Zn][OTf]_2_ will provide important insights for more efficient and complex control of chiral information beyond that in single helices.

## Conclusions

In conclusion, double-helical monometallofoldamers [(**1**)_2_Zn][OTf]_2_ were synthesized from bipyridine-type strands **1a**–**c** with L-shaped dibenzopyrrolo[1,2-*a*][1,8]naphthyridine units by complexation with a Zn(II) cation. X-ray crystallography revealed double-helical structures with L-shaped units densely stacked by π–π interactions and enclosing a metal cation. The stimuli-responsive switchability of the monometallofoldamers was investigated, and these complexes were found to be in equilibrium between the double-helical form favored at low temperatures and the open forms favored at high temperatures. Interestingly, the helix sense of [(**1c**)_2_Zn][OTf]_2_ with chiral side chains can be controlled in response to achiral solvent stimuli, where the *M*/*P* helicity switching is induced by conformational changes of the chiral side chains due to the interaction of Lewis basic solvents to double helices. In addition, in the chiral heteroleptic double-helical monometallofoldamer [(**1b**)(**1c**)Zn][OTf]_2_, which was produced from the achiral strand **1b** and the chiral strand **1c**, the switchable chiral properties of the chiral strand were transferred and amplified through the information transfer capability of the double helix. These double-helical monometallofoldamers would offer novel design guidelines for switching significant chiral properties and higher-order chiral structure controls as in nature and would facilitate the development of new chiroptical switching materials.

## Supporting Information Available

Experimental procedures; NMR spectra; UV–vis and CD spectra; theoretical studies; and X-ray crystallography data (PDF)
