# Supplementary material for: M/P Helicity Switching and Chiral Amplification in Double-Helical Monometallofoldamers
Source: J Am Chem Soc. 2024 Jul 19;146(30):21078–88. doi: 10.1021/jacs.4c06560 (PMC11295176; doi:10.1021/jacs.4c06560)
Supplement: Supplementary file 1 — ja4c06560_si_001.pdf [file ja4c06560_si_001.pdf]

Supporting Information for

# ***M*/*P* Helicity Switching and Chiral Amplification in Double-Helical Monometallofoldamers**

Kotaro Matsumura, Keigo Kinjo, Kotaro Tateno, Kosuke One, Yoshitaka Tsuchido and Hidetoshi Kawai*

## Contents


1. General 1

2. Experimental section 2

3. Complexation of strands with a Zn(II) cation 11

4. Single crystal X-ray structural analysis 15

5. Kinetic stability of monometallofoldamers and intercomplex equilibrium 20

6. Investigation of conformational isomers of [(**1b**)$_2$Zn][OTf]$_2$ by EXSY and DOSY 25

7. VT NMR and van't Hoff plots for [(**1b**)$_2$Zn][OTf]$_2$ 29

8. UV-vis and CD spectra of (*R*)-(**1c**) and [(*R*)-(**1c**)$_2$Zn][OTf]$_2$ 42

9. Time dependence of CD changes from (*M*)-helicity to (*P*)-helicity 54

10. Theoretical calculations 58

11. NMR study ($^1$H NMR, 2D COSY and ROESY) for [(*R*)-(**1c**)$_2$Zn][OTf]$_2$ 59

12. van't Hoff plot for the equilibrium between (*M*) and (*P*)-double-helical forms of [(*R*)-(**1c**)$_2$Zn][OTf]$_2$ 67

13. Chiral amplification via heteroleptic monometallofoldamer [(**1b**)(**1c**)Zn][OTf]$_2$ 69

14. $^1$H and $^{13}$C NMR spectra of new compounds 74

15. References 85

# 1. General

$^1$H and $^{13}$C NMR and ROESY spectra were recorded on a Bruker-Biospin·AVANCE NEO 400 ($^1$H: 400 MHz, $^{13}$C: 100 MHz) spectrometer. IR spectra were taken on a JASCO FT/IR-4600 (ATR). HRMS analyses were performed on a JEOL JMS-S3000 SpiralTOF (MALDI-TOF) mass spectrometer. UV-vis absorption spectra were obtained on a JASCO V-630 spectrophotometer, and CD spectra was taken on JASCO J-820 at room temperature. All melting points were determined on a METTLER TOLEDO MP90. The X-ray analysis data were obtained by using a Rigaku XtaLAB Synergy-DW diffractometer. Column chromatography was performed on silica gel (FUJI SILYSIA CHEMICAL LTD., CHROMATOREX PSQ60B). GPC purification was carried out on LC-9210 NEXT with JAIGEL-2HH+3HH columns eluted with $CHCl_3$ with 0.5% triethylamine. Optical rotation was taken on JASCO·P-1010-Polarimeter.

Tris{5-(4-methoxyphenyl)-6,7-dibenzopyrrolo[1,2-*a*][1,8]naphthyridin-2-yl}boroxine **2**[S1] and 5-bromo-6,7-dipentyldibenzopyrrolo[1,2-*a*][1,8]naphthyridine **6**[S2] were prepared according to the reported procedures. 6,6'-Dibromo-2,2'-bipyridyl **3a** was purchased from Tokyo Chemical Industry Co. and (*R*)-(-)-2-methoxy-2-phenylethanol was purchased from Sigma-Aldrich Co. LLC.

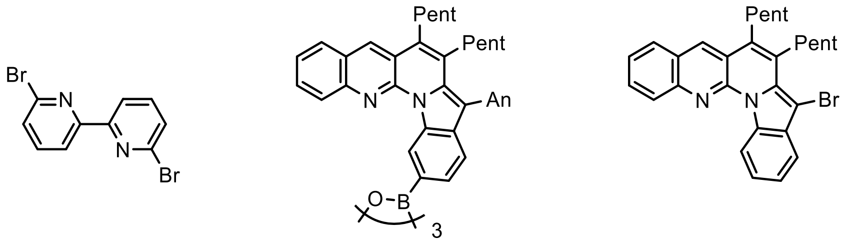


**3a** **2** **6**

**Chart S1.** Known compounds.

## 2. Experimental section

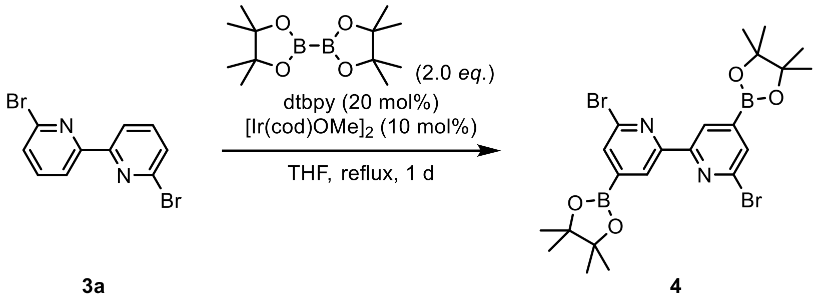

**6,6'-dibromo-4,4'-bis(4,4,5,5-tetramethyl-1,3,2-dioxaborolan-2-yl)-2,2'-bipyridine 4:** A mixture of 6,6'-dibromo-2,2'-bipyridyl **3a** (220 mg, 0.70 mmol), bis(pinacolato)diboron (356 mg, 1.4 mmol), 4,4'-di-*tert*-butyl-2,2'-bipyridyl (dtbbpy, 38 mg, 0.14 mmol) and (1,5-cyclooctadiene)(methoxy)iridium(I) dimer (46 mg, 70 μmol) in THF (2 mL) was heated to reflux for 1 d. After the reaction mixture was cooled to room temperature, it was concentrated *in vacuo.* The residue was washed hexane to give crude 6,6'-dibromo-4,4'-bis(4,4,5,5-tetramethyl-1,3,2-dioxaborolan-2-yl)-2,2'-bipyridine **4** (406 mg) as a brown solid which was used without further purification:

Mp 288.6-298.9 $^{0}$C (decomp.); IR (ATR): 3078, 2978, 2937, 2873, 1614, 1554, 1520, 1469, 1452, 1400, 1358, 1338, 1271, 1213, 1140, 1082, 964, 860, 723, 677 cm$^{-1}$; $^{1}$H NMR (400 MHz, $CDCl_3$) $\delta$: 8.63 (s, 2H), 7.85 (s, 2H), 1.37 (s, 24H) ppm; $^{13}$C NMR (100 MHz, $CDCl_3$) $\delta$: 155.4, 141.7, 133.6, 125.2, 84.94, 24.9 ppm; HR-MS (MALDI: matrix DCTB) (*m*/*z*): $[M+Na]^+$ calcd for $C_{22}H_{22}B_2N_2O_4Br_2$+Na: 587.0494, found 587.0513.

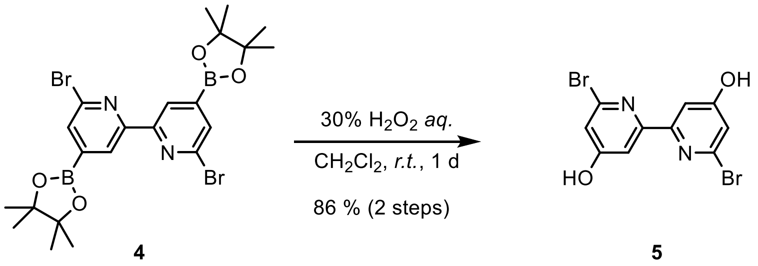

**6,6'-dibromo-4,4'-dihydroxy-2,2'-bipyridine 5:** A mixture of 6,6'-dibromo-4,4'-bis(4,4,5,5-tetramethyl-1,3,2-dioxaborolan-2-yl)-2,2'-bipyridine **4** (406 mg, 0.70 mmol) in a mixture of 30% $H_2O_2$ *aq.* (1 mL) and $CHCl_3$ (30 mL) was stirred at room temperature for 1 d. After the reaction mixture was added EtOAc and 10% $NaHSO_3$ *aq.*. The aqueous phase was extracted with EtOAc and the combined organic phases were washed with 10% $NaHSO_3$ *aq.* and brine. The organic phase was dried over $MgSO_4$ and concentrated in *vacuo*. The residue was dissolved in EtOAc (5 mL) and added hexane (25 mL). The precipitates were collected by suction filtration and washed with hexane to give 6,6'-dibromo-4,4'-dihydroxy-2,2'-bipyridine **5** (209 mg, 86%, 2 steps) as an off-white solid:

Mp 230.5-232.3 °C; IR (ATR): 3274, 3089, 2903, 2841, 1610, 1554, 1502, 1456, 1431, 1399, 1336, 1316, 1283, 1269, 1248, 1207, 1146, 1399, 1336, 1316, 1283, 1269, 1248, 1207, 1146, 1115, 1064, 1011, 984, 918, 836, 703, 548, 520 cm$^{-1}$; $^{1}$H NMR (400 MHz, Acetone-$d_6$) $\delta$: 10.07 (br, 2H), 7.85 (d, $J$ = 2.0 Hz, 2H), 7.09 (d, $J$ = 2.0 Hz, 2H) ppm; $^{13}$C NMR (100 MHz, Acetone-$d_6$) $\delta$: 167.3, 157.2, 142.9, 116.2, 109.3 ppm; HR-MS (MALDI: matrix dithranol) (*m*/*z*): $[M+H]^+$ calcd for $C_{10}H_6Br_2N_2O_2$+H: 344.8869, found 344.8868.

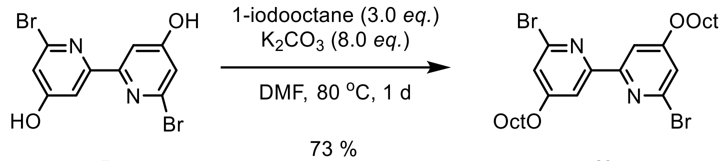


**6,6'-dibromo-4,4'-dioctoxy-2,2'-bipyridine 3b:** A mixture of 6,6'-dibromo-4,4'-dihydroxy-2,2'-bipyridine **5** (21 mg, 60 μmol), 1-iodooctane (32 μL, 0.18 mmol) and $K_2CO_3$ (66 mg, 0.48 mmol) in DMF (0.3 mL) was heated at 80 °C for 2 d. After the reaction mixture was cooled to room temperature, it was added MTBE and water. The aqueous phase was extracted with MTBE and the combined organic phases were washed with water and brine. The organic phase was dried over $MgSO_4$ and concentrated *in vacuo*. The residue was purified by silica gel column chromatography (hexane/$CHCl_3$ = 1:1 to hexane/$CHCl_3$/TEA = 50:50:1) to give 6,6'-dibromo-4,4'-dioctoxy-2,2'-bipyridine **3b** (25 mg, 73%) as a white solid:

Mp 76.6-77.1 °C; IR (ATR): 3097, 2952, 2922, 2852, 2725, 1576, 1545, 1471, 1417, 1390, 1369, 1309, 1271, 1263, 1146, 1084, 1063, 1036, 1003, 889, 843, 706 $cm^{-1}$; $^{1}H$ NMR (400 MHz, $CDCl_3$) $\delta$: 7.88 (d, $J$ = 2.2 Hz, 2H), 6.99 (d, $J$ = 2.2 Hz, 2H), 4.09 (t, $J$ = 6.4 Hz, 4H), 1.81 (qn, $J$ = 6.6 Hz, 4H), 1.46 (qn, $J$ = 7.1 Hz, 4H), 1.40-1.24 (m, 16H), 0.90 (t, $J$ = 6.8 Hz, 6H) ppm; $^{13}C$ NMR (100 MHz, $CDCl_3$) $\delta$: 167.3, 156.4, 142.1, 114.4, 107.6, 68.8, 31.8, 29.2, 29.2, 28.8, 25.9, 22.6, 14.1 ppm; HR-MS (MALDI: matrix dithranol) (*m*/*z*): $[M+Na]^+$ calcd for $C_{26}H_{38}Br_2N_2O_2$+Na: 591.1192, found 591.1193.

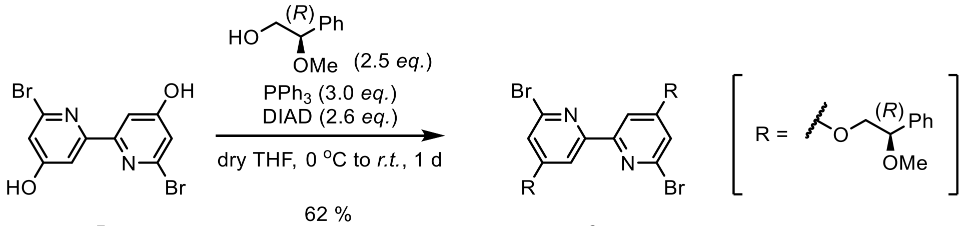


**6,6'-dibromo-4,4'-bis((*R*)-2-methoxy-2-phenylethoxy)-2,2'-bipyridine 3c:** A mixture of 6,6'-dibromo-4,4'-dihydroxy-2,2'-bipyridine **5** (34 mg, 0.10 mmol), (*R*)-(-)-2-methoxy-2-phenylethanol (36 μL, 0.25 mmol) and $PPh_3$ (79 mg, 0.30 mmol) in dry THF (0.5 mL) was cooled to 0 °C, and DIAD was added dropwise for 5 min. The reaction mixture was warmed to room temperature and stirred for 1 d. After the reaction mixture concentrated *in vacuo*. The residue was purified by silica gel column chromatography ($CHCl_3$) to give 6,6'-dibromo-4,4'-bis((*R*)-2-methoxy-2-phenylethoxy)-2,2'-bipyridine **3c** (38 mg, 62%) as a white solid:

Mp 137.8-138.3 °C; IR (ATR): 3060, 3030, 2952, 2926, 2868, 1577, 1557, 1485, 1450, 1396, 1308, 1282, 1242, 1173, 1108, 1058, 1033, 833, 748, 700, 559 $cm^{-1}$; $^{1}H$ NMR (400 MHz, $CDCl_3$) $\delta$: 7.88 (d, $J$ = 2.2 Hz, 2H), 7.45-7.32 (m, 10H), 7.01 (d, $J$ = 2.2 Hz, 2H), 4.59 (dd, $J$ = 7.8, 3.6 Hz, 2H), 4.27 (dd, $J$ = 10.2, 7.8 Hz, 2H), 4.13 (dd, $J$ = 10.2, 3.6 Hz, 2H), 3.35 (s, 6H) ppm; $^{13}C$ NMR (100 MHz, $CDCl_3$) $\delta$: 166.8, 156.3, 142.1, 137.6, 128.8, 128.6, 127.0, 114.8, 107.4, 81.7, 72.5, 57.2 ppm; HR-MS (MALDI: matrix dithranol) (*m*/*z*): $[M+Na]^+$ calcd for $C_{28}H_{26}Br_2N_2O_4$+Na: 635.0152, found 635.0151; $[\alpha]_D^{27}$=-25.0 ($c$=0.139, Acetone).

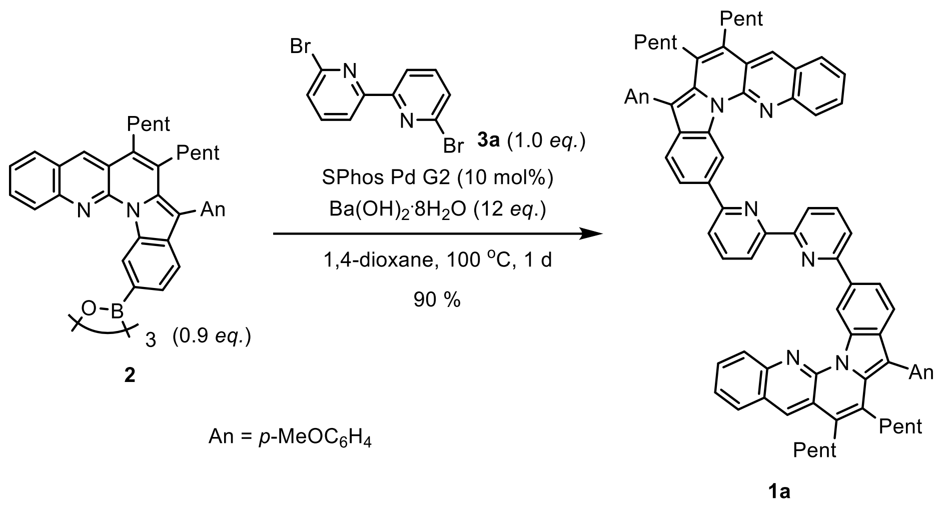


**2,2’-{(2,2’-bipyridyl)-6,6’-diyl}bis({5-(4-methoxyphenyl)-6,7-dipentyldibenzopyrrolo[1,2-*a*][1,8] naphthyridine}) 1a:** To a mixture of boroxine **2**[S1] (28 mg, 17 μmol), 6,6'-dibromo-2,2'-bipyridyl **3a** (6.3 mg, 50 μmol) in 1,4-dioxane (1 mL) was added SPhos Pd G2 (3.6 mg, 5 μmol) and $Ba(OH)_2 \cdot 8H_2O$ (76 mg, 240 μmol). The mixture was heated at 100 ˚C for 1 d. After the reaction mixture was cooled to room temperature, it was added $CHCl_3$ and water. The aqueous phase was extracted with $CHCl_3$ and the combined organic phases were washed with brine. The organic phase was dried over $MgSO_4$ and concentrated *in vacuo.* The residue was washed with hexane/$CHCl_3$ (4:1) and hexane to give **1a** (21 mg, 90%) as a yellow solid:

Mp 277.4-284.0 ºC (*decomp.*); IR (ATR): 3054, 2952, 2925, 2864, 1610, 1564, 1539, 1483, 1448, 1392, 1335, 1282, 1242, 1174, 1124, 1105, 1036, 793, 746, 650, 590 $cm^{-1}$; $^1$H NMR (400 MHz, $CDCl_3$) $\delta$: 10.84 (d, $J$ = 1.6 Hz, 2H), 8.95 (dd, $J$ = 7.5, 1.1 Hz, 2H), 8.40 (d, $J$ = 8.0 Hz, 2H), 8.38 (s, 2H), 8.24 (dd, $J$ = 8.0, 1.6 Hz, 2H), 8.12 (dd, $J$ = 8.0, 1.1 Hz, 2H), 8.07 (t, $J$ = 7.5 Hz, 2H), 7.95 (dd, $J$ = 7.5, 1.1 Hz, 2H), 7.82 (td, $J$ = 8.0, 1.1 Hz, 2H), 7.57 (td, $J$ = 8.0, 1.0 Hz, 2H), 7.48 (d, $J$ = 8.0 Hz, 4H), 7.47-7.44 (d, 2H), 7.07 (d, $J$ = 8.0 Hz, 4H),3.94 (s, 6H), 2.92 (t, $J$ = 8.2 Hz, 4H), 2.61 (t, $J$ = 8.2 Hz, 4H), 1.73 (qn, $J$ = 8.2 Hz, 4H), 1.62-1.32 (m, 12H), 1.15 (sx, $J$ = 7.5 Hz, 4H), 0.98 (t, $J$ = 7.3 Hz, 6H), 0.86-0.76 (m, 10H) ppm; $^{13}$C NMR could not be measured due to poor solubility of **1a**; HR-MS (MALDI: matrix dithranol) (*m*/*z*): $[M+H]^+$ calcd for $C_{82}H_{80}N_6O_2$+H: 1181.6416, found 1181.6400.

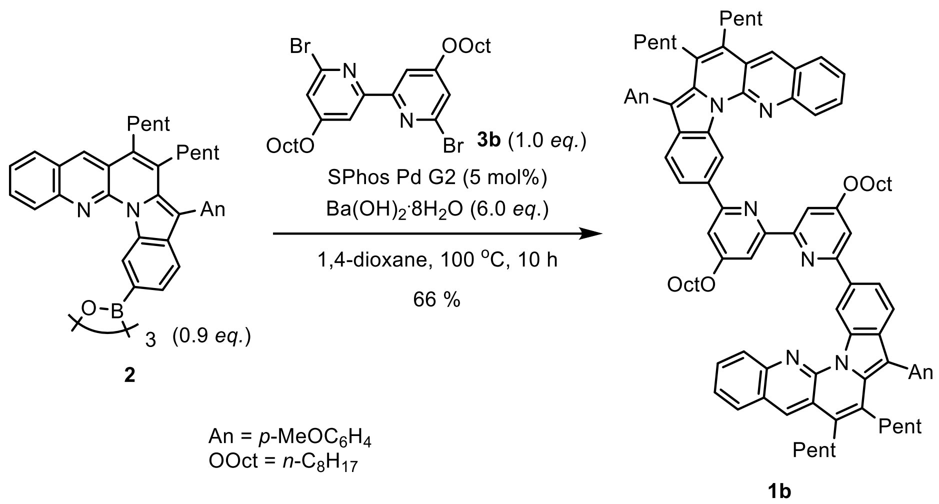


**2,2'-{4,4'-dioctoxy(2,2'-bipyridyl)-6,6'-diyl}bis({5-(4-methoxyphenyl)-6,7-dipentyldibenzopyrrolo[1,2-*a*][1,8] naphthyridine}) 1b:** To a mixture of boroxine **2**[S1] (148 mg, 0.25 mmol) and 6,6'-dibromo-4,4'-dioctoxy-2,2'-bipyridine **3b** (57 mg, 0.10 mmol) in 1,4-dioxane (2 mL) was added SPhos Pd G2 (3.6 mg, 5 μmol) and $Ba(OH)_2 \cdot 8H_2O$ (189 mg, 0.60 mmol). The mixture was heated at 100 ˚C for 10 h. After the reaction mixture was cooled to room temperature, it was added $CHCl_3$ and $NH_4Cl$ *aq.* The aqueous phase was extracted with $CHCl_3$ and the combined organic phases were washed with $NH_4Cl$ *aq.* and brine. The organic phase was dried over $MgSO_4$ and concentrated *in vacuo.* The residue was purified by silica gel column chromatography (hexane/$CHCl_3$ = 1:1 to $CHCl_3$) and obtained solid was washed with EtOH to give **1b** (97 mg, 66%) as an orange solid:

Mp 214.0-216.1 °C (*decomp.*); IR (ATR): 3055, 2952, 2927, 2856, 1577, 1485, 1452, 1396, 1313, 1282, 1244, 1173, 1126, 1109, 1053, 1032, 858, 750 $cm^{-1}$; $^1H$ NMR (400 MHz, $CDCl_3$) $\delta$: 10.76 (d, $J$ = 1.5 Hz, 2H), 8.42 (d, $J$ = 2.2 Hz, 2H), 8.35 (s, 2H), 8.29 (d, $J$ = 8.4 Hz, 2H), 8.20 (dd, $J$ = 8.3, 1.5 Hz, 2H), 7.92 (d, $J$ = 8.4 Hz, 2H), 7.74 (td, $J$ = 8.4, 1.3 Hz, 2H), 7.62 (d, $J$ = 2.2 Hz, 2H), 7.50 (td, $J$ = 8.4, 1.0 Hz, 2H), 7.46 (d, $J$ = 8.7 Hz, 4H), 7.44 (d, $J$ = 8.3 Hz, 2H), 7.06 (d, $J$ = 8.7 Hz, 4H), 4.25 (t, $J$ = 6.7 Hz, 4H), 3.93 (s, 6H), 2.90 (t, $J$ = 7.2 Hz, 4H), 2.60 (t, $J$ = 8.2 Hz, 4H), 1.81 (qn, $J$ = 7.2 Hz, 4H), 1.67-1.76 (m, 4H), 1.46 (sx, $J$ = 7.6 Hz, 4H), 1.42-1.18 (m, 26H), 1.14 (sx, $J$ = 7.3 Hz, 4H), 0.98 (t, $J$ = 7.2 Hz, 6H), 0.87-0.79 (m, 14H) ppm; $^{13}C$ NMR (100 MHz, $CDCl_3$) $\delta$: 137.0, 159.2, 159.0, 158.0, 147.7, 145.8, 135.5, 133.6, 133.1, 132.9, 132.4, 131.7, 130.8, 130.0, 129.6, 128.2, 127.8, 127.7, 125.6, 124.7, 122.0, 120.0, 119.2, 117.2, 114.9, 113.6, 107.7, 105.2, 68.2, 55.4, 32.4, 31.9, 31.8, 29.9, 29.8, 29.3, 29.2, 29.1, 28.7, 27.6, 25.9, 22.7, 22.6, 22.5, 14.11 (2C), 14.07 ppm; HR-MS (MALDI: matrix dithranol) (*m*/*z*): $[M+Na]^+$ calcd for $C_{98}H_{112}N_6O_4$+Na: 1459.8637, found 1459.8652.

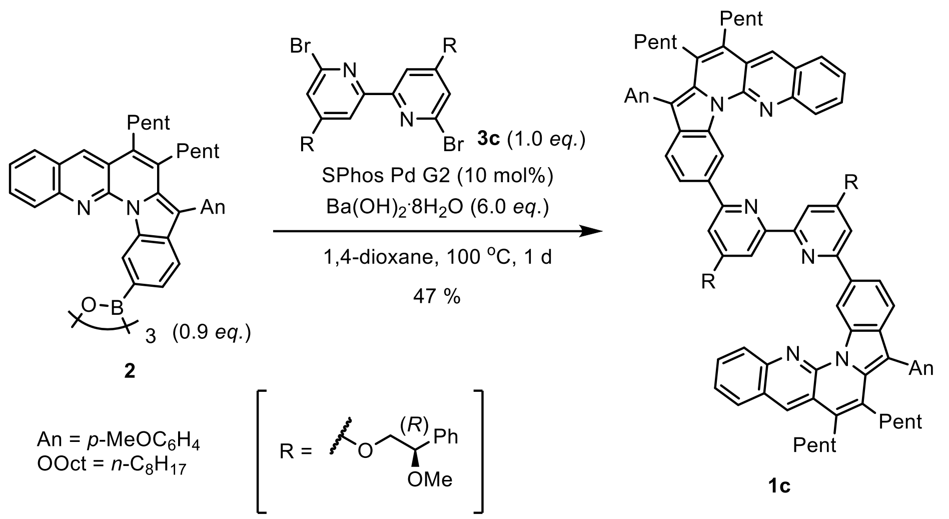


**2,2'-{4,4'-bis((*R*)-2-methoxy-2-phenylethoxy)-(2,2'-bipyridyl)-6,6'-diyl}bis({5-(4-methoxyphenyl)-6,7-dipentyldibenzopyrrolo[1,2-*a*][1,8]naphthyridine}) 1c**: To a mixture of boroxine **2**[S1] (30 mg, 18 μmol) and 6,6'-dibromo-4,4'-dioctoxy-2,2'-bipyridine **3c** (12 mg, 20 μmol) in 1,4-dioxane (0.5 mL) was added SPhos Pd G2 (1.4 mg, 2 μmol) and $Ba(OH)_2 \cdot 8H_2O$ (38 mg, 0.12 mmol). The mixture was heated at 100 ˚C for 1 d. After the reaction mixture was cooled to room temperature, it was added $CHCl_3$ and water. The aqueous phase was extracted with $CHCl_3$ and the combined organic phases were washed with water and brine. The organic phase was dried over $MgSO_4$ and concentrated *in vacuo.* The residue was purified by silica gel column chromatography ($CHCl_3$ to $CHCl_3$/TEA=100/1) and obtained solid was purified by GPC to give **1c** (14 mg, 47%) as an orange solid:

Mp 124.7-127.3 °C; IR (ATR): 3059, 3031, 2952, 2927, 2868, 1578, 1557, 1541, 1485, 1451, 1399, 1308, 1282, 1243, 1173, 1117, 1063, 1035, 833, 794, 700 $cm^{-1}$; $^1H$ NMR (400 MHz, $CDCl_3$) $\delta$: 10.67 (d, $J$ = 0.9 Hz, 2H), 8.43 (d, $J$ = 1.4 Hz, 2H), 8.36 (s, 2H), 8.24 (d, $J$ = 8.4 Hz, 2H), 8.21 (d, $J$ = 8.4 Hz, 2H), 7.93 (d, $J$ = 8.0 Hz, 2H), 7.74 (td, $J$ = 8.3, 1.2 Hz, 2H), 7.66 (d, $J$ = 1.9 Hz, 2H), 7.52 (t, $J$ = 7.4 Hz, 2H), 7.48 (d, $J$ = 8.0 Hz, 4H), 7.44 (d, $J$ = 8.3 Hz, 2H), 7.32-7.16 (m, 10H), 7.07 (d, $J$ = 8.6 Hz, 4H), 4.64 (dd, $J$ = 7.9, 3.4, 2H), 4.44 (dd, $J$ = 10.4, 7.9, 2H), 4.34 (dd, $J$ =10.4, 3.4, 2H), 3.95 (s, 6H), 3.31 (s, 6H), 2.92 (t, $J$ = 7.9 Hz, 4H), 2.62 (t, $J$ = 8.4 Hz, 4H), 1.74 (qn, $J$ = 7.9 Hz, 4H), 1.62-1.51 (m, 4H), 1.47 (sx, $J$ = 7.6 Hz, 4H), 1.42-1.32 (m, 4H) , 1.14 (sx, $J$ = 7.3 Hz, 4H), 1.15 (sx, $J$ = 7.3 Hz, 4H), 0.99 (t, $J$ = 7.2 Hz, 6H), 0.88-0.76 (m, 10H) ppm; $^{13}C$ NMR (100 MHz, $CDCl_3$) $\delta$: 166.6, 159.3, 159.0, 157.9, 147.7, 145.8, 138.1, 135.3, 133.6, 133.1, 132.9, 132.4, 131.7, 130.8, 130.0, 129.7, 128.5, 128.2, 128.1, 127.9, 127.7, 126.9, 125.6, 124.8, 122.1, 120.0, 119.3, 117.2, 114.9, 113.6, 108.1, 105.0, 81.9, 72.1, 57.2, 55.5, 32.4, 31.9, 29.9, 29.8, 28.7, 27.6, 22.6, 22.5, 14.11, 14.08 ppm; HR-MS (MALDI: matrix DCTB) (*m*/*z*): $[M]^+$ calcd for $C_{100}H_{100}N_6O_2$: 1480.7699, found 1480.7711; $[\alpha]_D^{23}$=-53.3 ($c$=0.138, $CH_2Cl_2$).

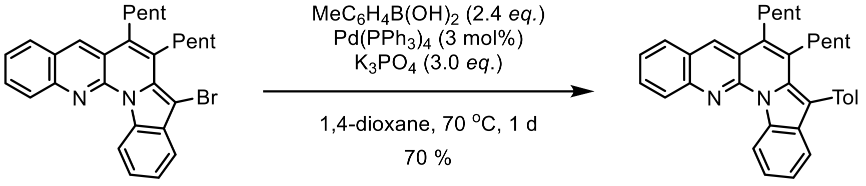


**6** Tol = *p*-$MeC_6H_4$ **7**

**5-(4-methylphenyl)-6,7-dipentyldibenzopyrrolo[1,2-*a*][1,8]naphthyridine 7:** To a mixture of 5-bromo-6,7-dipentyldibenzopyrrolo[1,2-*a*][1,8]naphthyridine **6**[S2] (243 mg, 0.50 mmol), $MeC_6H_4B(OH)_2$ (136 mg, 1.2 mmol) and $K_3PO_4$ (318 mg, 1.5 mmol) in 1,4-dioxane (2.5 mL) was added $Pd(PPh_3)_4$ (17 mg, 15 μmol). The mixture was heated at 70 ˚C for 1 d. After the reaction mixture was cooled to room temperature, it was diluted $CHCl_3$ and the solution was washed with $H_2O$ and brine. The organic phase was dried over $MgSO_4$ and concentrated *in vacuo.* The residue was purified by silica gel column chromatography (hexane/$CHCl_3$ = 10:1) to give **7** (174 mg, 70%) as a yellow solid:

Mp 111.2-111.8 °C; IR (ATR): 3057, 2952, 2927, 2866, 1587, 1545, 1456, 1392, 1348, 1302, 1261, 1228, 1211, 1180, 1157, 1140, 1107, 1020, 901, 787, 744 $cm^{-1}$; $^1H$ NMR (400 MHz, $CDCl_3$) $\delta$: 9.82 (dt, *J* = 8.4, 0.8 Hz, 1H), 8.31 (s, 1H), 8.20 (d, *J* = 8.4 Hz, 1H), 7.89 (dd, *J* = 8.2, 1.1 Hz, 1H), 7.72 (td, *J* = 6.9, 1.5 Hz, 1H), 7.51 (td, *J* = 5.9, 2.5 Hz, 1H), 7.48 (td, *J* = 6.9, 1.2 Hz, 1H), 7.38 (d, *J* = 8.0 Hz, 2H), 7.35-7.30 (m, 2H), 7.28 (d, *J* = 7.7 Hz, 2H), 2.87 (t, *J* = 8.1 Hz, 2H), 2.55 (t, *J* = 8.4 Hz, 2H), 2.47 (s, 3H), 1.69 (qn, *J* = 8.3 Hz, 2H), 1.59-1.48 (m, 2H), 1.43 (sx, *J* = 7.1 Hz, 2H), 1.36-1.24 (m, 2H), 1.09 (sx, *J* = 7.4 Hz, 2H), 0.96 (t, *J* = 7.2 Hz, 3H), 0.78 (t, *J* = 7.2 Hz, 3H), 0.73 (sx, *J* = 7.4 Hz, 2H) ppm; $^{13}C$ NMR (100 MHz, $CDCl_3$) $\delta$: 147.7, 154.7, 136.7, 133.2, 133.0, 132.1, 131.8, 131.3, 130.7, 129.5, 129.4, 128.7, 127.7, 127.6, 125.5, 124.6, 123.6, 122.6, 120.0, 119.3, 118.0, 115.2, 32.4, 31.8, 29.8, 29.7, 28.7, 27.6, 22.5, 22.4, 21.3, 14.09, 14.07 ppm; HR-MS (MALDI: matrix dithranol) (*m*/*z*): $[M]^+$ calcd for $C_{36}H_{38}N_2$: 498.3030, found 498.3014

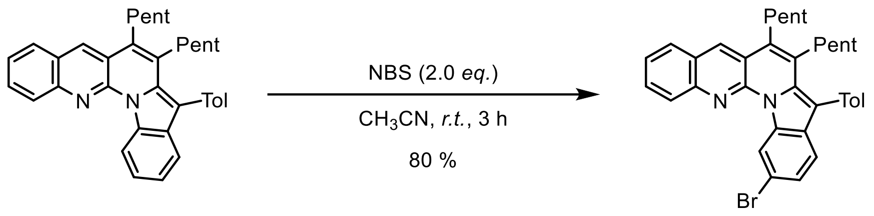


**7** Tol = *p*-MeC$_6$H$_4$ **8**

**2-Bromo-5-(4-methylphenyl)-6,7-dipentyldibenzopyrrolo[1,2-*a*][1,8]naphthyridine 8:** To a mixture of 5-(4-methylphenyl)-6,7-dipentyldibenzopyrrolo[1,2-*a*][1,8]naphthyridine **7** (150 mg, 0.30 mmol) in MeCN (3 mL) was added NBS (108 mg, 0.60 mmol) in MeCN (2 mL) at 0 °C. The reaction mixture was warmed to room temperature, and stired for 3 h. After the reaction, it was quenched by 10% $NaHSO_3$ *aq*. The aqueous phase was extracted with $CHCl_3$ and the combined organic phase were washed with $H_2O$ and brine. The organic phase was dried over $MgSO_4$ and concentrated *in vacuo*. The residue was purified by silica gel column chromatography (hexane/$CHCl_3$ = 9:1 to 4:1) to give **8** (138 mg, 80%) as a yellow solid:

Mp 166.6-167.1 °C; IR (ATR): 3114, 3051, 3022, 2956, 2924, 2856, 1622, 1591, 1562, 1541, 1493, 1469, 1452, 1392, 1331, 1282, 1228, 1178, 1159, 1120, 1024, 935, 874, 800, 739, 590, 505 cm$^{-1}$; $^{1}$H NMR (400 MHz, $CDCl_3$) *δ*: 10.03 (d, *J* = 1.7 Hz, 1H), 8.34 (s, 1H), 8.24 (d, *J* = 8.4 Hz, 1H), 7.90 (dd, *J* = 8.2, 1.0 Hz, 1H), 7.75 (td, *J* = 7.0, 1.5 Hz, 1H), 7.51 (td, *J* = 6.9, 1.1 Hz, 1H), 7.41 (dd, *J* = 8.4, 1.8 Hz, 1H), 7.35 (d, *J* = 8.0 Hz, 2H), 7.28 (d, *J* = 7.8 Hz, 2H), 7.17 (d, *J* = 8.4 Hz, 1H), 2.86 (t, *J* = 8.1 Hz, 2H), 2.54 (t, *J* = 8.5 Hz, 2H), 2.47 (s, 3H), 1.68 (qn, *J* = 8.4 Hz, 2H), 1.58-1.48 (m, 2H), 1.44 (sx, *J* = 7.6 Hz, 2H), 1.35-1.24 (m, 2H), 1.08 (sx, *J* = 7.4 Hz, 2H), 0.96 (t, *J* = 7.2 Hz, 3H), 0.78 (t, *J* = 7.2 Hz, 3H), 0.72 (sx, *J* = 7.4 Hz, 2H) ppm; $^{13}$C NMR (100 MHz, $CDCl_3$) *δ*: 147.3, 145.6, 137.0, 133.5, 132.4, 132.2, 131.4, 131.2, 131.1, 130.9, 130.0, 129.7, 128.8, 127.8, 127.7, 125.8, 125.6, 125.0, 120.8, 120.4, 119.8, 116.9, 114.9, 32.4, 31.8, 29.8, 29.7, 28.6, 27.6, 22.5, 22.4, 21.3, 14.08, 14.06 ppm; HR-MS (MALDI: matrix dithranol) (*m*/*z*): [M]$^+$ calcd for $C_{36}H_{37}BrN_2$: 576.2135, found 576.2125.

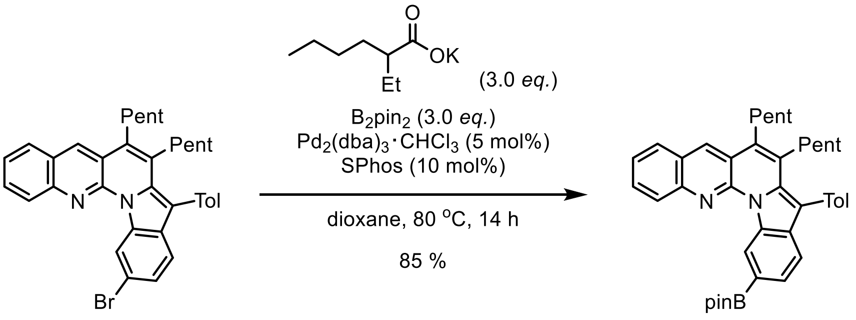


**8** Tol = *p*-$MeC_6H_4$ **9**

**2-(4,4,5,5-tetramethyl-1,3,2-dioxaborolan-2-yl)-5-(4-methylphenyl)-6,7-dipentyldibenzopyrrolo[1,2-*a*][1,8] naphthyridine 9:** To a mixture of 2-bromo-5-(4-methylphenyl)-6,7-dipentyldibenzopyrrolo[1,2-*a*][1,8] naphthyridine **8** (116 mg, 0.20 mmol), potassium 2-ethylhexanoate[S3] (110 mg, 0.60 mmol), $B_2pin_2$ (152 mg, 0.60 mmol), $Pd_2(dba)_3$· $CHCl_3$ (10 mg, 10 μmol) and SPhos (8.2 mg, 20 μmol) was added 1,4-dioxane (2 mL) . The mixture was heated at 80 ˚C for 14 h. After the reaction mixture was cooled to room temperature, it was diluted $CHCl_3$ and the solution was washed with $H_2O$ and brine. The organic phase was dried over $MgSO_4$ and concentrated *in vacuo.* The residue was dissolved into $CHCl_3$ and filtered through a small pad of silica gel, then the crude product was washed by MeOH to give **9** (108 mg, 85%) as an orange solid:

Mp 193.6-194.7 °C; IR (ATR): 3057, 3028, 2954, 2929, 2868, 1606, 1579, 1556, 1541, 1485, 1446, 1421, 1392, 1350, 1298, 1273, 1230, 1213, 1146, 1119, 1070, 968, 858, 748, 687, $cm^{-1}$; $^1H$ NMR (400 MHz, $CDCl_3$) $\delta$: 10.24 (s, 1H), 8.34 (s, 1H), 8.27 (d, $J$ = 8.4 Hz, 1H), 7.90 (dd, $J$ = 8.2, 1.1 Hz, 1H), 7.76 (td, $J$ = 6.9, 1.4 Hz, 1H), 7.73 (dd, $J$ = 7.9, 0.9 Hz, 1H), 7.50 (td, $J$ = 6.6, 1.2 Hz, 1H), 7.37 (d, $J$ = 8.0 Hz, 2H), 7.32-7.30 (m, 1H), 7.28 (d, $J$ = 7.9 Hz, 2H), 2.88 (t, $J$ = 8.1 Hz, 2H), 2.55 (t, $J$ = 8.6 Hz, 2H), 2.47 (s, 3H), 1.70 (qn, $J$ = 8.0 Hz, 2H), 1.60-1.48 (m, 2H), 1.49-1.36 (m, 2H), 1.44 (s, 12H), 1.36-1.25 (m, 2H), 1.09 (sx, $J$ = 7.4 Hz, 2H), 0.96 (t, $J$ = 7.2 Hz, 3H), 0.78 (t, $J$ = 7.2 Hz, 3H), 0.72 (sx, $J$ = 7.1 Hz, 2H) ppm; $^{13}C$ NMR (100 MHz, $CDCl_3$) $\delta$: 147.5, 145.8, 136.8, 134.4, 133.0, 133.0, 132.9, 131.6, 131.3, 130.9, 130.2, 129.4, 128.7, 128.4, 128.0, 127.6, 125.5, 124.9, 124.7, 119.8, 118.6, 115.3, 83.5, 32.4, 31.8, 29.8, 28.7, 27.6, 25.0, 22.5, 22.4, 21.3, 14.09, 14.07 ppm; HR-MS (MALDI: matrix dithranol) (*m*/*z*): $[M]^+$ calcd for $C_{42}H_{49}BN_2O_2$: 623.3889, found 623.3900.

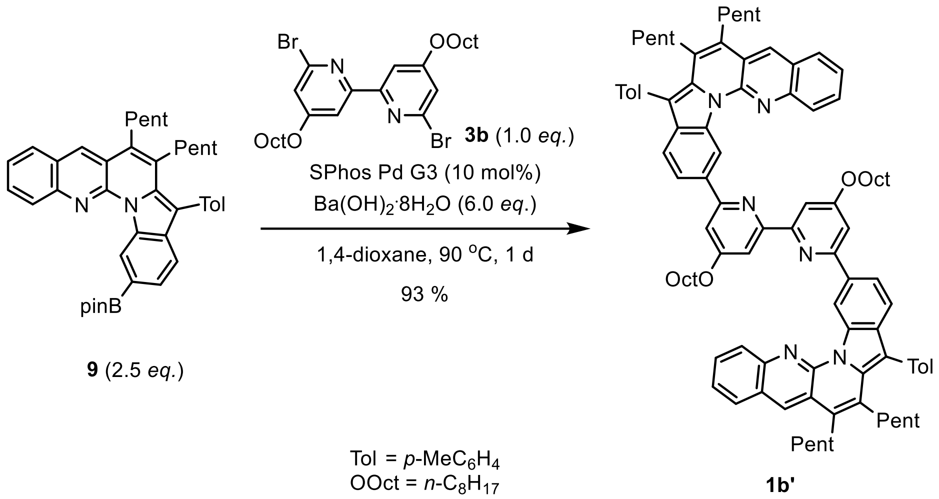


**2,2'-{4,4'-dioctoxy(2,2'-bipyridyl)-6,6'-diyl}bis({5-(4-methylphenyl)-6,7-dipentyldibenzopyrrolo[1,2-*a*][1,8] naphthyridine}) 1b':** To a mixture of boronic acid pinacol ester **9** (22 mg, 3.5 μmol) and 6,6'-dibromo-4,4'-dioctoxy-2,2'-bipyridine **3b** (8.0 mg, 14 μmol), SPhos Pd G3 (1.1 mg, 1.4 μmol) and $Ba(OH)_2\cdot 8H_2O$ (26 mg, 84 μmol) was added 1,4-dioxane (0.1 mL). The mixture was heated at 90 ˚C for 1 d. After the reaction mixture was cooled to room temperature, it was diluted $CHCl_3$ and the solution was washed with $H_2O$ and brine. The organic phase was dried over $MgSO_4$ and concentrated *in vacuo.* The residue was purified by silica gel column chromatography ($CHCl_3$) to give **1b'** (18 mg, 93%) as a yellow solid:

Mp 87.1-102.0 (decomp.); IR (ATR): 3168, 3128, 3041, 2951, 2924, 2852, 1577, 1556, 1481, 1450, 1392, 1308, 1281, 1228, 1211, 1155, 1107, 822, 744 $cm^{-1}$; $^1H$ NMR (400 MHz, $CDCl_3$) $\delta$: 10.72 (d, $J$ = 1.1 Hz, 2H), 8.43 (d, $J$ = 2.2, 2H), 8.36 (s, 2H), 8.29 (d, $J$ = 8.4 Hz, 2H), 8.19 (dd, $J$ = 8.4, 1.5 Hz, 2H), 7.93 (d, $J$ = 8.2 Hz, 2H), 7.74 (td, $J$ = 6.9, 1.4 Hz, 2H), 7.60 (d, $J$ = 2.2 Hz, 2H), 7.51 (td, $J$ = 7.1, 0.9 Hz, 2H), 7.46-7.41 (m, 6H), 7.32 (d, $J$ = 7.8 Hz, 4H), 4.25 (t, $J$ = 6.7 Hz, 4H), 2.91 (t, $J$ = 8.1 Hz, 4H), 2.59 (t, $J$ = 8.1 Hz, 4H), 2.50 (s, 6H), 1.80 (qn, $J$ = 7.0 Hz, 4H), 1.76-1.66 (m, 4H), 1.62-1.51 (m, 4H), 1.46 (sx, $J$ = 7.8 Hz, 4H), 1.41-1.16 (m, 24H), 1.11 (sx, $J$ = 7.4 Hz, 4H), 0.98 (t, $J$ = 7.2 Hz, 6H), 0.85 (t, $J$ = 6.7 Hz, 6H), 0.80 (t, $J$ = 7.2 Hz, 6H), 0.76 (sx, $J$ = 7.5 Hz, 4H) ppm; $^{13}C$ NMR (100 MHz, $CDCl_3$) $\delta$: 167.0, 159.2, 158.0, 147.7, 145.8, 136.8, 135.5, 133.7, 133.0, 133.0, 132.7, 131.7, 131.3, 130.8, 129.9, 129.5, 128.7, 127.8, 127.7, 125.6, 124.7, 122.0, 120.0, 119.2, 117.2, 115.2, 107.6, 105.2, 68.2, 32.4, 31.84, 31.78, 29.84, 29.79, 29.3, 29.24, 29.15, 28.7, 27.6, 25.9, 22.7, 22.6, 22.4, 21.3, 14.1 (3C) ppm; HR-MS (MALDI: matrix dithranol) (*m*/*z*): $[M+H]^+$ calcd for $C_{98}H_{112}N_6O_2$+H: 1405.8920, found 1405.8924.

## 3. Complexation of strands with a Zn(II) cation

**Complexation to [(1b)$_2$Zn][OTf]$_2$**

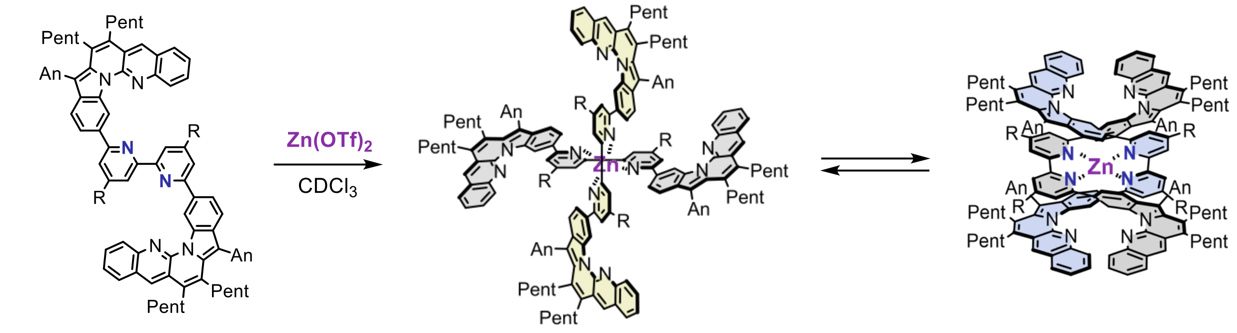


**1b**
(R = OOct, An = *p*-MeOC$_6$H$_4$)

[(**1b**)$_2$Zn][OTf]$_2$
open form

[(**1b**)$_2$Zn][OTf]$_2$
double-helical form

**Zinc complex [(1b)$_2$Zn][OTf]$_2$ :** To an NMR tube was charged with **1b** (1.4 mg, 1 µmol) and $CDCl_3$ (0.50 mL) was added $Zn(OTf)_2$ in acetone-$d_6$ (50 mM, 10 µL, 0.5 µmol, 0.5 *eq.*) and MS3A (10 mg). After the NMR tube was stirred for 1 min, the $^1$H NMR and ROESY spectra of the resulting mixture were recorded (Figure S1, Figure S2), which confirmed the formation of [(**1b**)$_2$Zn][OTf]$_2$.

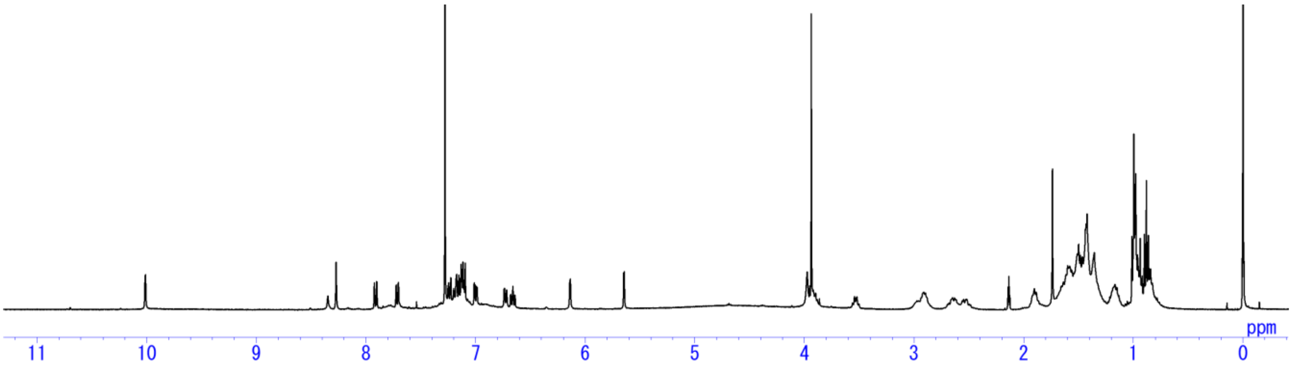


Figure S1. $^1$H NMR of [(**1b**)$_2$Zn][OTf]$_2$ ($CDCl_3$, 400 MHz, 298 K).

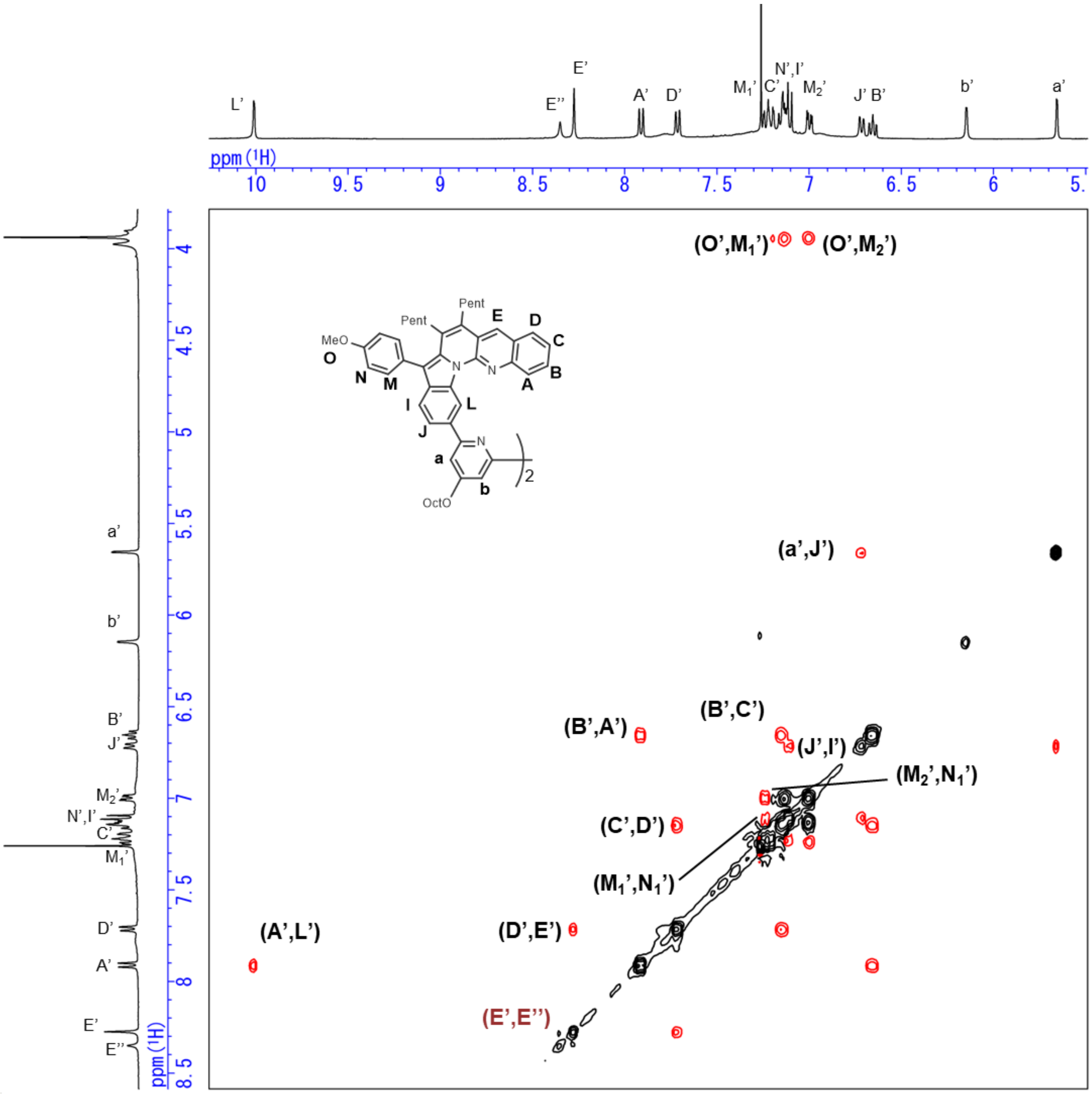


Figure S2. 2D ROESY of [(**1b**)$_2$Zn][OTf]$_2$ ($CDCl_3$, 298 K, mixing time = 1.0 s).

**Complexation to [(*R*)-(1c)$_2$Zn][OTf]$_2$**

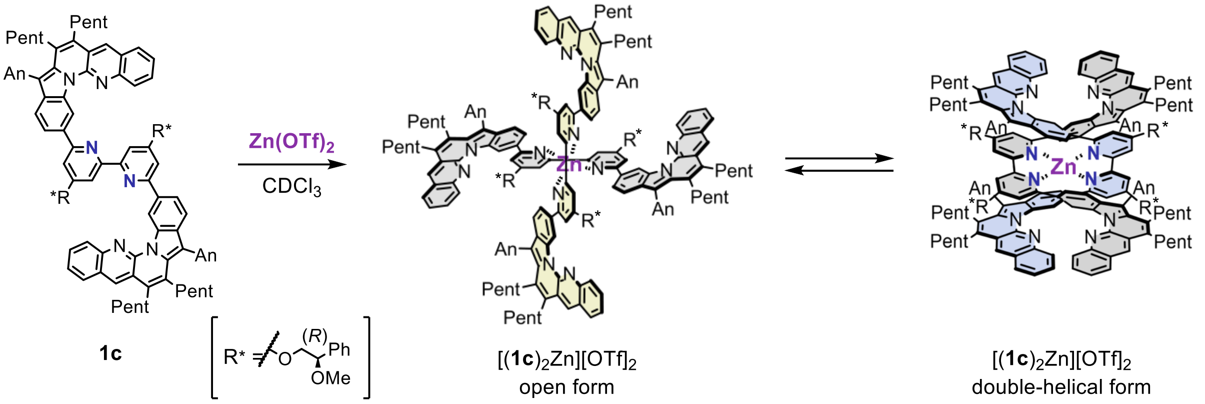


**Zinc complex [(*R*)-(1c)$_2$Zn][OTf]$_2$ :** To an NMR tube was charged with **1c** (1.5 mg, 1 μmol) and $CDCl_3$ (0.50 mL) was added $Zn(OTf)_2$ in acetone-$d_6$ (50 mM, 10 μL, 0.5 μmol, 0.5 *eq.*) and MS3A (10 mg). After the NMR tube was stirred for 1 min, the $^1$H NMR spectrum of the resulting mixture was recorded (Figure S3), which confirmed the formation of [(*R*)-(**1c**)$_2$Zn][OTf]$_2$.

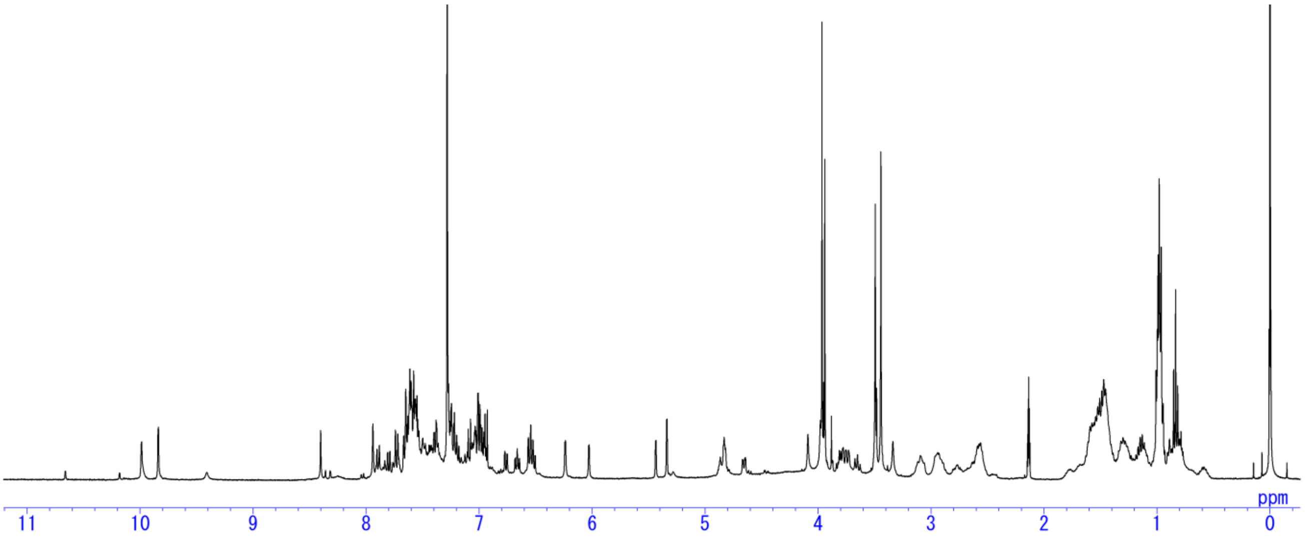


Figure S3. $^1$H NMR of [(*R*)-(**1c**)$_2$Zn][OTf]$_2$ ($CDCl_3$, 400 MHz, 298 K).

**Complexation to [(1b’)$_2$Zn][OTf]$_2$**

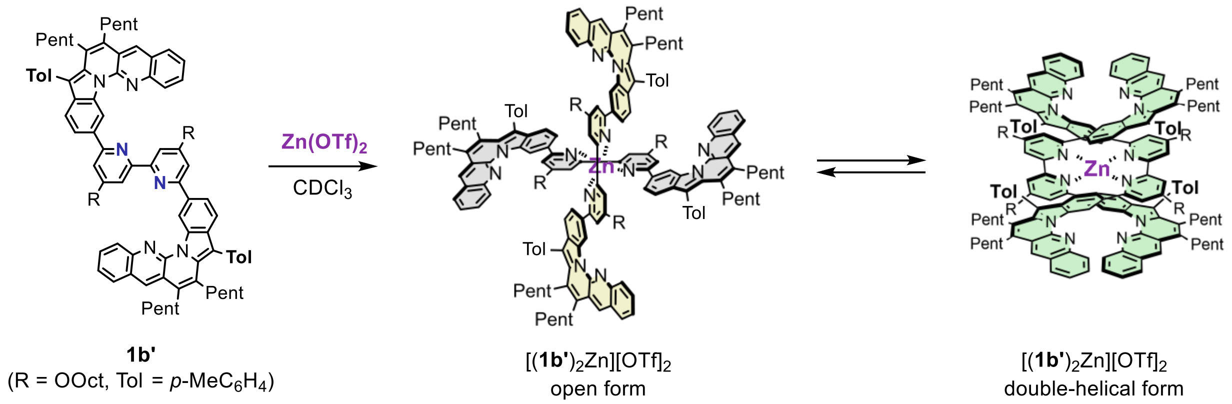


**Zinc complex [(1b’)$_2$Zn][OTf]$_2$ :** To an NMR tube was charged with **1b’** (1.4 mg, 1 µmol) and $CDCl_3$ (0.50 mL) was added $Zn(OTf)_2$ in acetone-$d_6$ (50 mM, 10 µL, 0.5 µmol, 0.5 *eq.*) and MS3A (10 mg). After the NMR tube was stirred for 1 min, the $^1H$ NMR spectrum of the resulting mixture was recorded (Figure S4), which confirmed the formation of [(**1b’**)$_2$Zn][OTf]$_2$.

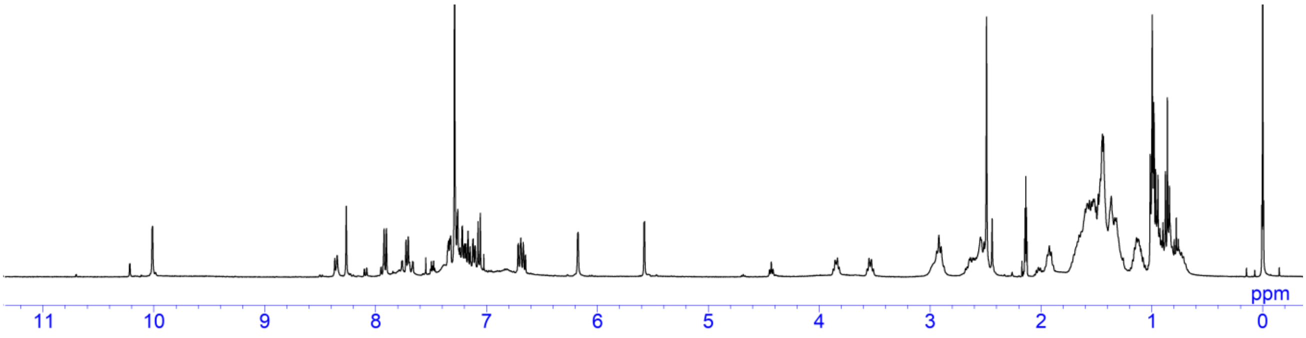


Figure S4. $^1H$ NMR of [(**1b’**)$_2$Zn][OTf]$_2$ ($CDCl_3$, 400 MHz, 298 K).

## 4. Single crystal X-ray structural analysis

**General**. Suitable crystals for diffraction experiments were obtained by diffusion method from a $C_6H_5Br$/hexane mixture for **1a** and [(**1a**)$_2$Zn][OTf]$_2$ and from a $C_6H_5Br$/acetone/$Et_2O$ mixture for complex [(**1a**)$_2$Ag][$PF_6$]. The single X-ray structure determination was performed on Rigaku XtaLAB Synergy-DW diffractometer (CuKα radiation, λ = 1.54184 Å). A numerical absorption correction (μ) was applied. The structure was solved by direct methods in Olex2 and refined by the full-matrix least-squares method on $F^2$ with anisotropic temperature factors for non-hydrogen atoms.[S4-6] All the hydrogen atoms were located at the calculated positions and refined with riding. One of the disordered alkyl chains of [(**1a**)$_2$Zn][OTf]$_2$ was restricted by DFIX, DANG, SIMU and RIGU. One of the disordered alkyl chains of [(**1a**)$_2$Ag][$PF_6$] was restricted by DFIX, DANG, SIMU and RIGU. The electron density related to disordered solvent molecules and counter-ions, which could not be represented through discrete atomic positions, was addressed through the implementation of the Solvent Mask routine in Olex2. Crystallographic data collection and refinement information is listed in Table S1.The final cif files were checked by IUCR's checkcif.

Table S1. Crystal data and structure refinement parameters for **1a**, [(**1a**)$_2$Zn][OTf]$_2$ and [(**1a**)$_2$Ag][PF$_6$]

| **Compound** | | **1a** | [(**1a**)$_2$Zn][OTf]$_2$ | [(**1a**)$_2$Ag][PF$_6$] |
|---|---|---|---|---|
| Data deposition | | 2354002 | 2354004 | 2354003 |
| Empirical formula | | $C_{82}H_{80}N_6O_2$ | $C_{164}H_{160}N_{12}O_4Zn$ (Solvent Mask) | $C_{164}H_{160}N_{12}O_4Ag$, $PF_6$, $C_4H_{10}O$ |
| Formula weight | | 1181.52 | 2428.40 | 2689.99 |
| Crystal habit | | yellow needle crystal | red block crystal | yellow plate crystal |
| Crystal size | | 0.3×0.15×0.1 mm$^3$ | 0.1×0.1×0.1 mm$^3$ | 0.3×0.2×0.05 mm$^3$ |
| Temperature | | 93 K | 93 K | 93 K |
| Wavelength | | 1.54184 Å | 1.54184 Å | 1.54184 Å |
| Crystal system | | triclinic | monoclinic | monoclinic |
| Space group | | *P*-1 | $P2_1/c$ | $P2_1/c$ |
| Unit cell dimensions | $a$ | 11.8968(4) Å | 22.1953(7) Å | 29.3564(4) Å |
| | $b$ | 12.1495(5) Å | 22.8757(7) Å | 16.47772(18) Å |
| | $c$ | 13.2542(5) Å | 30.6110(7) Å | 29.4648(3) Å |
| | $\alpha$ | 64.227(4) ° | 90 ° | 90 ° |
| | $\beta$ | 69.450(4) ° | 88.224(6) ° | 90.4168(12) ° |
| | $\gamma$ | 67.726(4) ° | 90.533(2) ° | 90 ° |
| Volume | | 1556.09(13) Å$^3$ | 15541.5(8) Å$^3$ | 14252.5(3) Å$^3$ |
| *Z* | | 1 | 4 | 4 |
| Density (calculated) | | 1.261 g/cm$^3$ | 1.038 g/cm$^3$ | 1.254 g/cm$^3$ |
| Absorption coefficient Mu | | 0.583 mm$^{-1}$ | 0.629 mm$^{-1}$ | 1.793 mm$^{-1}$ |
| *F*(000) | | 630 | 5160 | 5672 |
| Theta range for data collection | | 3.799 ° to 71.364 ° | 2.411 ° to 76.457 ° | 3.000 ° to76.687 ° |
| Index ranges | | $-14<=h<=14$ | $-27<=h<=27$ | $-34<=h<=36$ |
| | | $-14<=k<=7$ | $-28<=k<=24$ | $-17<=k<=20$ |
| | | $-16<=l<=16$ | $-37<=l<=36$ | $-36<=l<=37$ |
| Reflections collected | | 14863 | 116304 | 107706 |
| Refinement method | | Full-matrix least-squares on $F^2$ | Full-matrix least-squares on $F^2$ | Full-matrix least-squares on $F^2$ |
| Data/restraints/parameters | | 5859 /0/409 | 31305 /142 /1713 | 28817 /179 /1818 |
| Goodness-of-fit on $F^2$ | | 1.059 | 1.051 | 1.096 |
| Final *R* indices | | $R_1$ = 0.0437 [$I$>2σ($I$)] , $wR_2$ = 0.1220 (all data) | $R_1$ = 0.0866 [$I$>2σ($I$)] , $wR_2$ = 0.2759 (all data) | $R_1$ = 0.1205 [$I$>2σ($I$)] , $wR_2$ = 0.3444 (all data) |

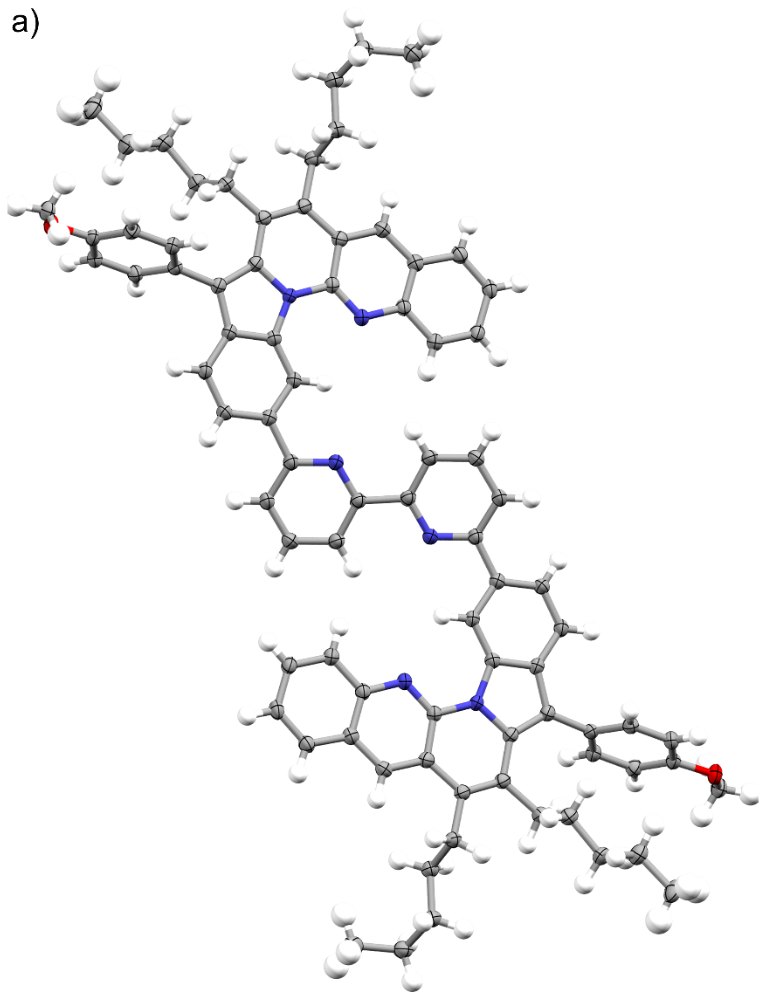


Figure S5. ORTEP drawings of X-ray structures of **1a** with thermal ellipsoids at 50% probability; a) Front view and b) Side view.

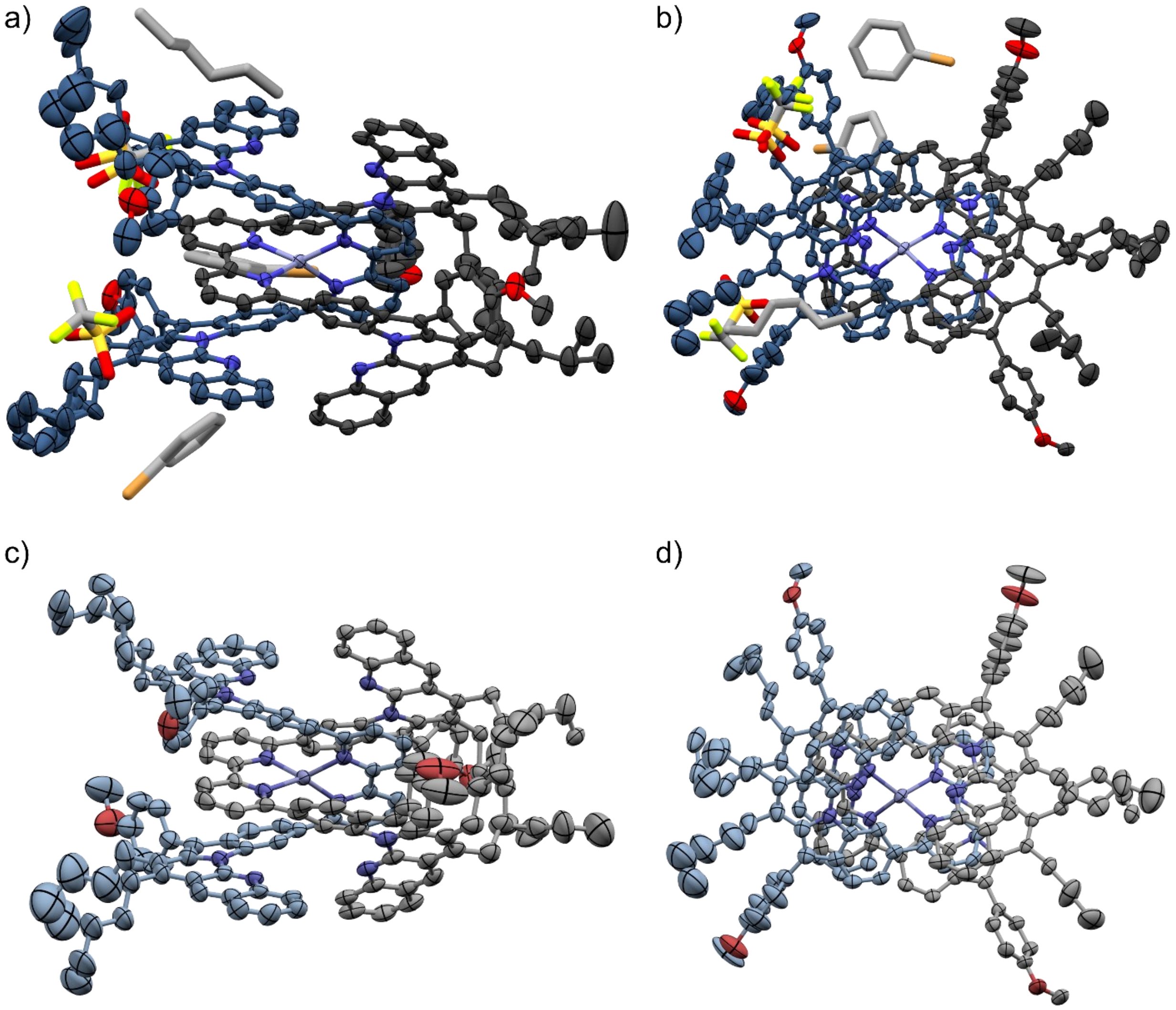


Figure S6. a) Front view and b) Top view of X-ray structures of [(**1a**)$_2$Zn][OTf]$_2$ with the disordered solvents and counter-anions being resolved through the application of isotropic refinement parameters. The solvents and counter-ions were not adequately refined. The crystal structure of [(**1a**)$_2$Zn][OTf]$_2$ including of solvents and counter-ions, were subjected to analyzed as preliminary examination to verify the placement and quantity of counter-ions ($R_1$ = 0.23, $wR_2$ = 0.58). c) Front view and d) Top view of X-ray structures of [(**1a**)$_2$Zn]$^{2+}$ (solvent mask).

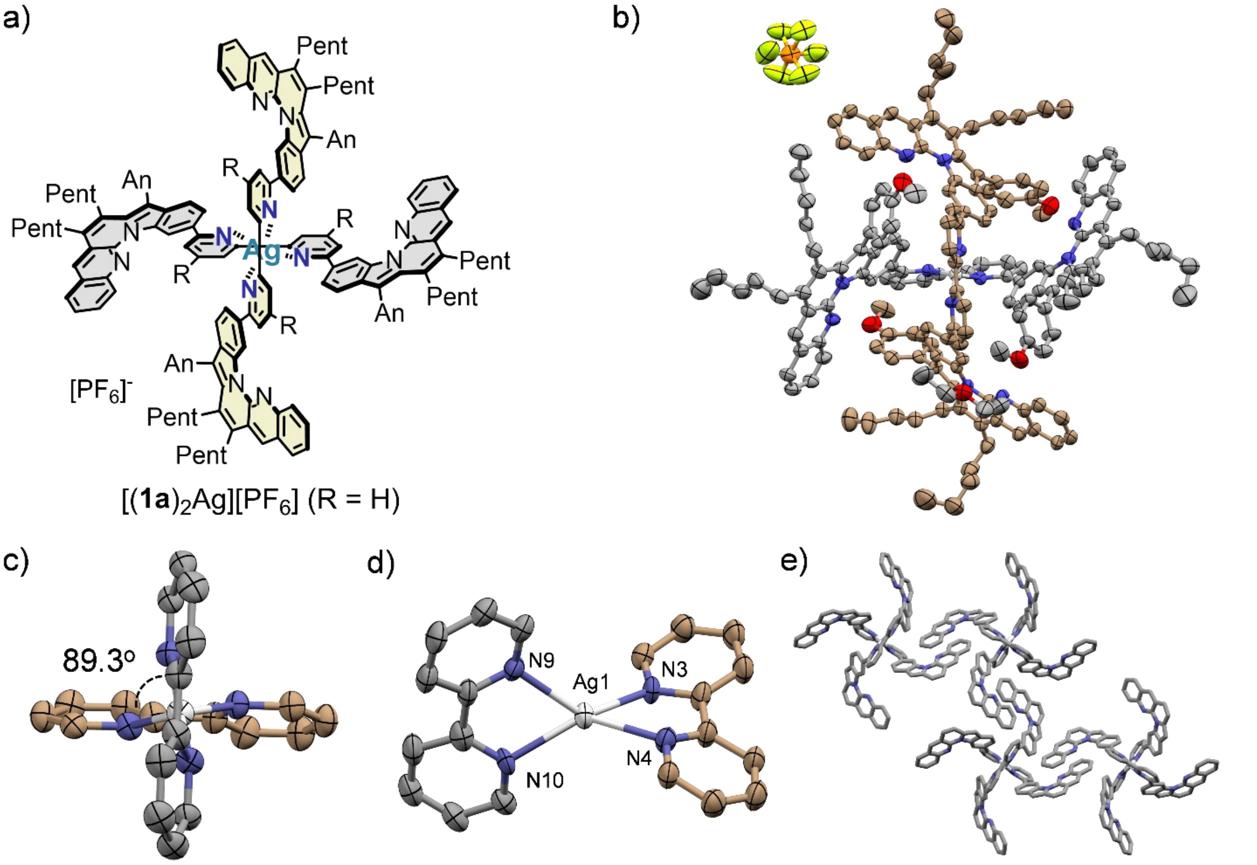


Figure S7. X-ray structures of [(**1a**)$_2$Ag][PF$_6$], a) the structural formula of [(**1a**)$_2$Ag][PF$_6$]; ORTEP drawings of X-ray structures of [(**1a**)$_2$Cu]$^{2+}$ with thermal ellipsoids at 50% probability; minor disordered parts and hydrogen atoms are omitted for clarity; b) Front view; c, d) Coordination geometry of [(**1a**)$_2$Ag][PF$_6$], Selected atom distances and angles: N3- Ag1 2.333(5) Å, N4-Ag1 2.377(5) Å, N9-Ag1 2.368(5) Å, N10-Ag1 2.347(5) Å, N3-Ag1-N4 70.6(2)°, N4-Ag1-N10 132.6(2)°, N9-Ag1-N10 70.2(2)°, N10-Ag1-N3 132.6(2)°; e) Crystal packing of complex [(**1a**)$_2$Ag][PF$_6$] (all side chains, solvents and counter anions are omitted for clarity).

## 5. Kinetic stability of monometallofoldamers and intercomplex equilibrium

Two experiments A and B were carried out to investigate the possibility of ligand exchange in the monometallofoldamers [(**1**)$_2$Zn][OTf]$_2$ (Scheme S1). In the experiment A, the kinetic mixture of [(**1b**)$_2$Zn][OTf]$_2$/[(**1b**)(**1b'**)Zn][OTf]$_2$/[(**1b'**)$_2$Zn][OTf]$_2$ (~ 1/2/1) was prepared kinetically by adding Zn(OTf)$_2$ to a mixed solution of a strand **1b** and an analogue **1b'** with tolyl groups instead of anisyl groups (Figure S8, Figure S9). The ratio of the complexes remained unchanged under the conditions of $CDCl_3$, 40 °C, 1 d, suggesting that the (i) the mixture used in this experiment had been already in equilibrium, or (ii) monometallofoldamers were not in intercomplex equilibrium (or were in very slow intercomplex equilibrium) (Scheme S2, Figure S10, Figure S11). As the experiment B to exclude the possibility of intercomplex equilibrium, the two complexes [(**1b**)$_2$Zn][OTf]$_2$ and its analogue [(**1b'**)$_2$Zn][OTf]$_2$ were mixed and heated at 40 ˚C in $CDCl_3$ for 1 d to study whether the heteroleptic complex [(**1b**)(**1b'**)Zn][OTf]$_2$ form (Scheme S3). The result of the experiment B where the heteroleptic complex did not form suggested that (ii) monometallofoldamers were not in intercomplex equilibrium (or were in very slow intercomplex equilibrium) when considered in conjunction with the experiment A.

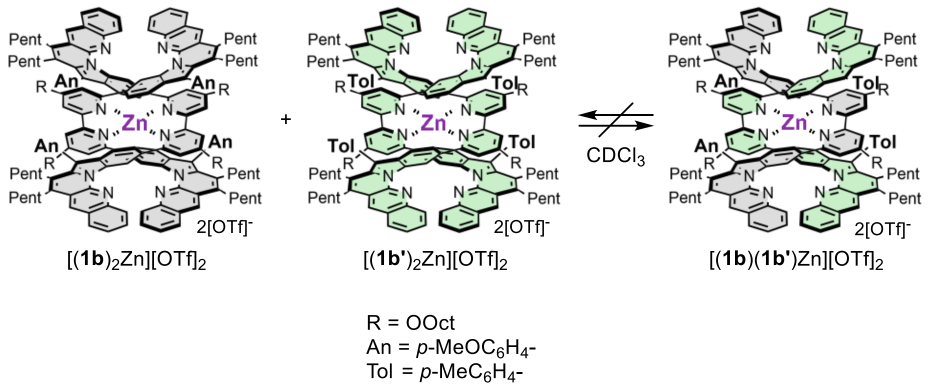


Scheme S1. The intercomplex equilibrium in the mixture of [(**1b**)$_2$Zn][OTf]$_2$, [(**1b**)(**1b'**)Zn][OTf]$_2$ and [(**1b'**)$_2$Zn][OTf]$_2$.

**The complexation of [(1b)(1b')Zn][OTf]$_2$**

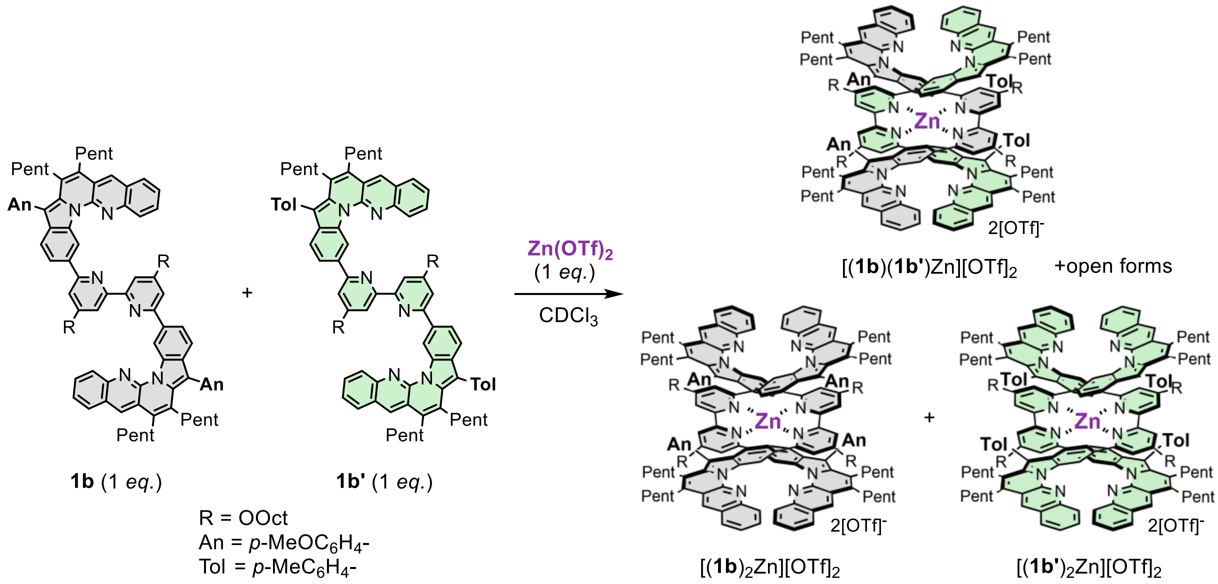


**Zinc complex [(1b)(1b')Zn][OTf]$_2$:** To an NMR test tube was charged with **1b** (0.7 mg, 0.5 μmol, 1 *eq.*) **1b'** (0.7 mg, 0.5 μmol, 1 *eq.*) and $CHCl_3$ (0.50 mL) was added $Zn(OTf)_2$ in acetone (50 mM, 10 μL, 0.5 μmol, 1 *eq.*) and MS3A (10 mg). After the NMR tube was stirred for 1 min, and the solution was concentrated to give a almostly statistical ratio of mixture of [(**1b**)$_2$Zn][OTf]$_2$/[(**1b**)(**1b'**)Zn][OTf]$_2$/[(**1b'**)$_2$Zn][OTf]$_2$ (~ 1/2/1) (Figure S8, Figure S9).

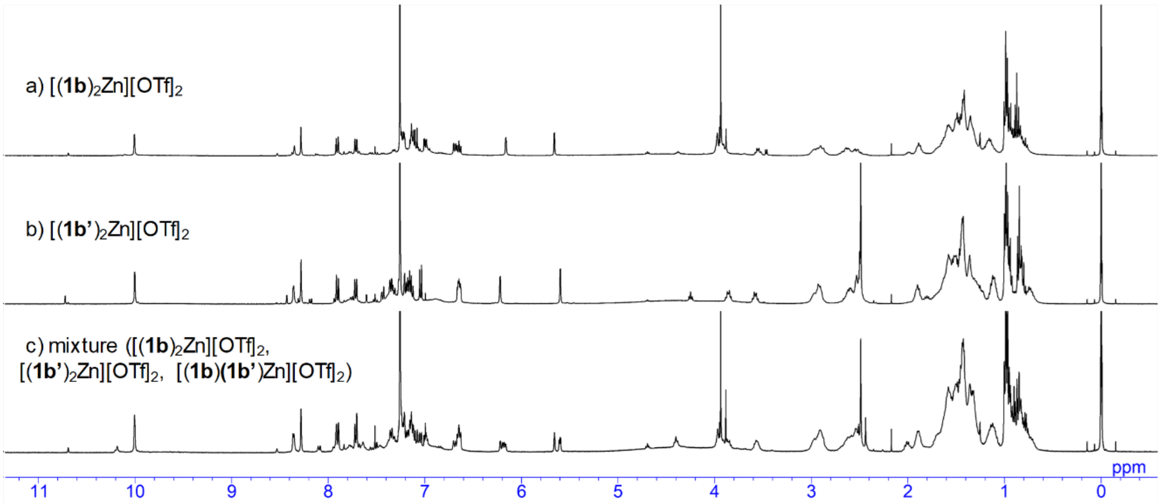


Figure S8. $^1$H NMR spectra of a) [(**1b**)$_2$Zn][OTf]$_2$, b) [(**1b'**)$_2$Zn][OTf]$_2$ and c) a mixture of [(**1b**)$_2$Zn][OTf]$_2$, [(**1b'**)$_2$Zn][OTf]$_2$ and [(**1b**)(**1b'**)Zn][OTf]$_2$ ($CDCl_3$, 400 MHz, 298 K).

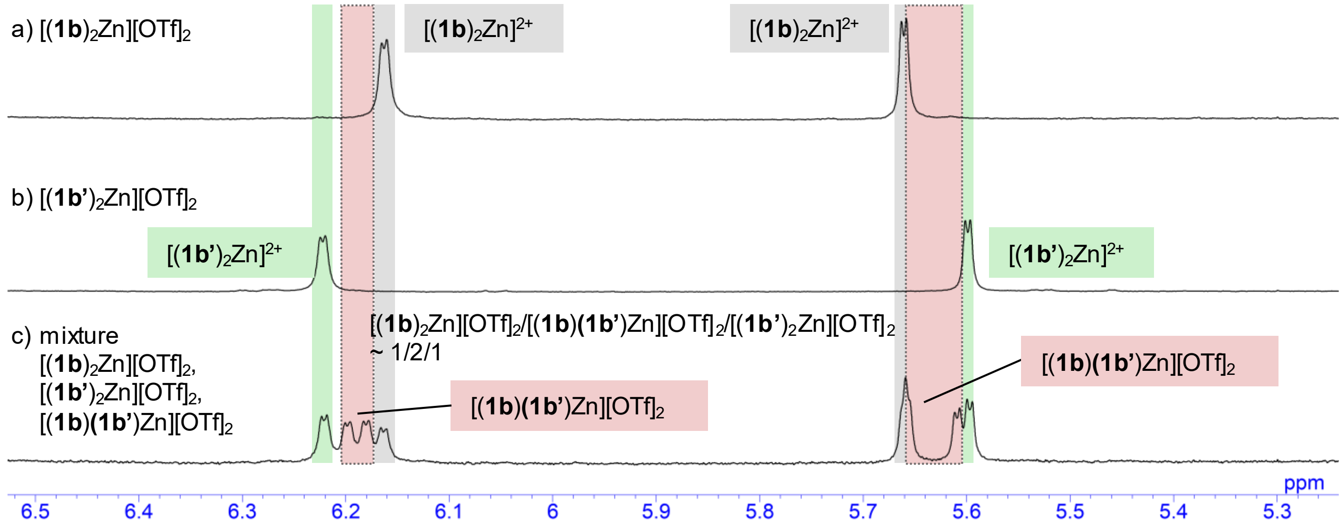


Figure S9. $^{1}$H NMR spectra (5.3-6.5 ppm) of a) [(**1b**)$_2$Zn][OTf]$_2$, b) [(**1b'**)$_2$Zn][OTf]$_2$ and c) a mixture of [(**1b**)$_2$Zn][OTf]$_2$, [(**1b'**)$_2$Zn][OTf]$_2$ and [(**1b**)(**1b'**)Zn][OTf]$_2$ ($CDCl_3$, 400 MHz, 298 K).

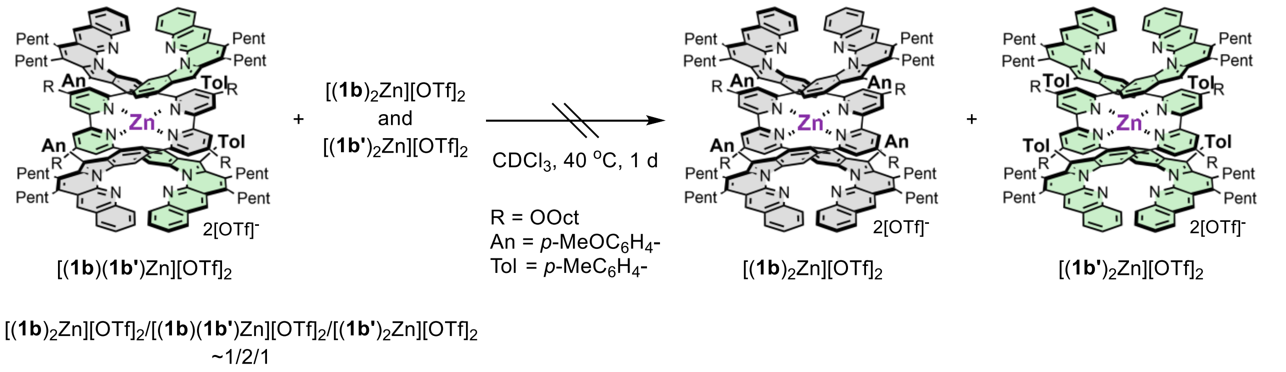


Scheme S2. **The experiments A**: An NMR tube was charged with a mixture of [(**1b**)$_2$Zn][OTf]$_2$ (~0.25 mM, 0.5 *eq.*), [(**1b**)(**1b'**)Zn][OTf]$_2$ (~0.5 mM, 1 *eq.*) and [(**1b'**)$_2$Zn][OTf]$_2$ (~0.25 mM, 1 *eq.*) in $CDCl_3$ (0.50 mL). The mixture was heated at 40 ˚C for 1 d. After the mixture was cooled to room temperature, $^1H$ NMR spectrum was measured for the solution and showed that the composition ratio of complexes was not changed.

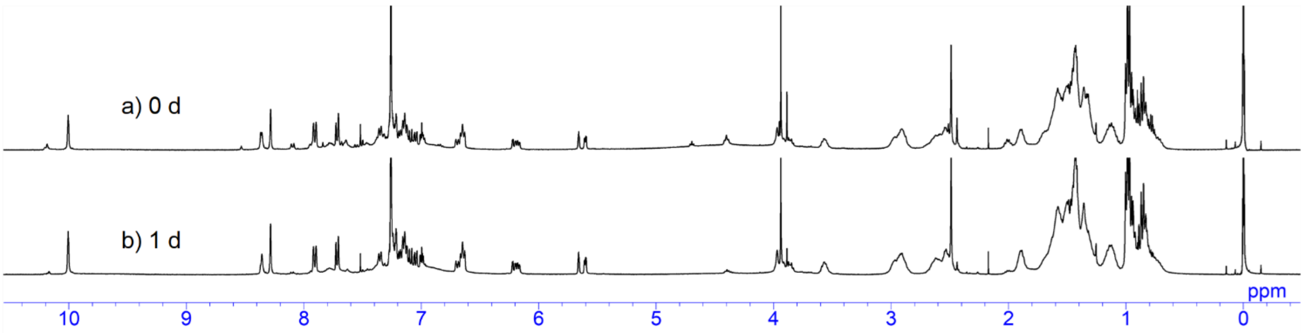


Figure S10. $^1H$ NMR spectra of a mixture of [(**1b**)$_2$Zn][OTf]$_2$, [(**1b**)(**1b'**)Zn][OTf]$_2$ and [(**1b'**)$_2$Zn][OTf]$_2$ after a) just after preparation (0 d) and b) after 1 d ($CDCl_3$, 400 MHz, 298 K).

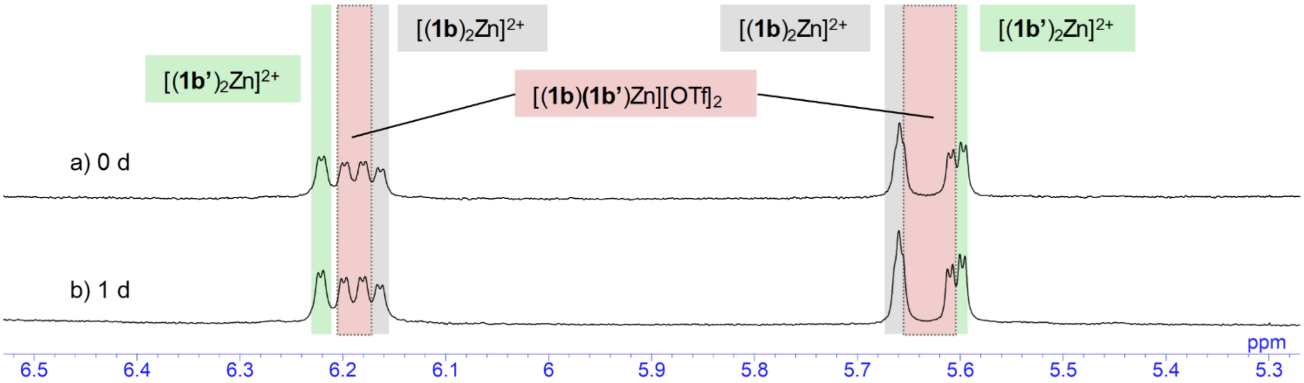


Figure S11. $^1H$ NMR spectra (5.3-6.5 ppm) of a mixture of [(**1b**)$_2$Zn][OTf]$_2$, [(**1b**)(**1b'**)Zn][OTf]$_2$ and [(**1b'**)$_2$Zn][OTf]$_2$ a) just after preparation (0 d) and b) after 1 d ($CDCl_3$, 400 MHz, 298 K).

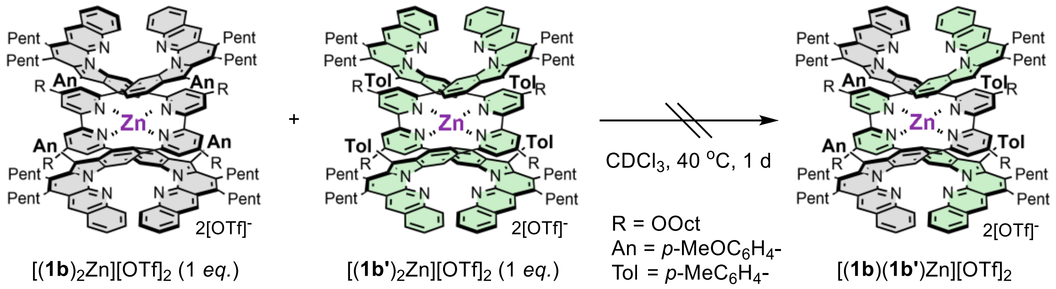


Scheme S3. **The experiments B**: An NMR tube was charged with [(**1b**)$_2$Zn][OTf]$_2$ in $CDCl_3$ (0.5 mM, 0.25 mL, 1 *eq.*) and [(**1b**)$_2$Zn][OTf]$_2$ in $CDCl_3$ (0.5 mM, 0.25 mL, 1 *eq.*). The mixture was heated at 40 ˚C for 1 d. After the reaction mixture was cooled to room temperature, then $^1$H NMR spectrum was measured for the solution and showed that [(**1b**)(**1b'**)Zn][OTf]$_2$ was not detected (Figure S12, Figure S13).

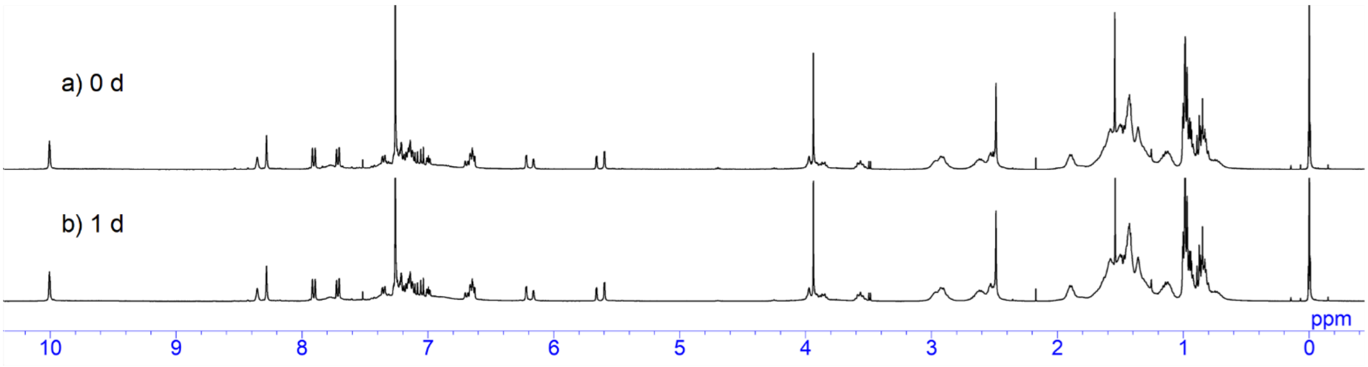


Figure S12. $^1$H NMR spectra of a mixture of [(**1b**)$_2$Zn][OTf]$_2$ and [(**1b**)$_2$Zn][OTf]$_2$ after a) just after preparation (0 d) and b) after 1 d ($CDCl_3$, 400 MHz, 298 K).

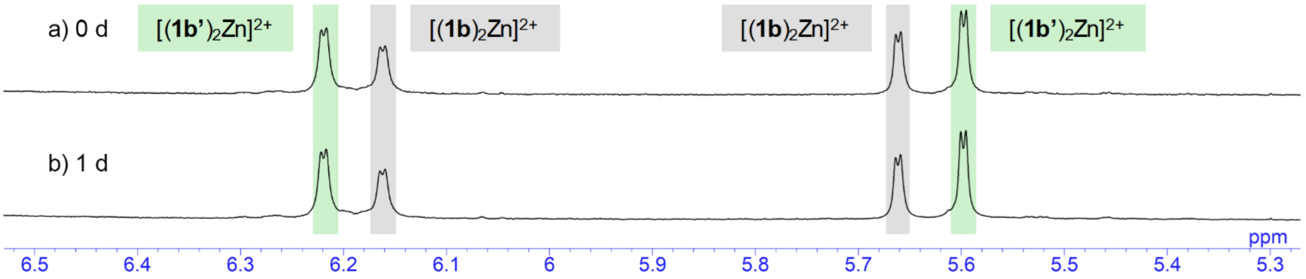


Figure S13. $^1$H NMR spectra (5.3-6.5 ppm) of a mixture of [(**1b**)$_2$Zn][OTf]$_2$ and [(**1b**)$_2$Zn][OTf]$_2$ a) just after preparation (0 d) and b) after 1 d ($CDCl_3$, 400 MHz, 298 K).

## 6. Investigation of conformational isomers of $[(\mathbf{1b})_2Zn][OTf]_2$ by EXSY and DOSY

The exchange rates between the double-helical form and the open forms of $[(\mathbf{1b})_2Zn][OTf]_2$ were estimated by 2D-EXSY[S7].

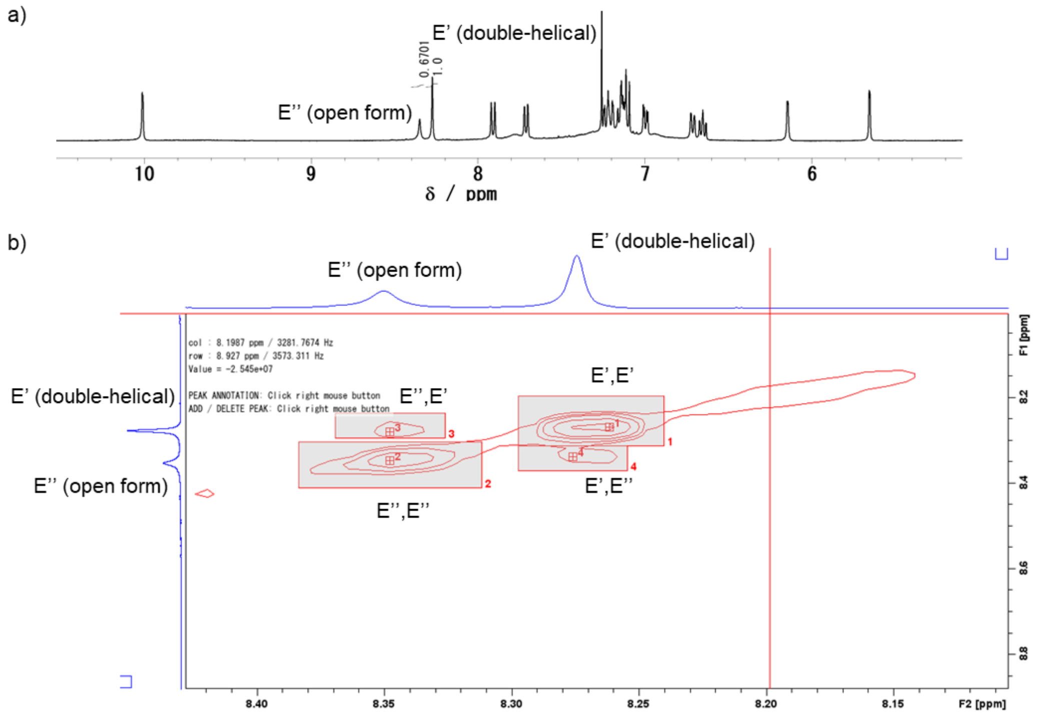


Figure S14. a) $^{1}$H NMR of $[(\mathbf{1b})_2Zn][OTf]_2$ (in $CDCl_3$, 298 K, 400 MHz). b) 2D-EXSY (ROESY: mixing time = 1.0 s) of $[(\mathbf{1b})_2Zn][OTf]_2$ (in $CDCl_3$, 298 K, 400 MHz).

Exchange rates can be obtained by using the equations shown *eq.* S1 and *eq.* S2, where $I_{E'E'}$ and $I_{E''E''}$ are the diagonal peak intensities and $I_{E'E''}$ and $I_{E''E'}$ are the cross-peak intensities (Table S 2). *k* is the sum of the forward, $k_{E'E''}$, and backward, $k_{E''E'}$, pseudo-first order rate constants for the exchange process (*eq.* S3). $k_{E'E''}X_{E'}$ and $k_{E''E'}X_{E''}$ are equal at equilibrium (*eq.* S4) and $X_{E'}$ and $X_{E''}$ were determined by analyzing the integral ratio of the $^{1}$H NMR signal (Table S3) due to the exchange rate constant $k_{E'E''}$ and $k_{E''E'}$ can be determined. By using a modified Eyring equation (*eq.* S5), the free energy of activation $\Delta G^{\ddagger}$ can be determined.

$$r = 4X_{\mathrm{E'}}X_{\mathrm{E''}}\frac{(I_{\mathrm{E'E'}} - I_{\mathrm{E''E''}})}{(I_{\mathrm{E'E''}} - I_{\mathrm{E''EE''}})} - (X_{\mathrm{E'}} - X_{\mathrm{E''}})^2 \qquad (eq.\mathrm{S1})$$

$$k = \frac{1}{t_{\mathrm{m}}}\ln\frac{r+1}{r-1} \qquad (eq.\mathrm{S2})$$

∴ $r = 6.163$, $k = 0.327$ (s$^{-1}$), $k_{E'E''} = 0.131$ (s$^{-1}$), $k_{E''E'} = 0.196$ (s$^{-1}$) ($CDCl_3$, 298 K)

Table S 2. The diagonal peak intensities and the cross-peak intensities.

| | F1 (ppm) | F2 (ppm) | Intensity |
|---|---|---|---|
| E'E' | 8.268 | 8.262 | 9090562231 |
| E"E" | 8.346 | 8.348 | 3862563823 |
| E'E" | 8.278 | 8.348 | 1060644201 |
| E"E' | 8.336 | 8.276 | 946271611 |

Table S3. The mole fraction of the double-helical form and the open forms of [(**1b**)$_2$Zn][OTf]$_2$ determined by analyzing the integral ratio of the $^{1}$H NMR signal.

| area | | mole fraction | |
|---|---|---|---|
| double (E') | open (E") | $X_{E'}$ | $X_{E''}$ |
| 1 | 0.67 | 0.60 | 0.40 |

$$k = k_{\mathrm{E'E''}} + k_{\mathrm{E''E'}} \qquad (eq.\mathrm{S3})$$

$$k_{\mathrm{E'E''}}X_{\mathrm{E'}} = k_{\mathrm{E''E'}}X_{\mathrm{E''}} \qquad (eq.\mathrm{S4})$$

$$\Delta G^{\ddagger} = -RT\ln\frac{hk}{k_B T} \qquad (eq.\mathrm{S5})$$

∴ $\Delta G_{E'E''}^{\ddagger} = 18.65$ (kcal·mol$^{-1}$), $\Delta G_{E''E'}^{\ddagger} = 18.41$ (kcal·mol$^{-1}$) ($CDCl_3$, 298 K)

$T = 298$ (K)

$t_m = 1.0$ (s)

$R = 1.987$ (cal·K$^{-1}$·mol$^{-1}$)

$k_B = 3.30 \times 10^{-24}$ (cal·K$^{-1}$)

$h = 1.58 \times 10^{-34}$ (cal·s)

The hydrodynamic radius $r$ was obtained by substituting the diffusion coefficient $D$ obtained from DOSY into the Storkes-Einstein equation (*eq.* S 6).

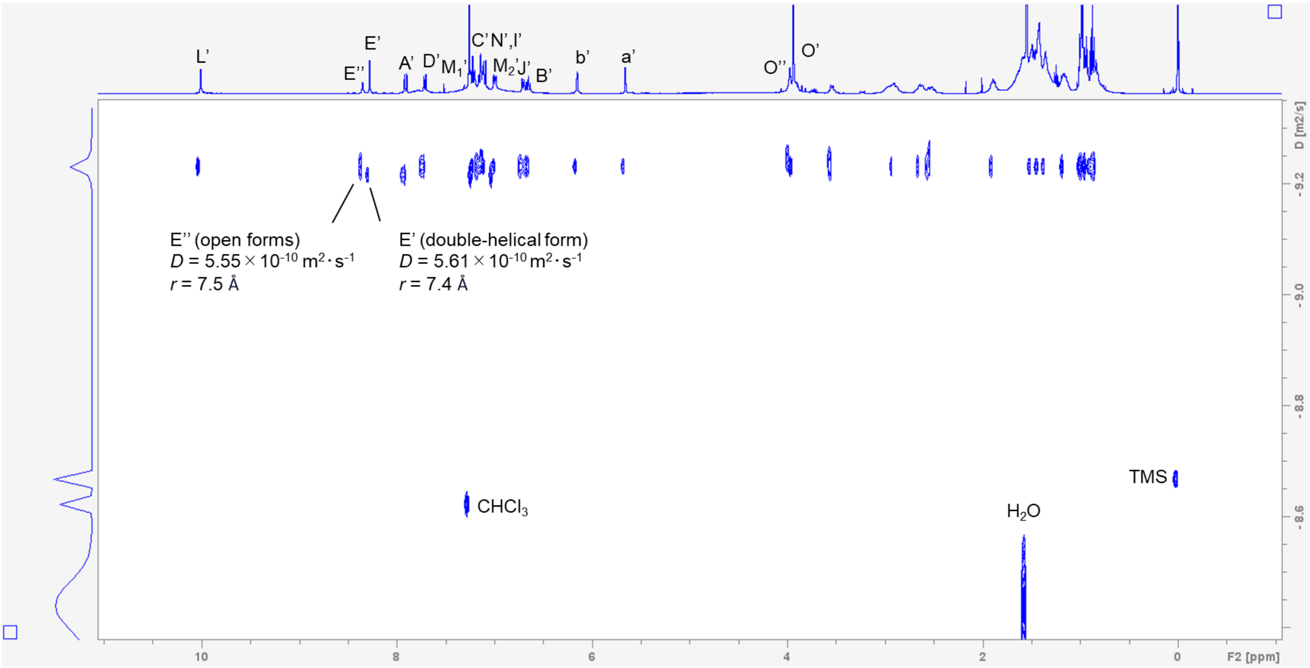


Figure S15. 2D DOSY of [(**1b**)$_2$Zn][OTf]$_2$ ($CDCl_3$, 298 K).

$$D = \frac{k_B T}{6\pi\eta r} \qquad (eq. S6)$$

∴ E'' (open forms): $D = 5.55 \times 10^{-10}$ ($m^2 \cdot s^{-1}$), $r = 7.5$ Å

E' (double-helical form): $D = 5.61 \times 10^{-10}$ ($m^2 \cdot s^{-1}$), $r = 7.4$ Å

$\eta_{CDCl3} = 5.28 \times 10^{-4}$ ($kg \cdot m^{-1} \cdot s^{-1}$)[S6]

$k_B = 1.83 \times 10^{-23}$ ($m^2 \cdot kg \cdot s^{-1} \cdot K^{-1}$)

$\pi = 3.14$

$T = 298$ (K)

**Conformational isomer candidates for the open forms**

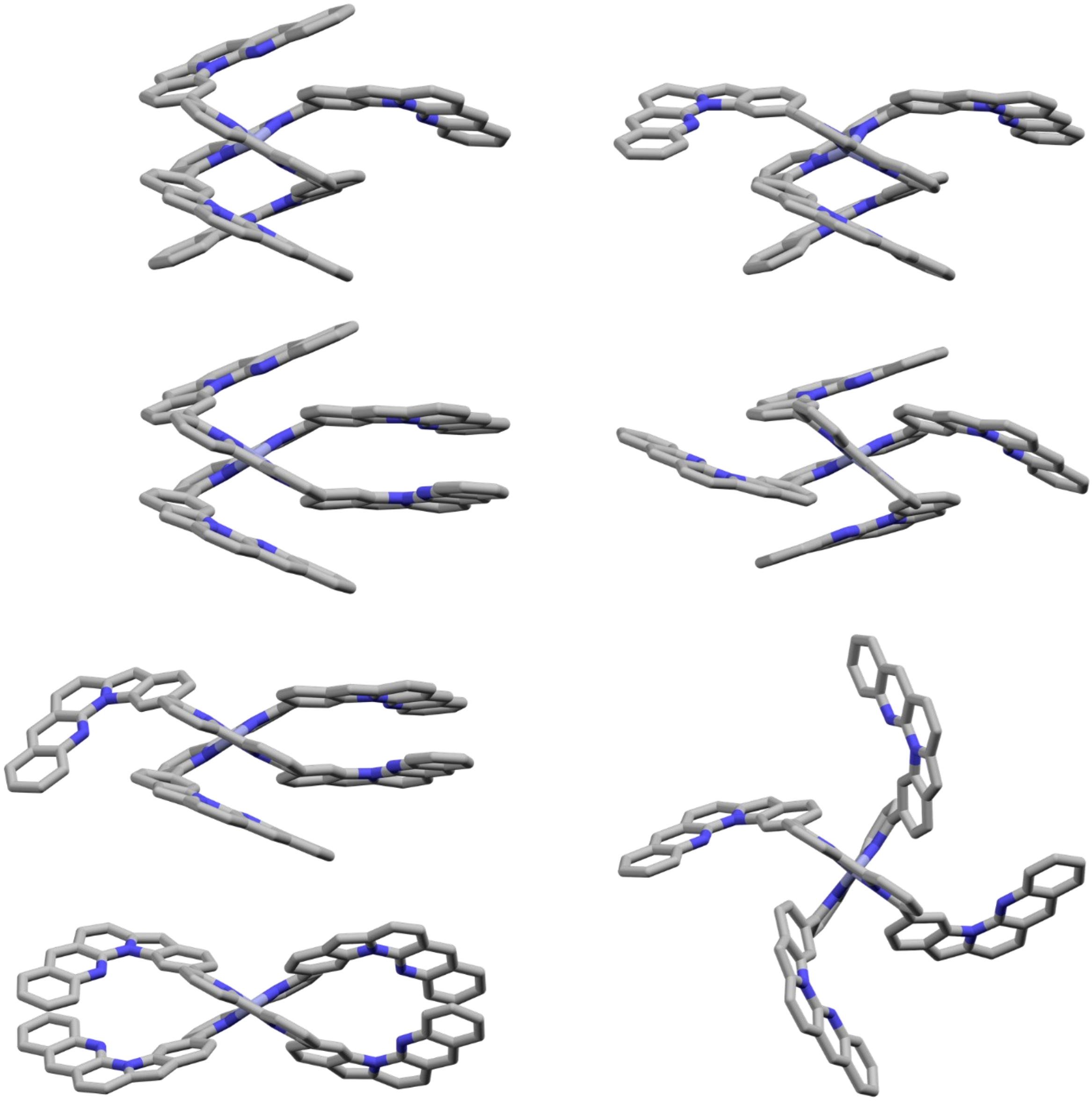

Figure S16. Molecular models of the expected open forms (Hydrogen atoms and side chains are omitted for clarity).

# 7. VT NMR and van't Hoff plots for [(1b)$_2$Zn][OTf]$_2$

General method.

[(**1b**)$_2$Zn][OTf]$_2$ : To an NMR tube was charged with **1b** (1.4 mg, 1 µmol) and $CDCl_3$ (0.50 mL) was added $Zn(OTf)_2$ in acetone-$d_6$ (50 mM, 10 µL, 0.5 µmol, 0.5 eq.) and MS3A (10 mg). After the NMR tube was stirred for 1 min, the $^1$H NMR spectrum of the resulting mixture was recorded, which confirmed the formation of [(*R*)-(**1c**)$_2$Zn][OTf]$_2$. The solution was concentrated in *vacuo*. and subsequently solvents (0.50 mL) were added to the NMR tube. The solution of [(**1b**)$_2$Zn][OTf]$_2$ (1.0 mM) was subjected to VT NMR analyses.

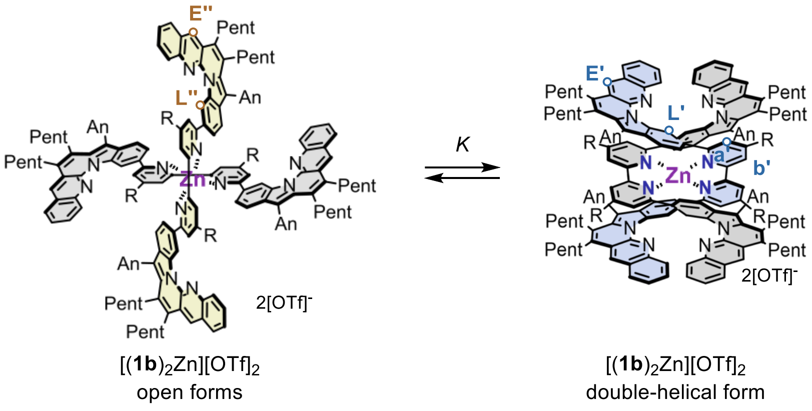


Table S4. Solvent-dependence of thermodynamic parameters in [(**1b**)$_2$Zn][OTf]$_2$

| *solvent* | Δ*H* (kcal·mol$^{-1}$) | Δ*S* (cal·mol$^{-1}$·K$^{-1}$) | Δ*G* (298 K) (kcal·mol$^{-1}$) | *K* (298 K) |
|---|---|---|---|---|
| $CDCl_3/CD_3OD$ = 2/1 | -8.2 ± 2.0 | -25.0 ± 6.5 | -0.7 ± 2.8 | 3.5 |
| $CDCl_3/CD_3CN$ = 2/1 | -5.2 ± 1.0 | -15.3 ± 3.0 | -0.7 ± 1.3 | 3.2 |
| $CDCl_3$ | -2.4 ± 0.3 | -7.1 ± 1.0 | -0.3 ± 0.4 | 1.8 |
| THF-$d_8$ | -1.9 ± 0.3 | -5.5 ± 1.0 | -0.3 ± 0.4 | 1.6 |
| toluene-$d_8$ | -1.7 ± 0.2 | -5.1 ± 0.7 | -0.2 ± 0.3 | 1.4 |

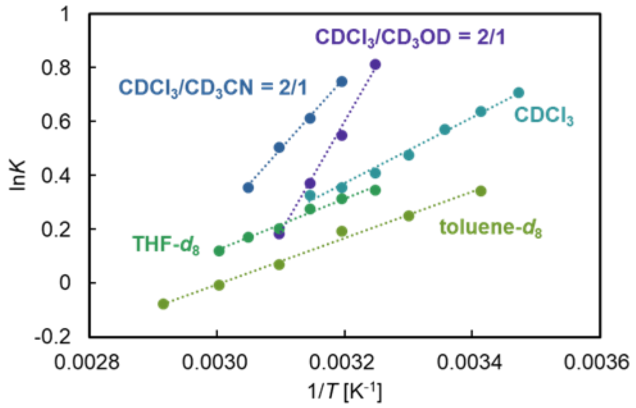


Figure S17. van't Hoff plots for the equilibriums of the open forms and the double-helical form of [(**1b**)$_2$Zn][OTf]$_2$ in several solvents.

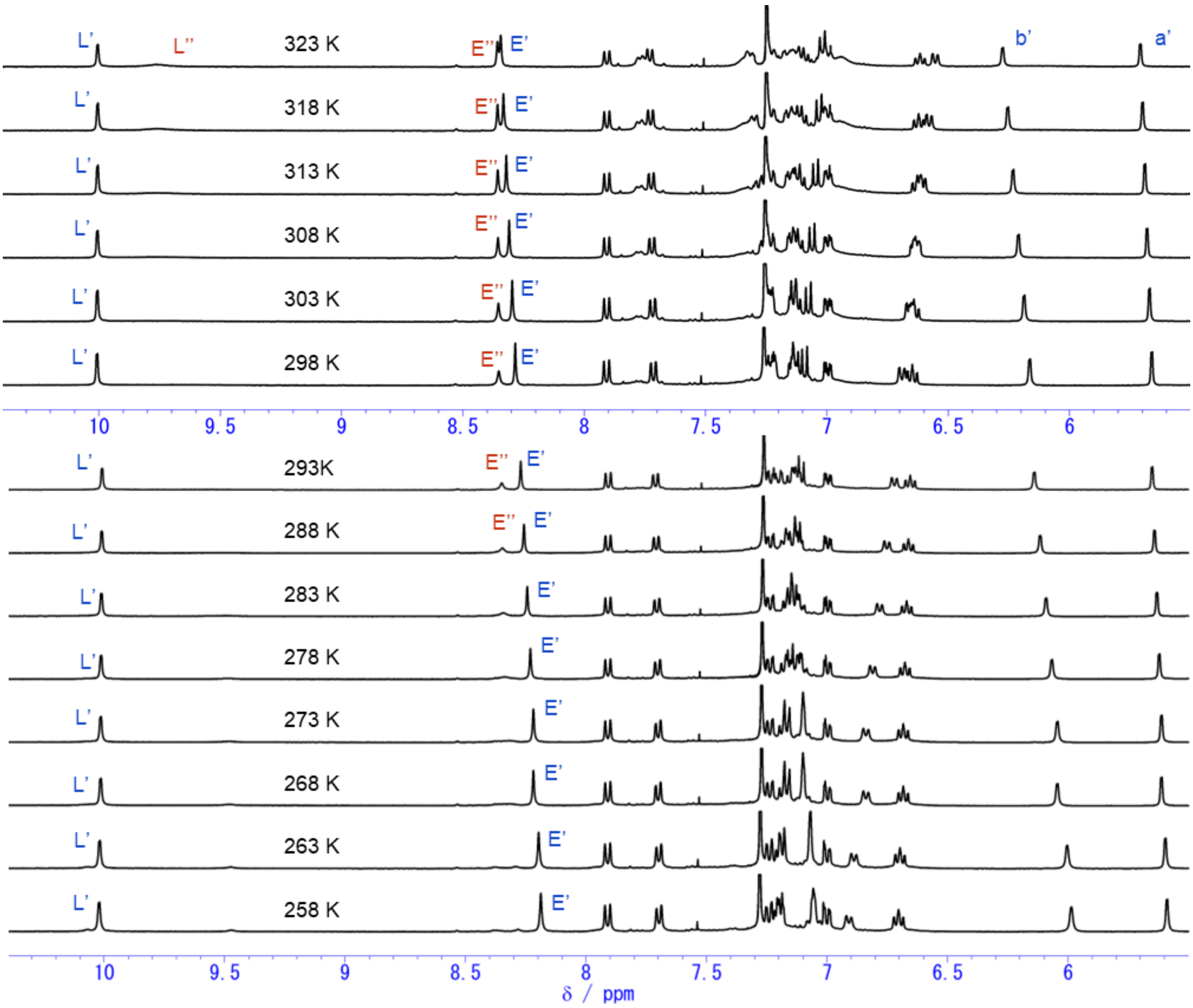


Figure S18. Comparison of representative region of the $^1$H NMR spectra (400 MHz) of [(**1b**)$_2$Zn][OTf]$_2$ in $CDCl_3$ (1.0 μM) at various temperatures.

Table S5. Relative ratio of the open form and the double-helical form of [(**1b**)$_2$Zn][OTf]$_2$, determined by the integrations of $^1$H NMR spectra at various temperatures in $CDCl_3$.

| | integration | | ratio | |
|---|---|---|---|---|
| *temp.* (K) | double-helical (E') | open (E'') | double-helical | open |
| 318 | 1 | 0.72 | 0.58 | 0.42 |
| 313 | 1 | 0.70 | 0.59 | 0.41 |
| 308 | 1 | 0.66 | 0.60 | 0.40 |
| 303 | 1 | 0.62 | 0.62 | 0.38 |
| 298 | 1 | 0.56 | 0.64 | 0.36 |
| 293 | 1 | 0.53 | 0.65 | 0.35 |
| 288 | 1 | 0.49 | 0.67 | 0.33 |

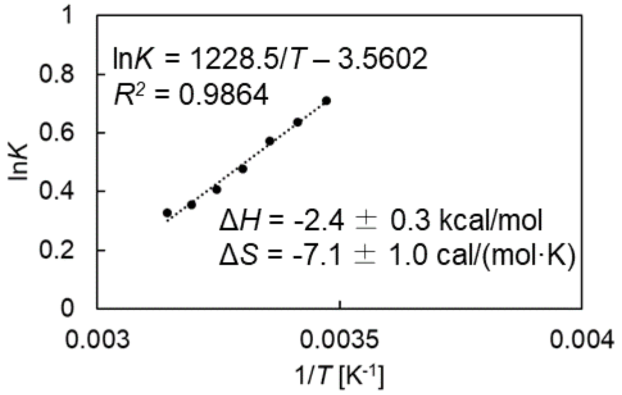


Figure S19. van't Hoff plot for the equilibriums of the open forms and the double-helical form of [(**1b**)$_2$Zn][OTf]$_2$ in $CDCl_3$.

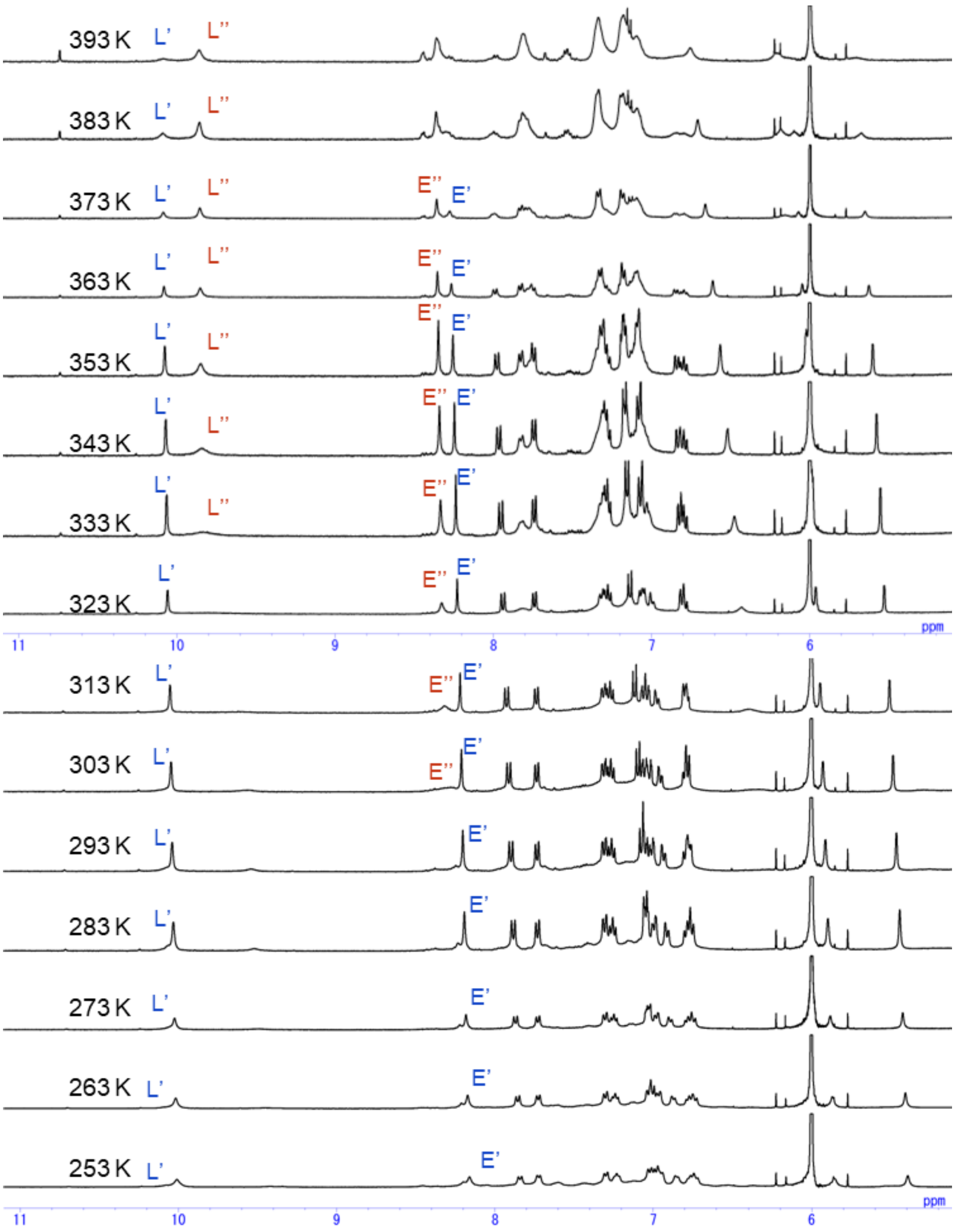


Figure S20. Comparison of representative region of the $^{1}$H NMR spectra (400 MHz) of [(**1b**)$_2$Zn][OTf]$_2$ in $C_2D_2Cl_4$ (1.0 μM) at various temperatures.

Table S6. Relative ratio of the open form and the double-helical form of [(**1b**)$_2$Zn][OTf]$_2$, determined by the integrations of $^1$H NMR spectra at various temperatures in $C_2D_2Cl_4$.

| | integration | | ratio | |
|---|---|---|---|---|
| *temp.* (K) | double-helical (E' or L') | open (E" or L") | double-helical | open |
| 383 | 1 (L') | 2.3 (L") | 0.31 | 0.69 |
| 373 | 0.95 (E') | 2.0 (E") | 0.32 | 0.68 |
| 363 | 1.1 (E') | 1.8 (E") | 0.37 | 0.63 |
| 353 | 1.2 (E') | 1.7 (E") | 0.42 | 0.58 |
| 343 | 1.1 (E') | 1.5 (E") | 0.43 | 0.57 |
| 333 | 1.1 (E') | 1.2 (E") | 0.47 | 0.53 |
| 323 | 1.1 (E') | 0.97 (E") | 0.52 | 0.48 |
| 313 | 1.0 (E') | 0.91 (E") | 0.53 | 0.47 |
| 303 | 1.0 (E') | 0.86 (E") | 0.54 | 0.46 |

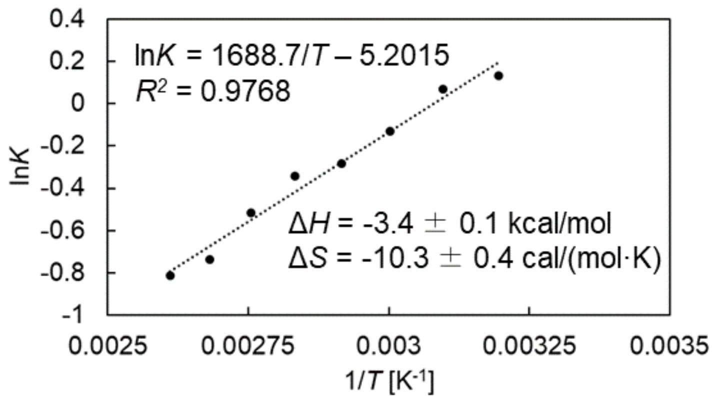


Figure S21. van't Hoff plot for the equilibriums of the open form and the double-helical form of [(**1b**)$_2$Zn][OTf]$_2$ in $C_2D_2Cl_4$.

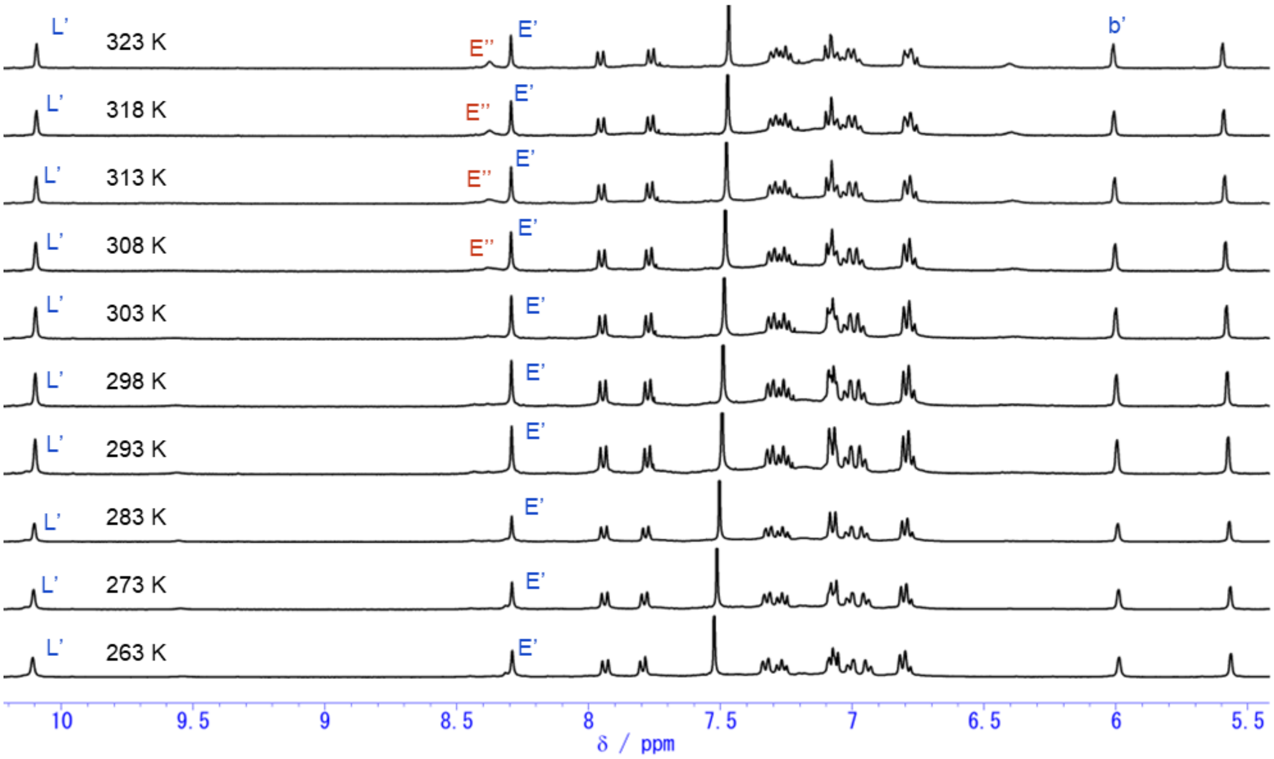


Figure S22. Comparison of representative region of the $^{1}$H NMR spectra (400 MHz) of [(**1b**)$_2$Zn][OTf]$_2$ in $CDCl_3/CD_3OD$ = 2/1 (v/v) (1.0 μM) at various temperatures.

Table S7. Relative ratio of the open form and the double-helical form of [(**1b**)$_2$Zn][OTf]$_2$, determined by the integrations of $^1$H NMR spectra at various temperatures in $CDCl_3/CD_3OD$ = 2/1 (v/v).

| | integration | | ratio | |
|---|---|---|---|---|
| *temp.* (K) | double-helical (E') | open (E'') | double-helical | open |
| 323 | 1 | 0.83 | 0.55 | 0.45 |
| 318 | 1 | 0.69 | 0.59 | 0.41 |
| 313 | 1 | 0.58 | 0.63 | 0.37 |
| 308 | 1 | 0.44 | 0.69 | 0.31 |

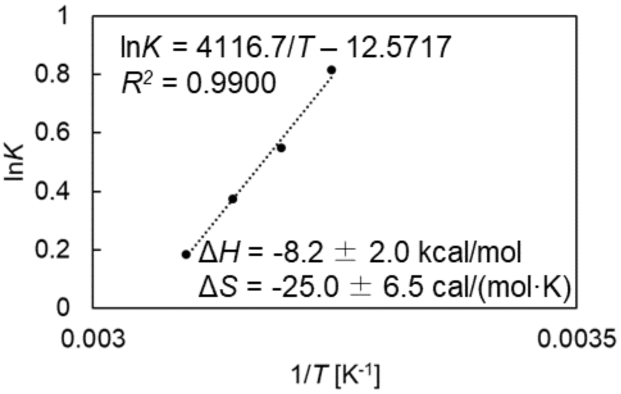


Figure S23. van't Hoff plot for the equilibriums of the open form and the double-helical form of [(**1b**)$_2$Zn][OTf]$_2$ in $CDCl_3/CD_3OD$ = 2/1 (v/v).

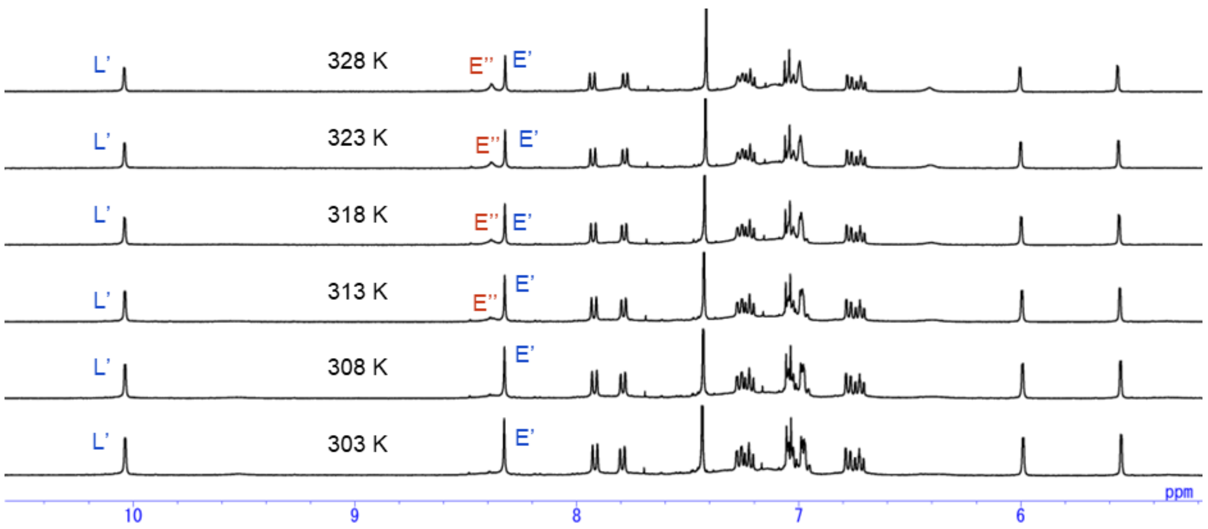


Figure S24. Comparison of representative region of the $^{1}$H NMR spectra (400 MHz) of [(**1b**)$_2$Zn][OTf]$_2$ in $CDCl_3/CD_3CN$ = 2/1 (v/v) (1.0 μM) at various temperatures.

Table S8. Relative ratio of the open form and the double-helical form of [(**1b**)$_2$Zn][OTf]$_2$, determined by the integrations of $^1$H NMR spectra at various temperatures in $CDCl_3/CD_3CN$ = 2/1 (v/v).

| | integration | | ratio | |
|---|---|---|---|---|
| *temp.* (K) | double-helical (E') | open (E'') | double-helical | open |
| 328 | 1 | 0.70 | 0.59 | 0.41 |
| 323 | 1 | 0.60 | 0.62 | 0.38 |
| 318 | 1 | 0.54 | 0.65 | 0.35 |
| 313 | 1 | 0.47 | 0.68 | 0.32 |

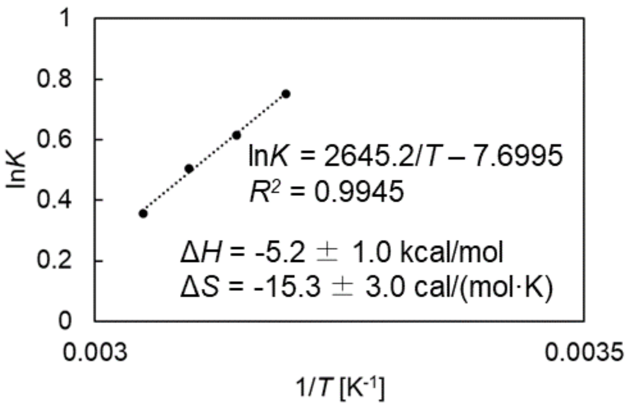


Figure S25. van't Hoff plot for the equilibriums of the open form and the double-helical form of [(**1b**)$_2$Zn][OTf]$_2$ in $CDCl_3/CD_3CN$ = 2/1 (v/v).

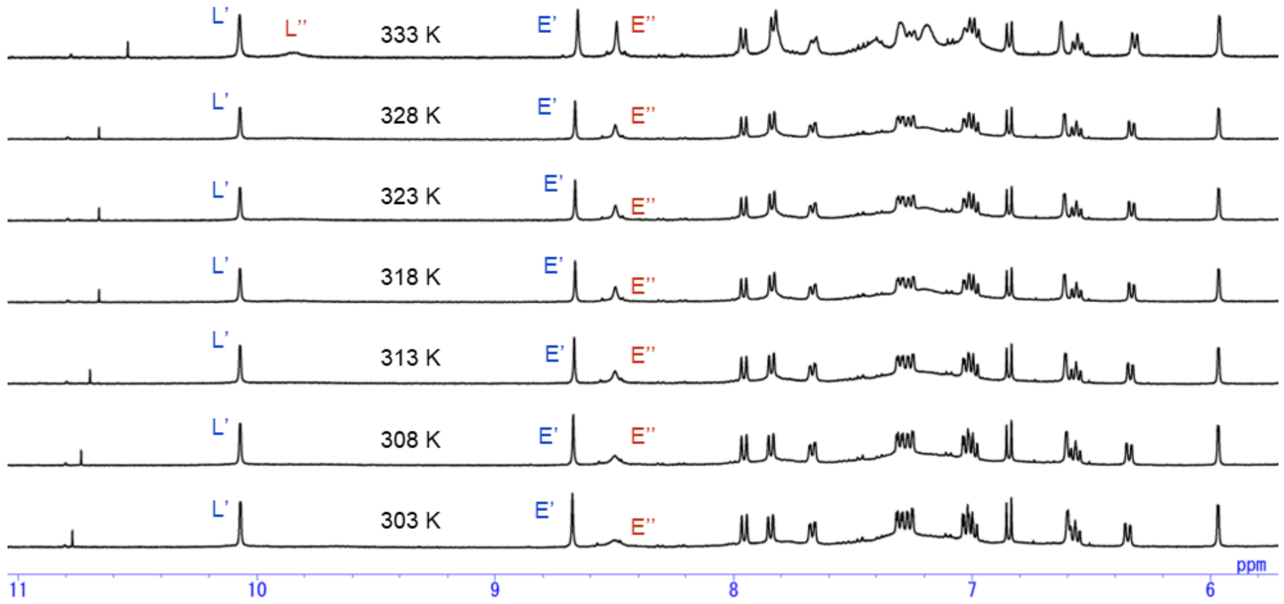


Figure S26. Comparison of representative region of the $^{1}$H NMR spectra (400 MHz) of [(**1b**)$_2$Zn][OTf]$_2$ in THF-$d_8$ (1.0 μM) at various temperatures.

Table S9. Relative ratio of the open form and the double-helical form of [(**1b**)$_2$Zn][OTf]$_2$, determined by the integrations of $^1$H NMR spectra at various temperatures in THF-$d_8$.

| | integration | | ratio | |
|---|---|---|---|---|
| *temp.* (K) | double-helical (E') | open (E'') | double-helical | open |
| 333 | 1 | 0.89 | 0.53 | 0.47 |
| 328 | 1 | 0.84 | 0.54 | 0.46 |
| 323 | 1 | 0.82 | 0.55 | 0.45 |
| 318 | 1 | 0.76 | 0.57 | 0.43 |
| 313 | 1 | 0.73 | 0.58 | 0.42 |
| 308 | 1 | 0.71 | 0.59 | 0.41 |

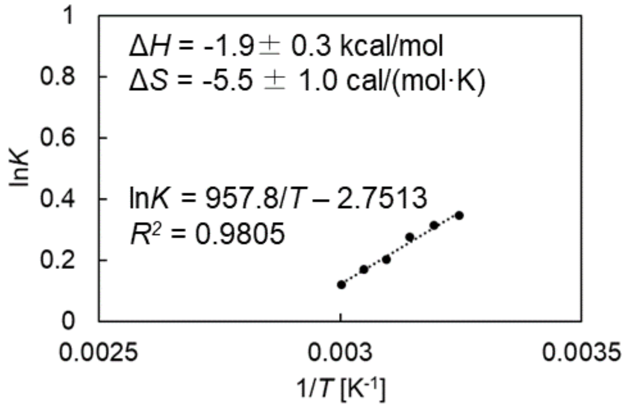


Figure S27. van't Hoff plot for the equilibriums of the open form and the double-helical form of [(**1b**)$_2$Zn][OTf]$_2$ in THF-$d_8$.

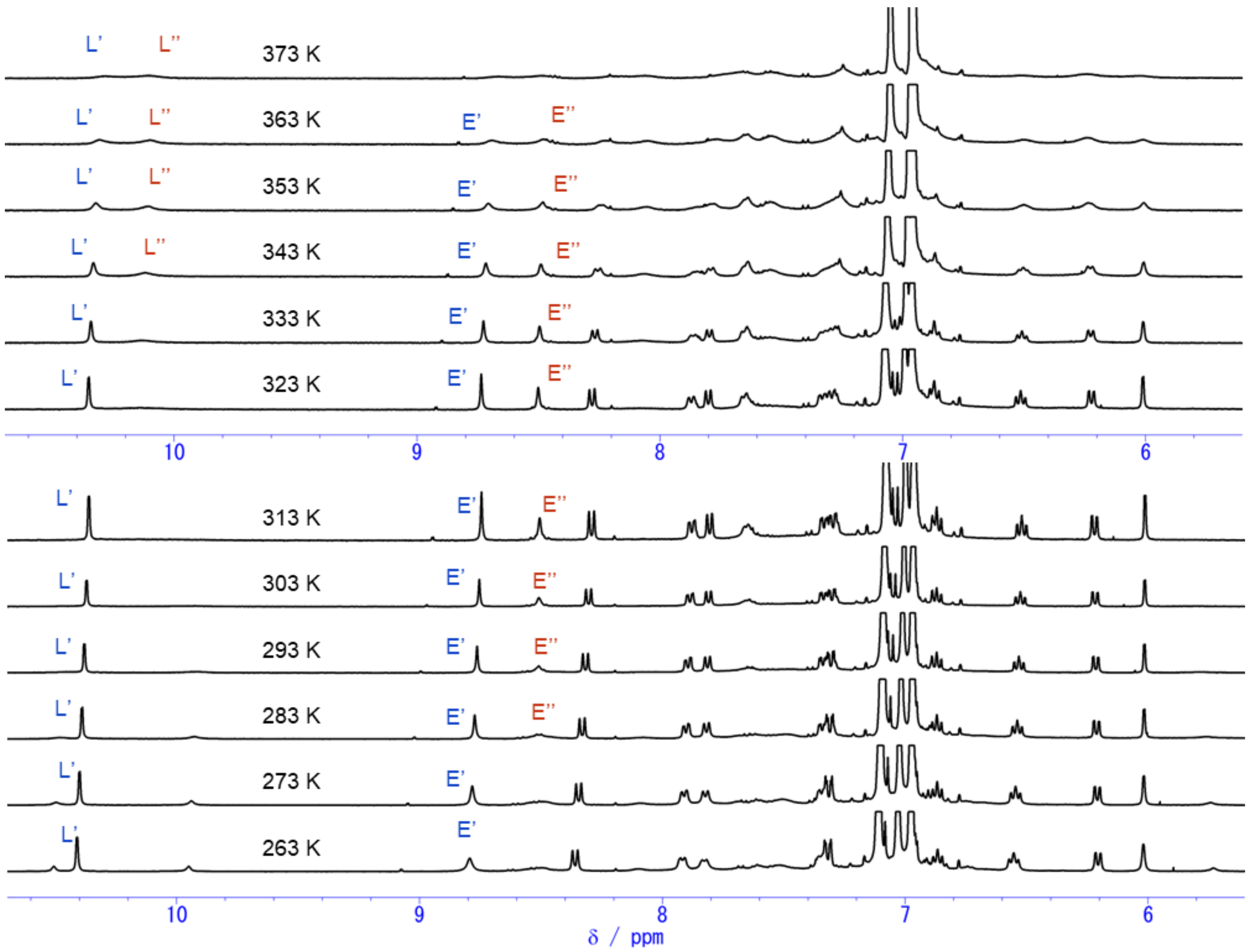


Figure S28. Comparison of representative region of the $^{1}$H NMR spectra (400 MHz) of [(**1b**)$_2$Zn][OTf]$_2$ in Toluene-$d_8$ (1.0 μM) at various temperatures.

Table S10. Relative ratio of the open form and the double-helical form of [(**1b**)$_2$Zn][OTf]$_2$, determined by the integrations of $^1$H NMR spectra at various temperatures in Toluene-$d_8$.

| | integration | | ratio | |
|---|---|---|---|---|
| *temp.* (K) | double-helical (E') | open (E'') | double-helical | open |
| 343 | 1 | 1.1 | 0.48 | 0.52 |
| 333 | 1 | 1.0 | 0.50 | 0.50 |
| 323 | 1 | 0.93 | 0.52 | 0.48 |
| 313 | 1 | 0.82 | 0.55 | 0.45 |
| 303 | 1 | 0.78 | 0.56 | 0.44 |
| 293 | 1 | 0.71 | 0.59 | 0.41 |

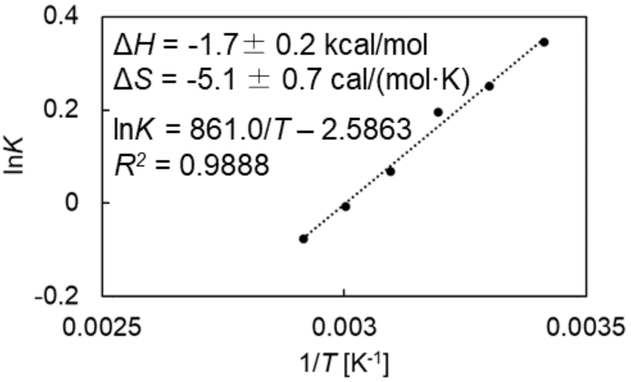


Figure S29. van't Hoff plot for the equilibriums of the open form and the double-helical form of [(**1b**)$_2$Zn][OTf]$_2$ in Toluene-$d_8$.

# 8. UV-vis and CD spectra of (*R*)-(1c) and [(*R*)-(1c)$_2$Zn][OTf]$_2$

**General procedure on UV-vis and CD measurements for [(*R*)-(1c)$_2$Zn][OTf]$_2$**

To an NMR tube was charged with **1c** (1.5 mg, 1 µmol) and $CDCl_3$ (0.50 mL) was added $Zn(OTf)_2$ in acetone-$d_6$ (50 mM, 10 µL, 0.5 µmol, 0.5 *eq.*) and MS3A (10 mg). After the NMR tube was stirred for 1 min, the formation of complex [(*R*)-(**1c**)$_2$Zn][OTf]$_2$ was confirmed by $^1$H NMR (See section 4). The solution of [(*R*)-(**1c**)$_2$Zn][OTf]$_2$ was concentrated and dried in *vacuo* to obtained [(*R*)-(**1c**)$_2$Zn][OTf]$_2$ complex as a yellow solid. [(*R*)-(**1c**)$_2$Zn][OTf]$_2$ was dissolved in various solvent to 5.0 µM, and UV-vis absorption and CD spectra of the prepared solutions were obtained at room temperature.

The CD signal of monometallofoldamers [(*R*)-(**1c**)$_2$Zn][OTf]$_2$ did not decrease for at least several hours at room temperature in the solvents shown in Table 2. Similarly, no decomposition product peaks of monometallofoldamers [(*R*)-(**1c**)$_2$Zn][OTf]$_2$ were observed by NMR in $CDCl_3$, acetone-$d_6$, and toluene-$d_8$ at room temperature for 1 day. On the other hand, [(*R*)-(**1c**)$_2$Zn][OTf]$_2$ was decomplexed in HMPA or in the presence of amines (e.g., $Et_3N$). Although [(*R*)-(**1c**)$_2$Zn][OTf]$_2$ was stable in many solvents shown in Table 2, HPLC analysis for monometallofoldamer [(*R*)-(**1c**)$_2$Zn][OTf]$_2$ was failed due to instability of the complex and the difficulty in selecting suitable solvent conditions for HPLC analysis on a chiral column.

**UV-vis absorption and CD spectra of (*R*)-(1c)**

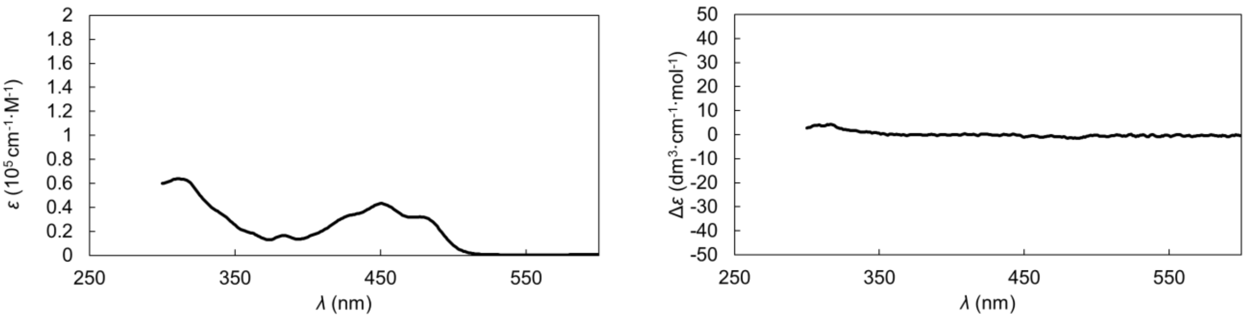


Figure S30. UV-vis absorption and CD spectra of (*R*)-(**1c**) in $CHCl_3$ ([(*R*)-(**1c**)] = 10 µM).

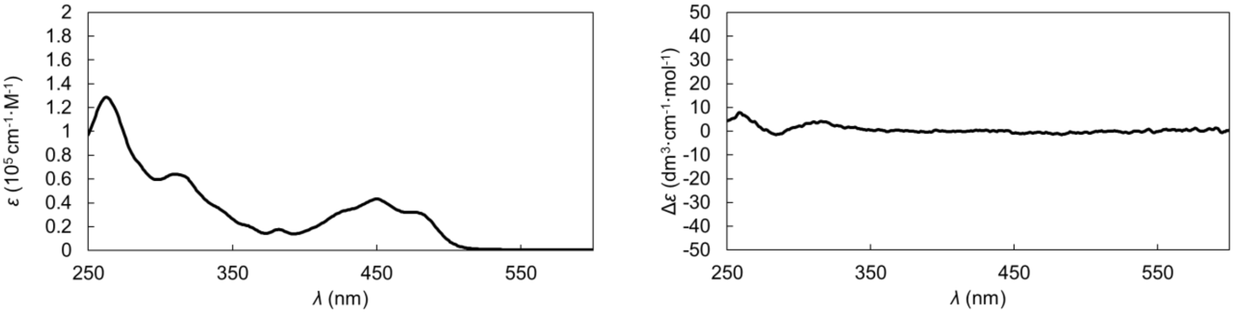


Figure S31. UV-vis absorption and CD spectra of (*R*)-(**1c**) in $CH_2Cl_2$ ([(*R*)-(**1c**)] = 10 µM).

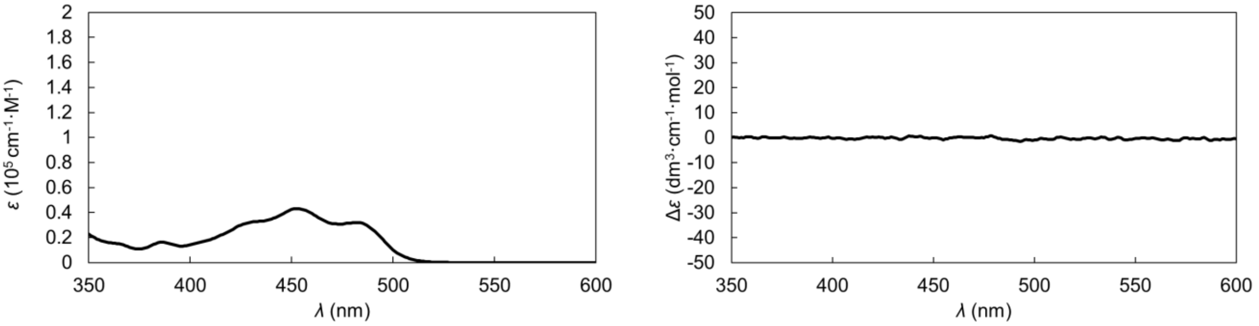


Figure S32. UV-vis absorption and CD spectra of (*R*)-(**1c**) in toluene ([(*R*)-(**1c**)] = 10 μM).

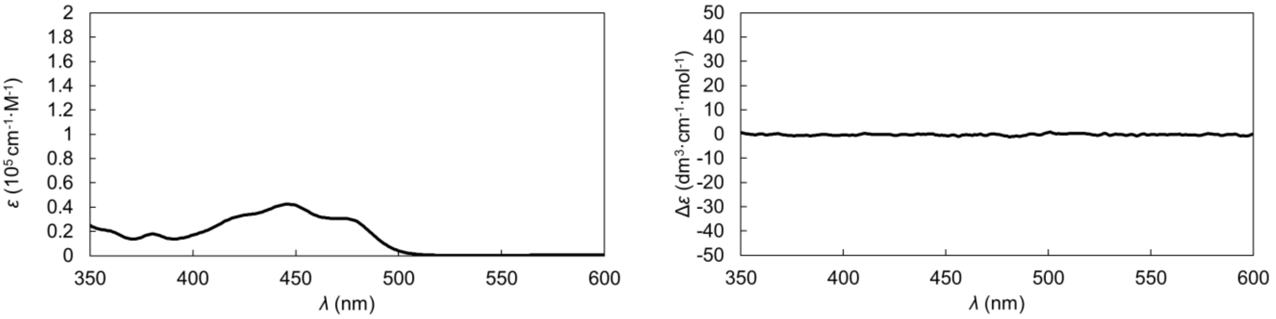


Figure S33. UV-vis absorption and CD spectra of (*R*)-(**1c**) in acetone ([(*R*)-(**1c**)] = 10 μM).

**UV-vis absorption and CD spectra of [(*R*)-(1c)$_2$Zn][OTf]$_2$**

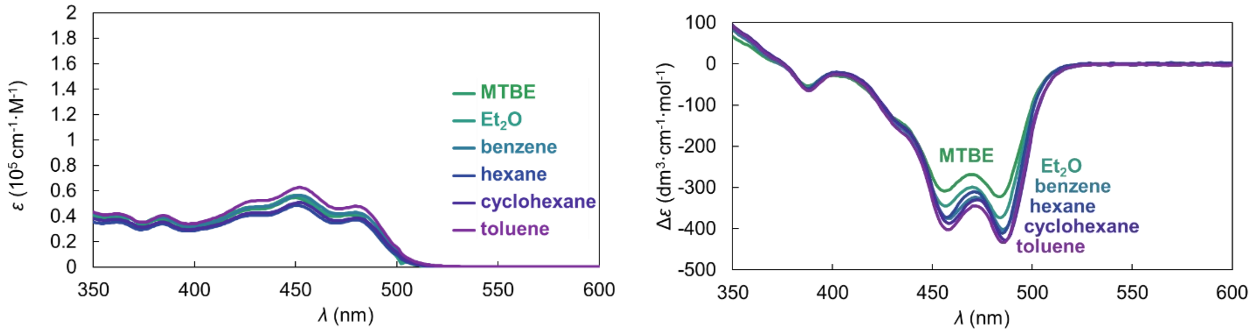


Figure S34. UV-vis absorption and CD spectra of [(*R*)-(**1c**)$_2$Zn][OTf]$_2$ in MTBE (*t*-BuOMe), $Et_2O$, benzene, hexane, cyclohexane and toluene ([(*R*)-(**1c**)$_2$Zn][OTf]$_2$ = 5.0 μM).

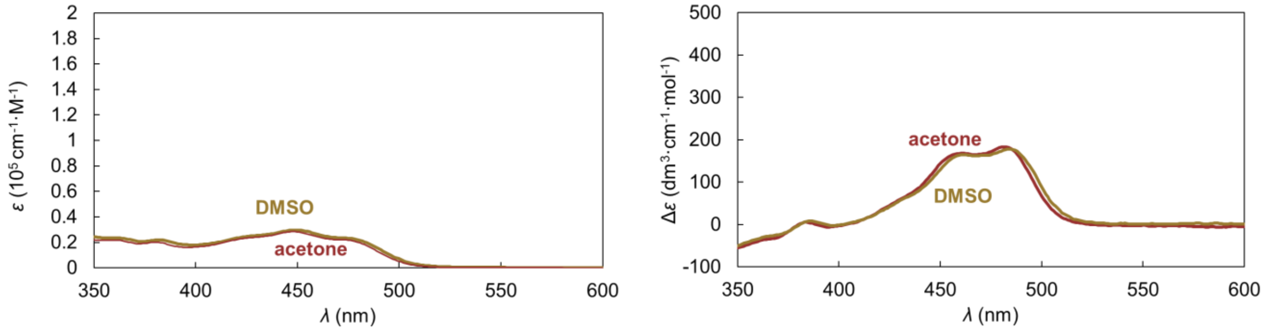


Figure S35. UV-vis absorption and CD spectra of [(*R*)-(**1c**)$_2$Zn][OTf]$_2$ in acetone and DMSO ([(*R*)-(**1c**)$_2$Zn][OTf]$_2$ = 5.0 μM).

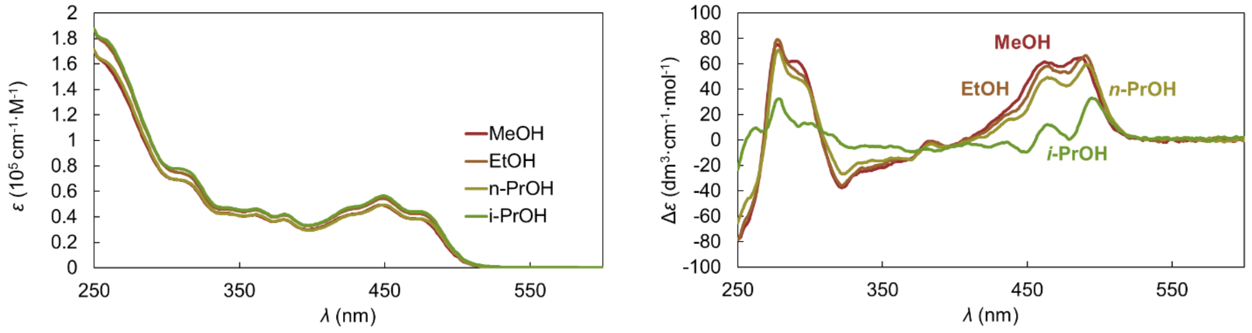


Figure S36. CD spectra of [(*R*)-(**1c**)$_2$Zn][OTf]$_2$ in MeOH, EtOH, *n*-PrOH and *i*-PrOH ([(*R*)-(**1c**)$_2$Zn][OTf]$_2$ = 5.0 μM).

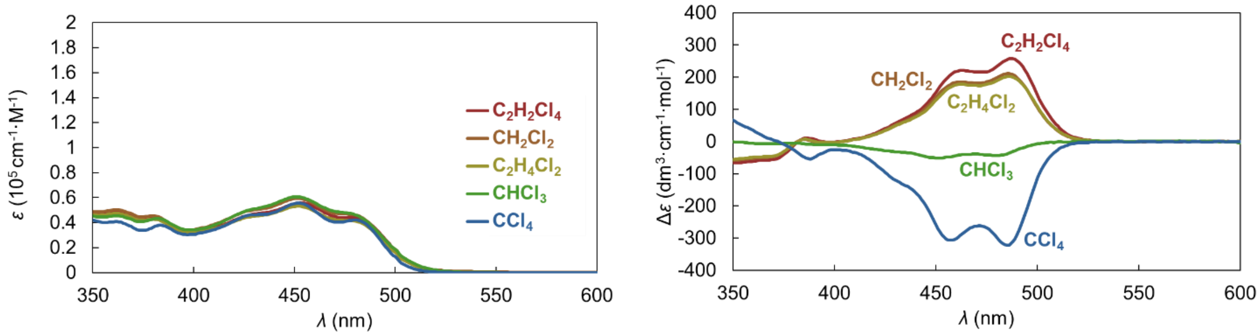


Figure S37. CD spectra of [(*R*)-(**1c**)$_2$Zn][OTf]$_2$ in $C_2H_2Cl_4$, $CH_2Cl_2$, $C_2H_4Cl_3$, $CHCl_3$ and $CCl_4$ ([(*R*)-(**1c**)$_2$Zn][OTf]$_2$ = 5.0 μM).

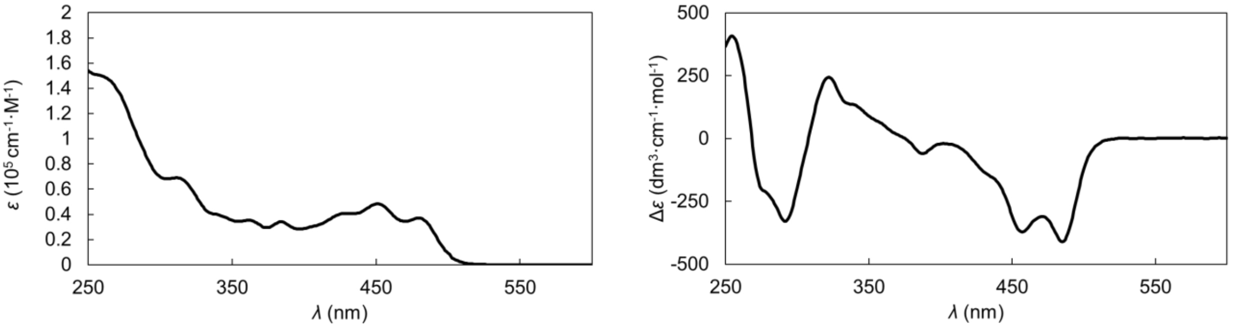


Figure S38. UV-vis absorption and CD spectra of [(*R*)-(**1c**)$_2$Zn][OTf]$_2$ in hexane ([(**1c**)$_2$Zn][OTf]$_2$ = 5.0 μM).

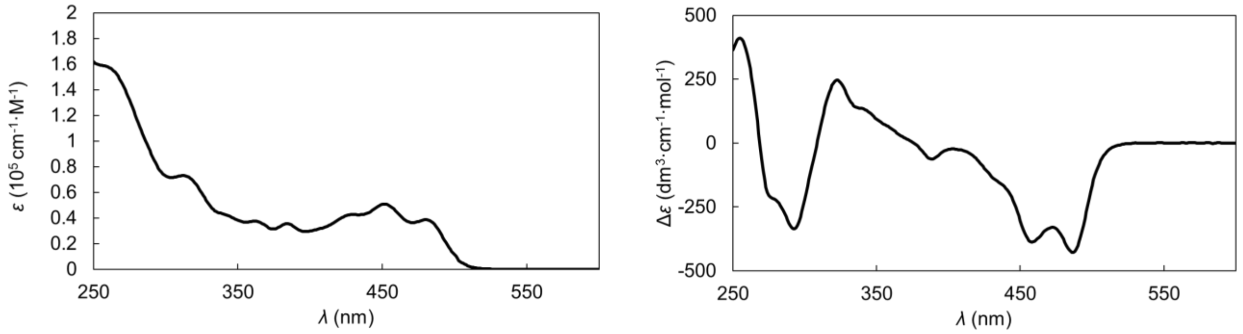


Figure S39.UV-vis absorption and CD spectra of [(*R*)-(**1c**)$_2$Zn][OTf]$_2$ in cyclohexane ([(*R*)-(**1c**)$_2$Zn][OTf]$_2$ = 5.0 μM).

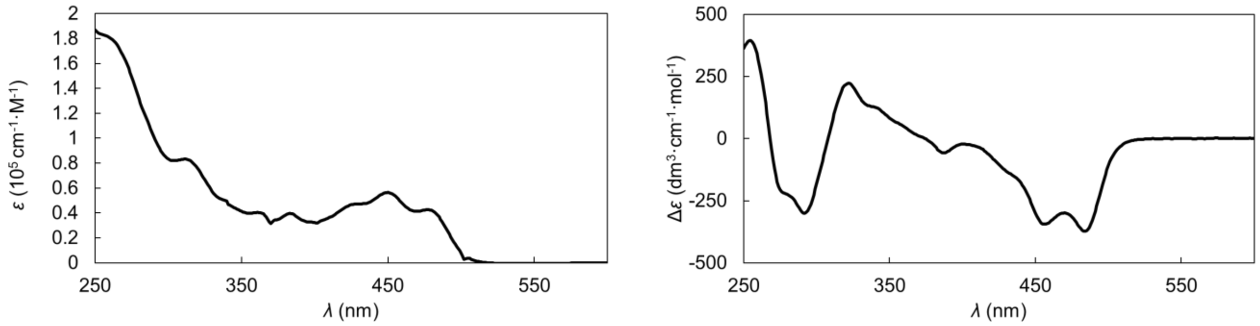


Figure S40. UV-vis absorption and CD spectra of [(*R*)-(**1c**)$_2$Zn][OTf]$_2$ in $Et_2O$ ([(*R*)-(**1c**)$_2$Zn][OTf]$_2$ = 5.0 μM).

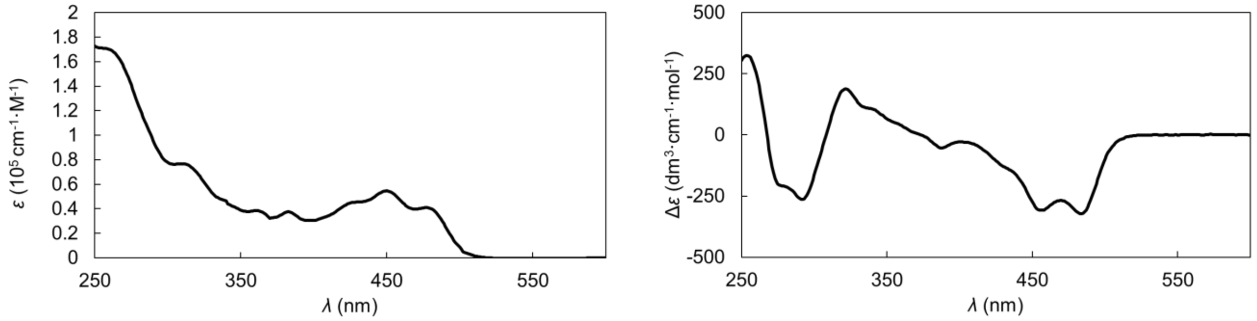


Figure S41. UV-vis absorption and CD spectra of [(*R*)-(**1c**)$_2$Zn][OTf]$_2$ in MTBE ([(*R*)-(**1c**)$_2$Zn][OTf]$_2$ = 5.0 μM).

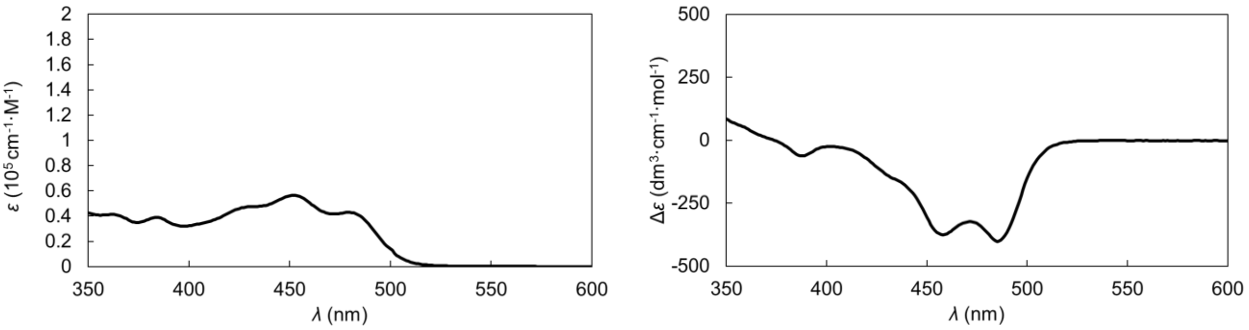


Figure S42. UV-vis absorption and CD spectra of [(*R*)-(**1c**)$_2$Zn][OTf]$_2$ in benzene ([(*R*)-(**1c**)$_2$Zn][OTf]$_2$ = 5.0 μM).

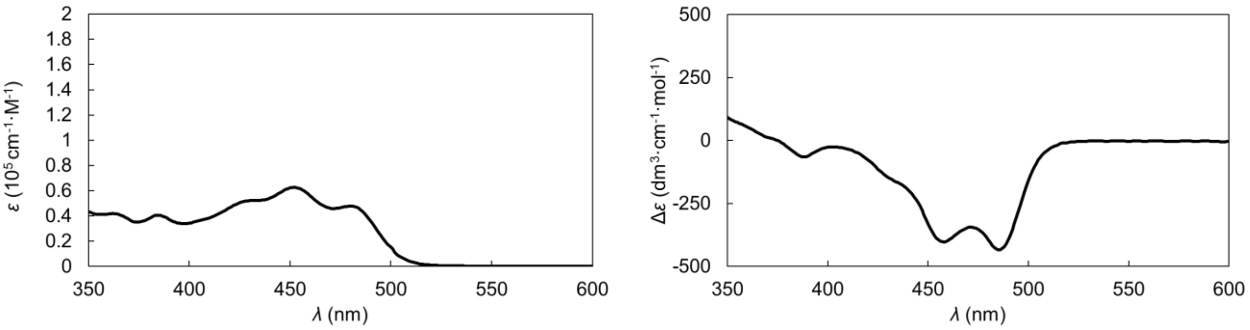


Figure S43. UV-vis absorption and CD spectra of [(*R*)-(**1c**)$_2$Zn][OTf]$_2$ in toluene ([(*R*)-(**1c**)$_2$Zn][OTf]$_2$ = 5.0 μM).

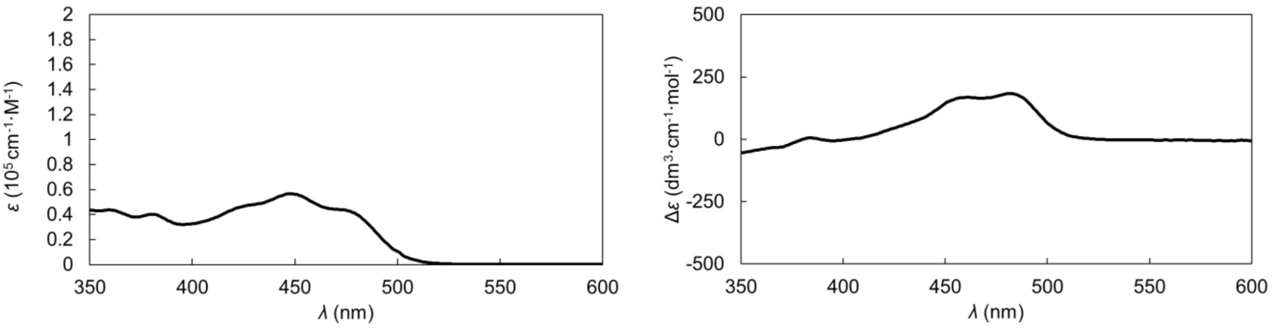


Figure S44. UV-vis absorption and CD spectra of [(*R*)-(**1c**)$_2$Zn][OTf]$_2$ in acetone ([(*R*)-(**1c**)$_2$Zn][OTf]$_2$ = 5.0 μM).

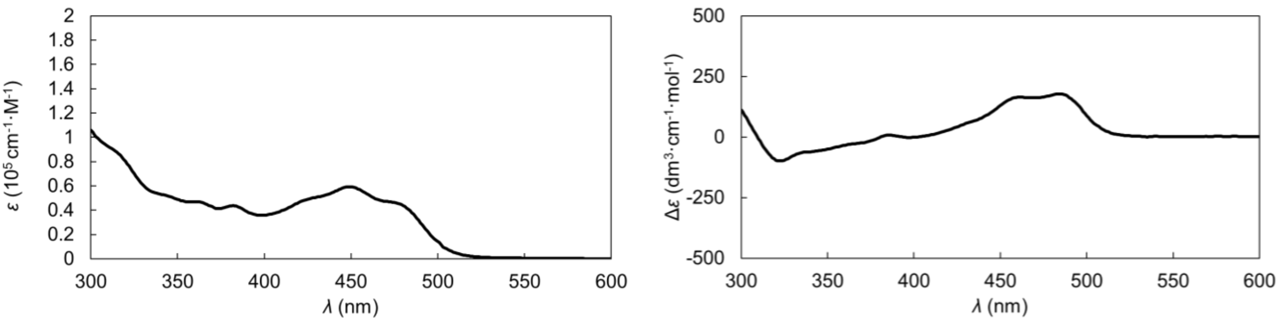


Figure S45. UV-vis absorption and CD spectra of [(*R*)-(**1c**)$_2$Zn][OTf]$_2$ in DMSO ([(*R*)-(**1c**)$_2$Zn][OTf]$_2$ = 5.0 μM).

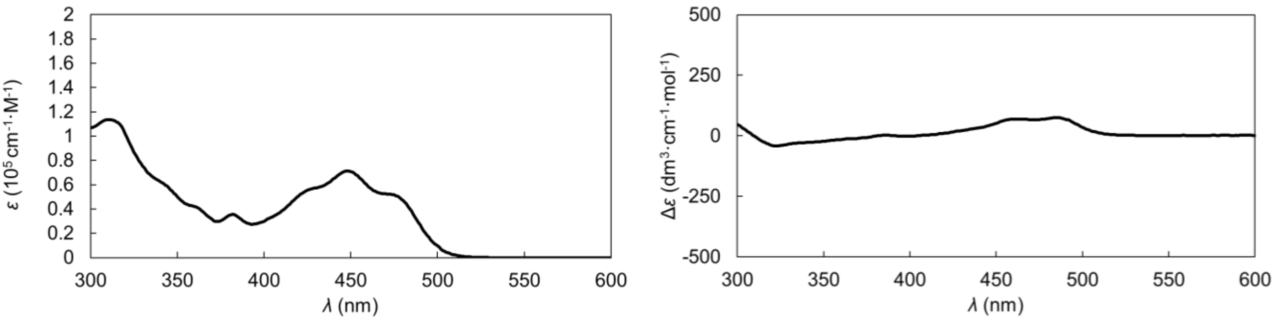


Figure S46. UV-vis absorption and CD spectra of [(*R*)-(**1c**)$_2$Zn][OTf]$_2$ in DMF ([(*R*)-(**1c**)$_2$Zn][OTf]$_2$ = 5.0 μM).

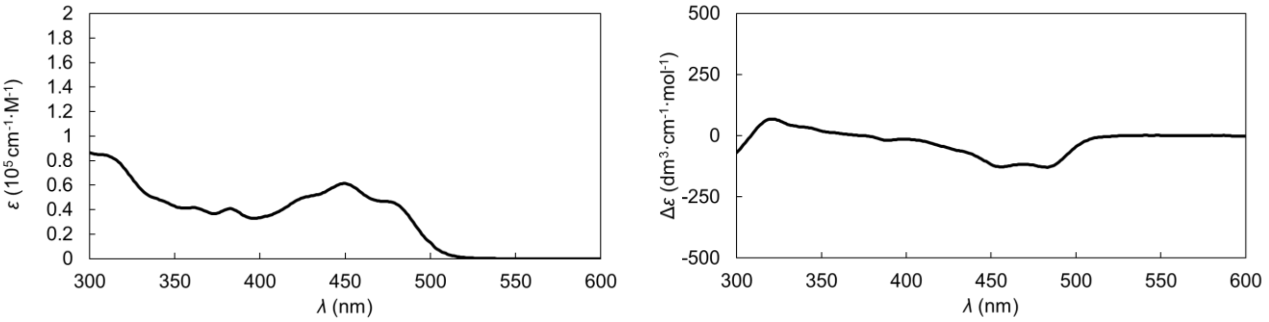


Figure S47. UV-vis absorption and CD spectra of [(*R*)-(**1c**)$_2$Zn][OTf]$_2$ in THF ([(*R*)-(**1c**)$_2$Zn][OTf]$_2$ = 5.0 μM).

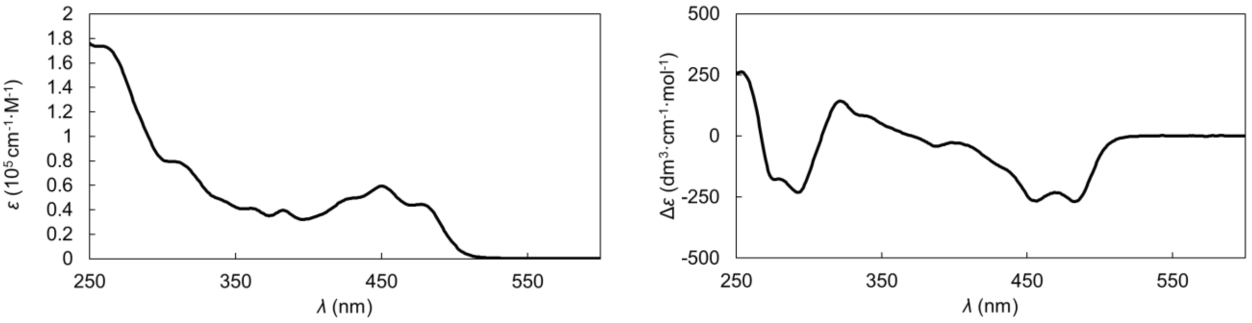


Figure S48. UV-vis absorption and CD spectra of [($R$)-($\mathbf{1c}$)$_2$Zn][OTf]$_2$ in 1,4-dioxane ([($R$)-($\mathbf{1c}$)$_2$Zn][OTf]$_2$ = 5.0 μM).

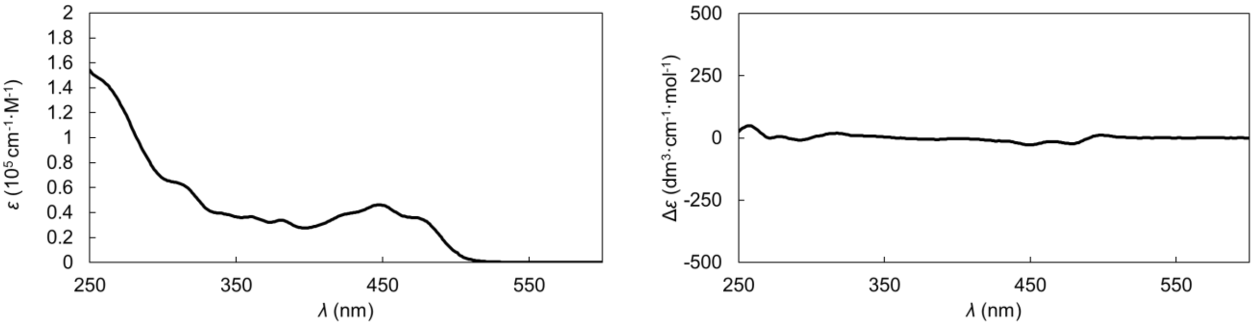


Figure S49. UV-vis absorption and CD spectra of [($R$)-($\mathbf{1c}$)$_2$Zn][OTf]$_2$ in MeCN ([($R$)-($\mathbf{1c}$)$_2$Zn][OTf]$_2$ = 5.0 μM).

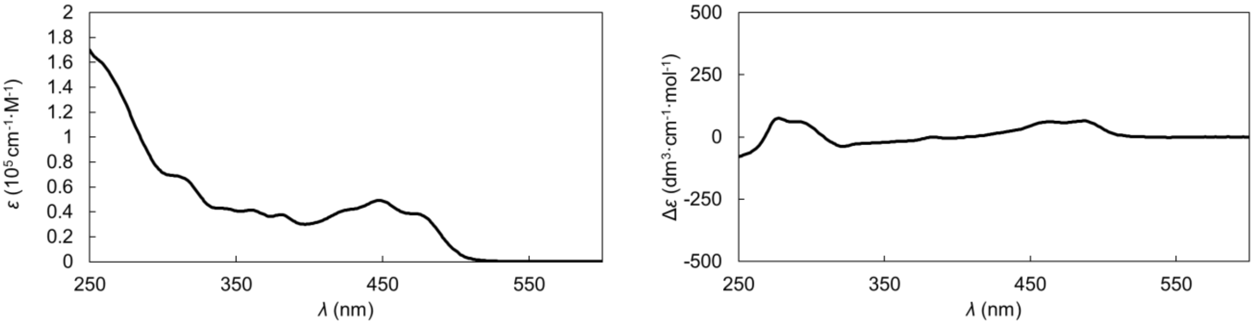


Figure S50. UV-vis absorption and CD spectra of [($R$)-($\mathbf{1c}$)$_2$Zn][OTf]$_2$ in MeOH ([($R$)-($\mathbf{1c}$)$_2$Zn][OTf]$_2$ = 5.0 μM).

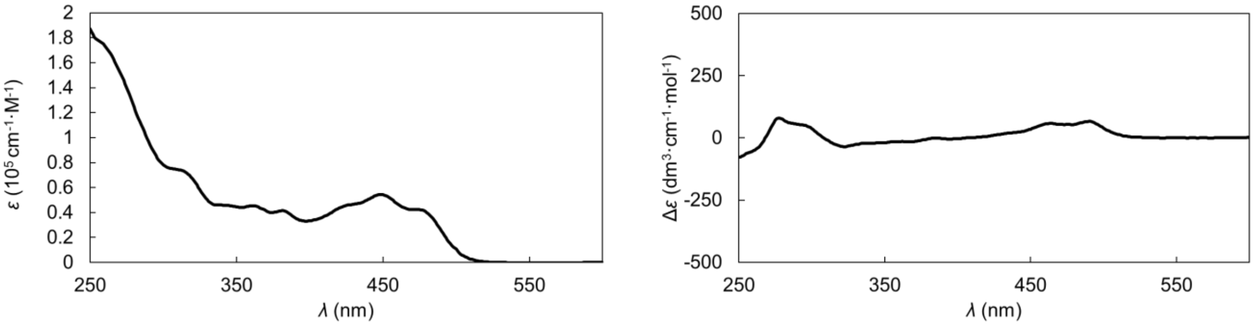


Figure S51. UV-vis absorption and CD spectra of [(*R*)-(**1c**)$_2$Zn][OTf]$_2$ in EtOH ([(*R*)-(**1c**)$_2$Zn][OTf]$_2$ = 5.0 μM).

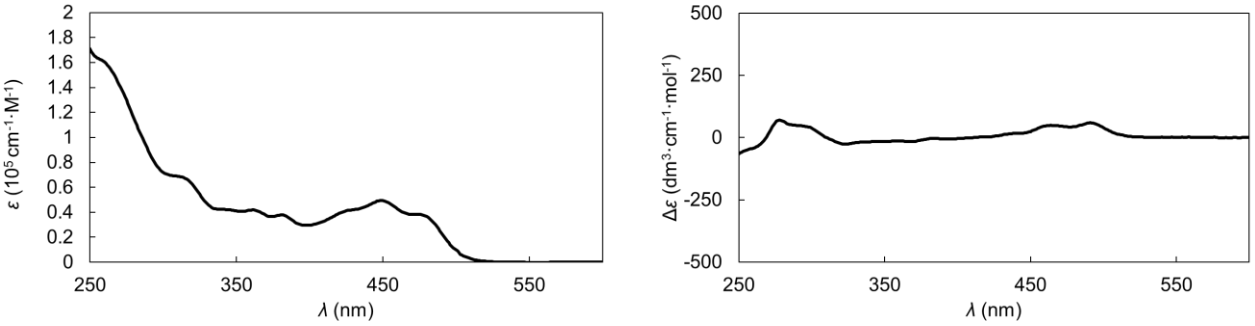


Figure S52. UV-vis absorption and CD spectra of [(*R*)-(**1c**)$_2$Zn][OTf]$_2$ in *n*-PrOH ([(*R*)-(**1c**)$_2$Zn][OTf]$_2$ = 5.0 μM).

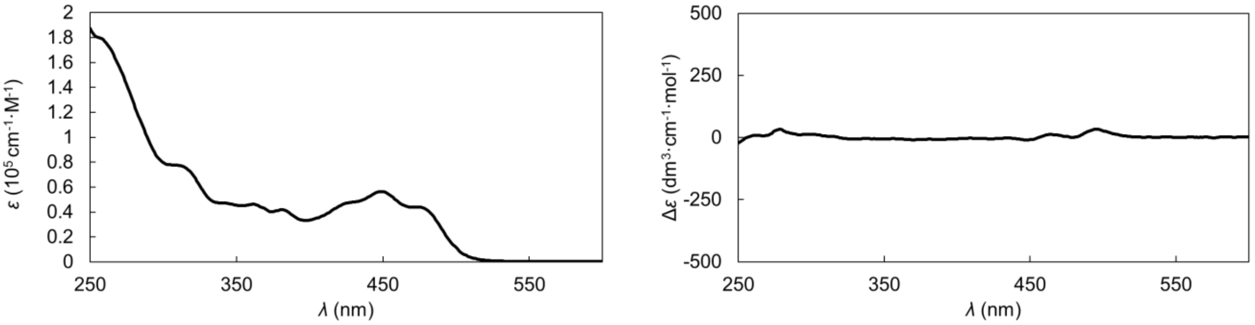


Figure S53. UV-vis absorption and CD spectra of [(*R*)-(**1c**)$_2$Zn][OTf]$_2$ in *i*-PrOH ([(*R*)-(**1c**)$_2$Zn][OTf]$_2$ = 5.0 μM).

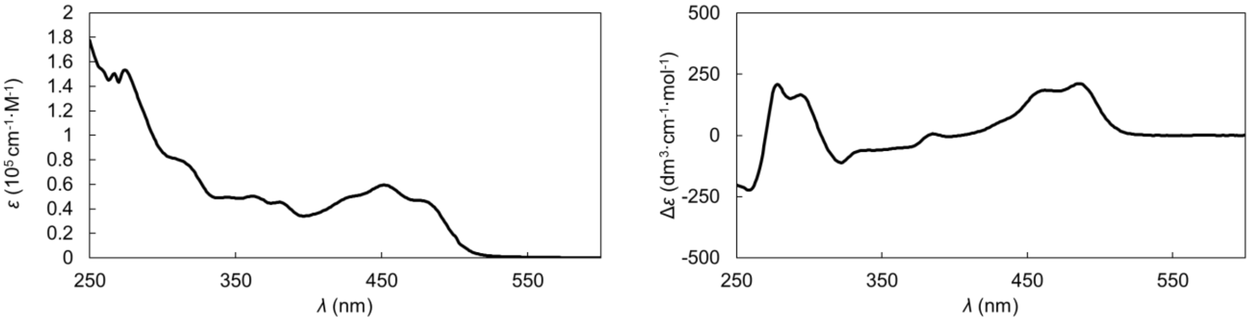


Figure S54. UV-vis absorption and CD spectra of [(*R*)-(**1c**)$_2$Zn][OTf]$_2$ in $CH_2Cl_2$ ([(*R*)-(**1c**)$_2$Zn][OTf]$_2$ = 5.0 μM).

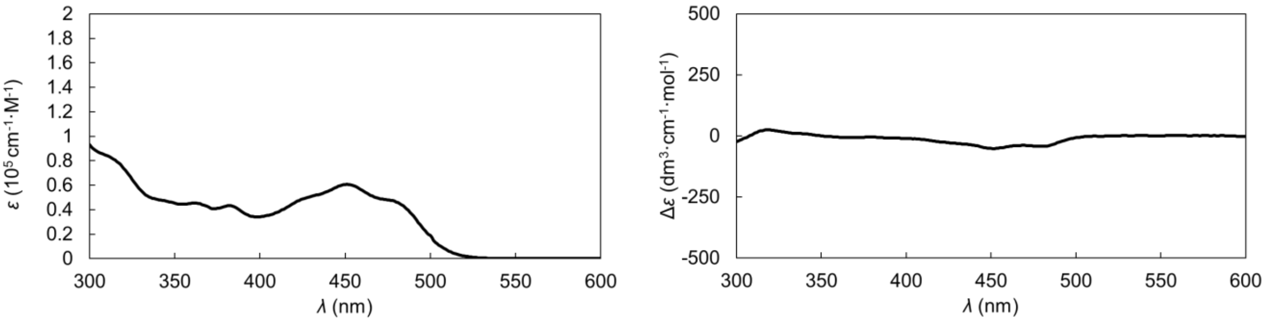


Figure S55. UV-vis absorption and CD spectra of [(*R*)-(**1c**)$_2$Zn][OTf]$_2$ in $CHCl_3$ ([(*R*)-(**1c**)$_2$Zn][OTf]$_2$ = 5.0 μM).

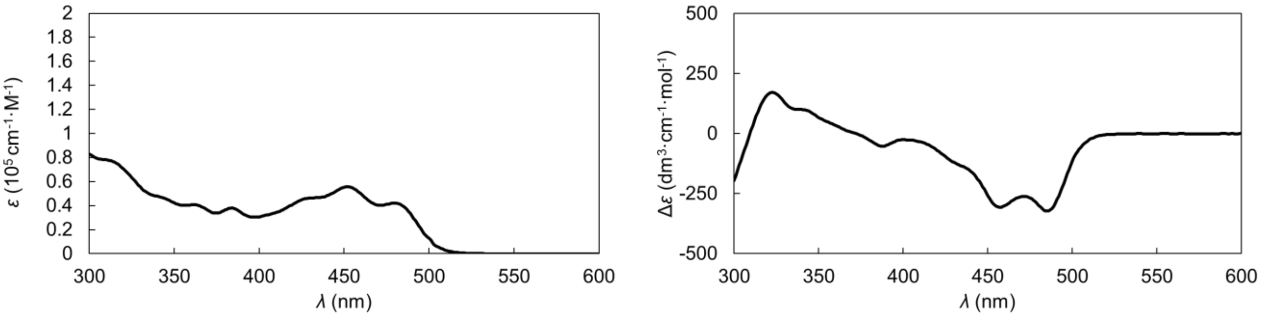


Figure S56. UV-vis absorption and CD spectra of [(*R*)-(**1c**)$_2$Zn][OTf]$_2$ in $CCl_4$ ([(*R*)-(**1c**)$_2$Zn][OTf]$_2$ = 5.0 μM).

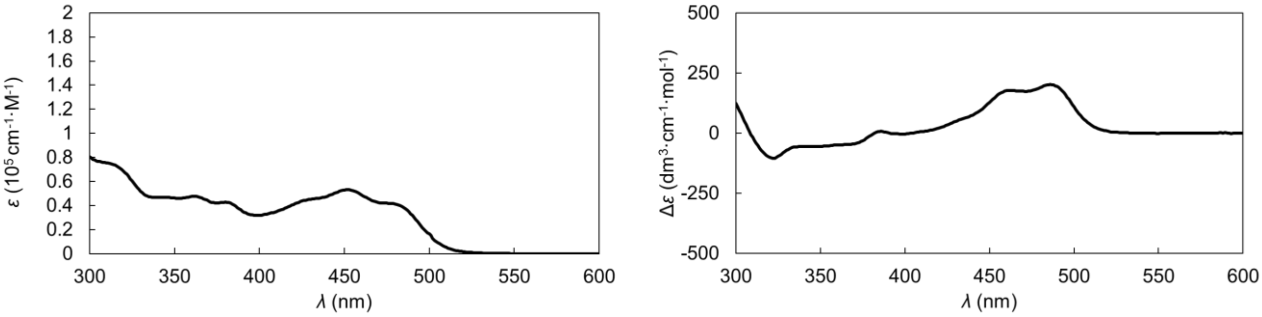


Figure S57. UV-vis absorption and CD spectra of [(*R*)-(**1c**)$_2$Zn][OTf]$_2$ in $C_2H_4Cl_2$ ([(*R*)-(**1c**)$_2$Zn][OTf]$_2$ = 5.0 μM).

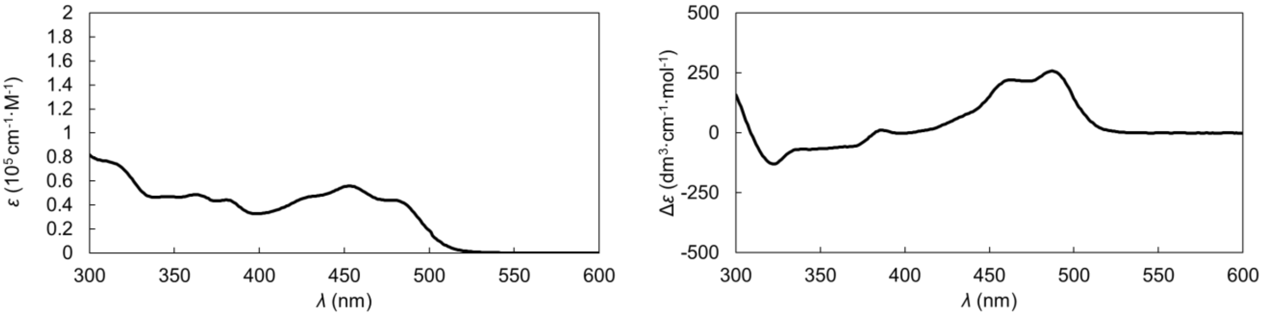


Figure S58. UV-vis absorption and CD spectra of [(*R*)-(**1c**)$_2$Zn][OTf]$_2$ in $C_2H_2Cl_4$ ([(*R*)-(**1c**)$_2$Zn][OTf]$_2$ = 5.0 μM).

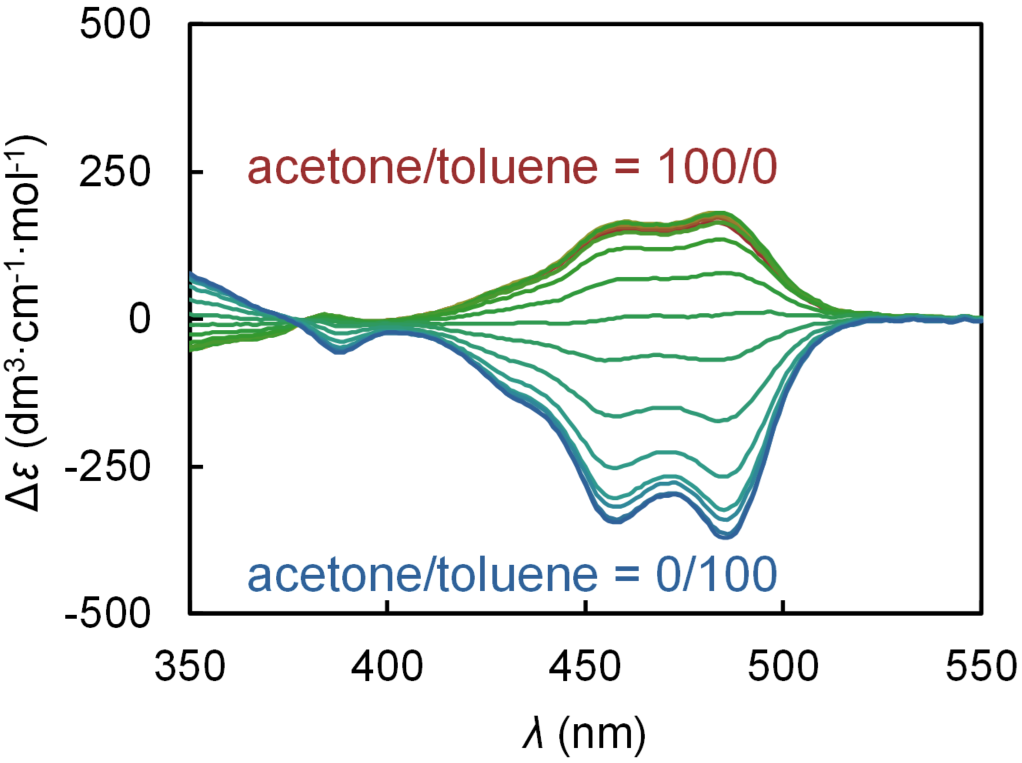


Figure S59. CD spectra of [(*R*)-(**1c**)$_2$Zn][OTf]$_2$ in mixed solvent acetone/toluene ([(*R*)-(**1c**)$_2$Zn][OTf]$_2$ = 5.0 μM).

**Temperature and concentration dependence of [(*R*)-(1c)$_2$Zn][OTf]$_2$**

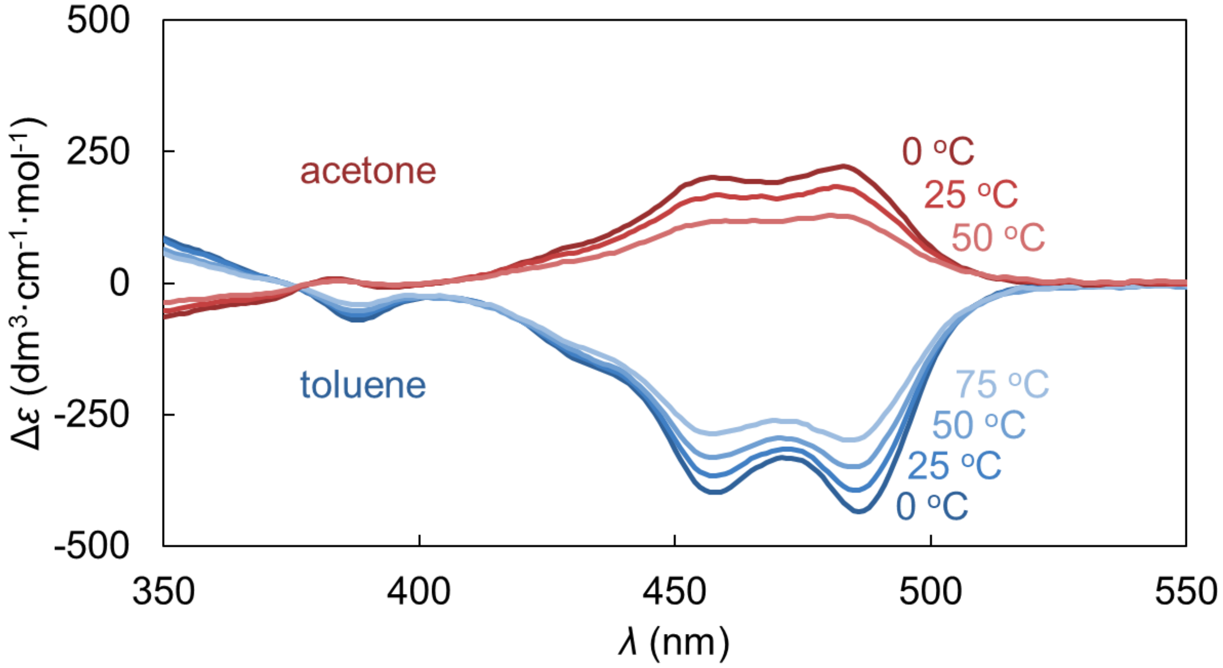


Figure S60. Temperature dependence of CD spectra of [(*R*)-(**1c**)$_2$Zn][OTf]$_2$ in acetone and toluene ([(*R*)-(**1c**)$_2$Zn][OTf]$_2$ = 5.0 μM).

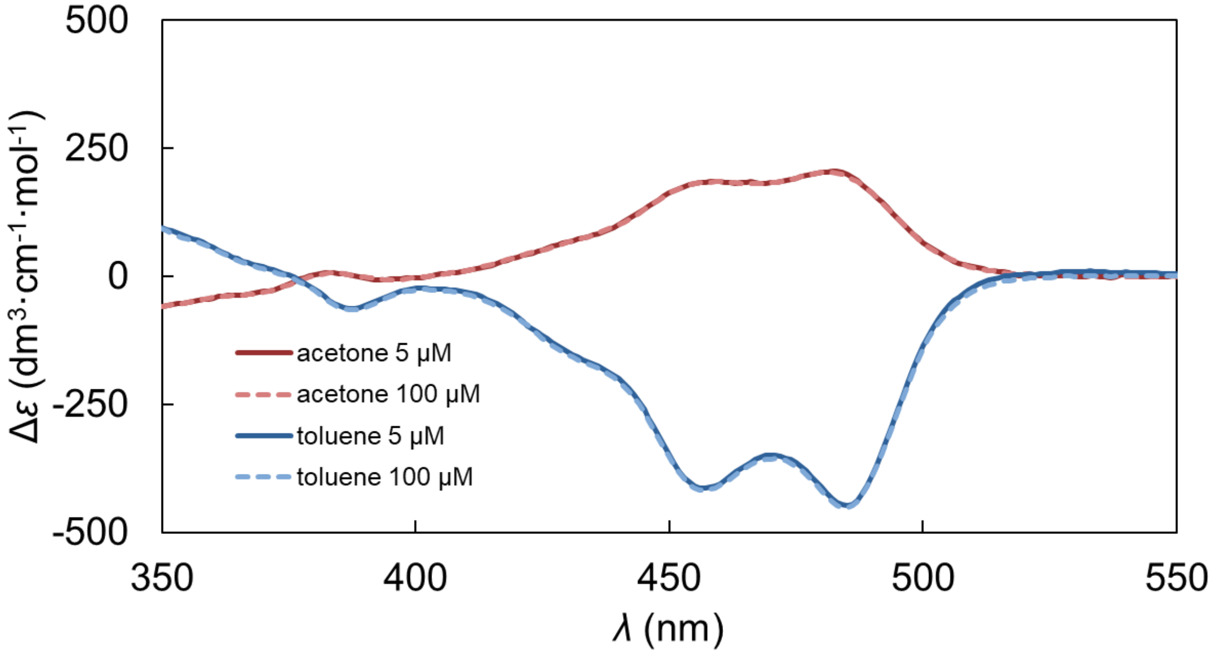


Figure S61. Concentration dependence of CD spectra of [(*R*)-(**1c**)$_2$Zn][OTf]$_2$ in acetone and toluene ([(*R*)-(**1c**)$_2$Zn][OTf]$_2$ = 5.0 μM and 100 μM).

## 9. Time dependence of CD changes from (*M*)-helicity to (*P*)-helicity

The *P*/*M* helicity inversion was expected to be a pseudo first-order reaction via open forms, and the reversal kinetics of double-helices were calculated by the time dependence of the CD signal (Scheme S4, Figure S62, Figure S63). [(*R*)-(**1c**)$_2$Zn][OTf]$_2$ was dissolved in toluene, and the [(*R*)-(**1c**)$_2$Zn][OTf]$_2$ biased (*M*)-helicity was obtained by evaporation from the toluene solution. The solid [(*R*)-(**1c**)$_2$Zn][OTf]$_2$ biased (*M*)-helicity showed a negative Cotton effect at 485 nm when measured immediately after dissolution in acetone, but this gradually changed to a positive Cotton effect over time. The half-life of the inversion was 224 s at 20 $^{O}$C, 69 s at 30 $^{O}$C and 29 s at 40 $^{O}$C. The CD signal reached an equilibrium state after about 5-30 min. These changes in the Cotton effect were accompanied by a time-dependent change in the diastereomeric ratio, and the activation energy $\Delta G^{\ddagger}_{MP}$ for the inversion from (*M*) to (*P*)-helicity could be estimated ($\Delta G^{\ddagger}_{MP}$ = 20.6 kcal/mol at 293 K, 20.7 kcal/mol at 303 K and $\Delta G^{\ddagger}_{MP}$ = 20.8 kcal/mol at 313 K). Additionally, the activation entropy and enthalpy were calculated from the Eyring-Polanyi plot [$\Delta S^{\ddagger}_{MP}$ = −9.8 cal/(mol·K), $\Delta H^{\ddagger}_{MP}$ = 17.7 kcal/mol, Figure S64]. The transition states could be two intermediate structures between the (*M*)- or (*P*)-double-helical forms and the open forms. The conformational restriction of the side chains during the rotation of the L-shaped units should have resulted in $\Delta H^{\ddagger}_{MP} > 0$ and $\Delta S^{\ddagger}_{MP} < 0$, and negative activation entropy ($\Delta S^{\ddagger}_{MP} < 0$) might imply that the mechanism of helicity inversion was not a pass through the dissociated state of the strands with a high degree of freedom.

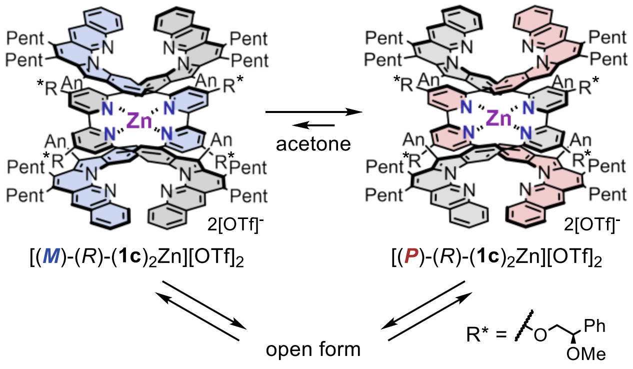


Scheme S4. *P*/*M* helicity inversion of [(*R*)-(**1c**)$_2$Zn][OTf]$_2$ in acetone.

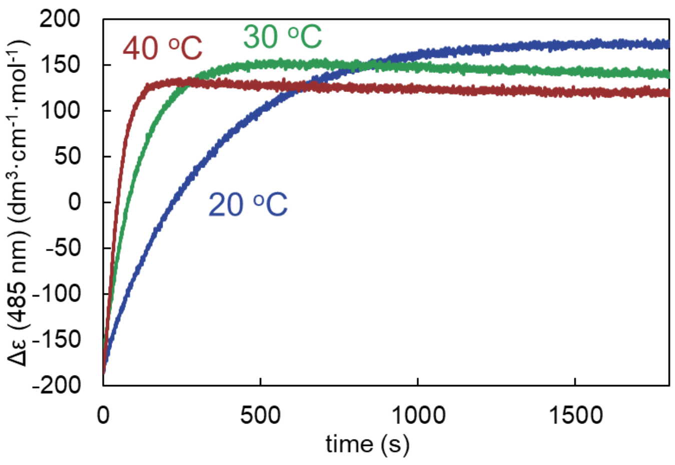


Figure S62. CD changes at 485 nm of the solutions [(*R*)-(**1c**)$_2$Zn][OTf]$_2$ (5.0 μM) after dissolving the toluene-solvated solid [(*R*)-(**1c**)$_2$Zn][OTf]$_2$ at a) 20 °C, b) 30 °C and c) 40 °C.

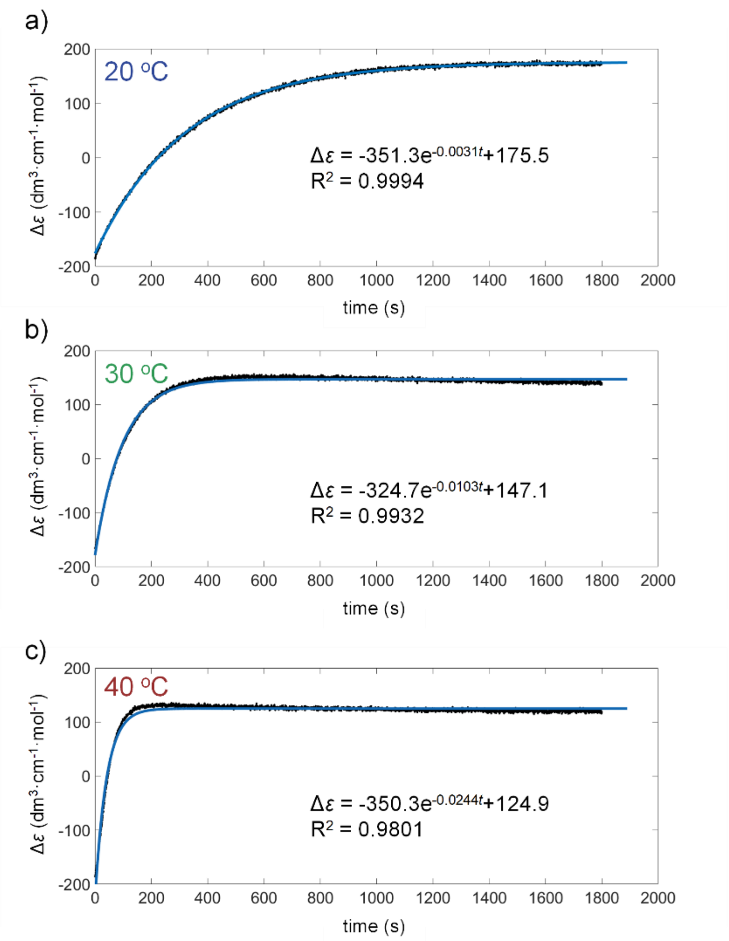


Figure S63. CD changes (black) and fitting (blue) as pseude-first-order process of helix inversion of [(*R*)-(**1c**)$_2$Zn][OTf]$_2$ at a) 20 °C, b) 30 °C and c) 40 °C.

**Kinetics for *M*/*P* switching from (*M*)-helicity to (*P*)-helicity**

The kinetic analysis was calculated under the assumption that the existence of the open forms could be ignorable and that the $\Delta\varepsilon$ of the [(*M*)-(*R*)-(**1c**)$_2$Zn][OTf]$_2$ and [(*P*)-(*R*)-(**1c**)$_2$Zn][OTf]$_2$ were equal.

In a pseudo-first order reaction, the concentrations of (*M*)-helicity and (*P*)-helicity are proportional to $e^{-kt}$, and the CD intensity is proportional to their respective concentrations. The molar circular dichroism $\Delta\varepsilon$ is expressed by *eq.* S7 where $\Delta\varepsilon_0$ is the initial molar circular dichroism and $\Delta\varepsilon_\infty$ is the molar circular dichroism when the equilibrium reaches the steady state. $k$ is the sum of the forward, $k_{MP}$, and backward, $k_{PM}$, pseudo-first order rate constants for the exchange process (*eq.* S8). $k$ was calculated from the fittings shown in Figure S63 and *eq.* S7 ($k_{20} = 3.1\times10^{-3}$ s$^{-1}$, $k_{30} = 1.0\times10^{-2}$ s$^{-1}$, $k_{20} = 2.4\times10^{-2}$ s$^{-1}$), and half-life $\tau_{1/2}$ was calculated from *eq.* S11 (Table S12). $k_{MP}X_M$ and $k_{PM}X_P$ are equal at equilibrium (*eq.* S9) and $X_M$ and $X_P$ were determined by analyzing the integral ratio of the $^1$H NMR signal in acetone-$d_6$ (Table S11) due to the exchange rate constant $k_{MP}$ and $k_{PM}$ can be determined (Table S12). Furthermore, $\Delta G^‡_{MP}$ was calculated from Eyring−Polanyi equation (*eq.* S10, Table S12), and $\Delta H^‡_{MP}$ and $\Delta S^‡_{MP}$ were calculated from Eyring−Polanyi plot ($\Delta H^‡_{MP}$ = 17.7 kcal·mol$^{-1}$, $\Delta S^‡_{MP}$ = -9.8 cal·mol$^{-1}$·K$^{-1}$, Figure S64).

$$\Delta\varepsilon = (\Delta\varepsilon_0 - \Delta\varepsilon_\infty)e^{-kt} + \Delta\varepsilon_\infty \qquad (eq. S7)$$

$$k = k_{\mathrm{MP}} + k_{\mathrm{PM}} \qquad (eq. S8)$$

$$k_{\mathrm{MP}}X_{\mathrm{M}} = k_{\mathrm{PM}}X_{\mathrm{P}} \qquad (eq. S9)$$

$$\Delta G^‡ = -RT\ln\frac{hk}{k_{\mathrm{B}}T} \qquad (eq. S10)$$

$$\tau_{1/2} = \frac{\ln 2}{k} \qquad (eq. S11)$$

Table S11. Mole fractions of [(*M*)-(*R*)-(**1c**)$_2$Zn][OTf]$_2$ and [(*P*)-(*R*)-(**1c**)$_2$Zn][OTf]$_2$ in the equilibrium state determined by analyzing the integral ratio of the $^1$H NMR signal in acetone-$d_6$.

| *Temp.* | $X_M$ | $X_P$ |
|---|---|---|
| 20 °C | 77 | 23 |
| 30 °C | 79 | 21 |
| 40 °C | 80 | 20 |

Table S12. Kinetic parameters of helix inversion from [(*M*)-(*R*)-(**1c**)$_2$Zn][OTf]$_2$ to [(*P*)-(*R*)-(**1c**)$_2$Zn][OTf]$_2$.

| *Temp.* | $k_{MP}$ (s$^{-1}$) | $k_{PM}$ (s$^{-1}$) | $\Delta G^‡$ (kcal·mol$^{-1}$) | $\tau_{1/2}$ (s) |
|---|---|---|---|---|
| 20 °C | $2.5\times10^{-3}$ | $6.2\times10^{-4}$ | 20.6 | 224 |
| 30 °C | $7.9\times10^{-3}$ | $2.1\times10^{-3}$ | 20.7 | 69 |
| 40 °C | $1.8\times10^{-2}$ | $5.5\times10^{-3}$ | 20.8 | 29 |

$R$ = 1.987 (cal·K$^{-1}$·mol$^{-1}$)

$k_B = 3.30\times10^{-24}$ (cal·K$^{-1}$)

$h = 1.58\times10^{-34}$ (cal·s)

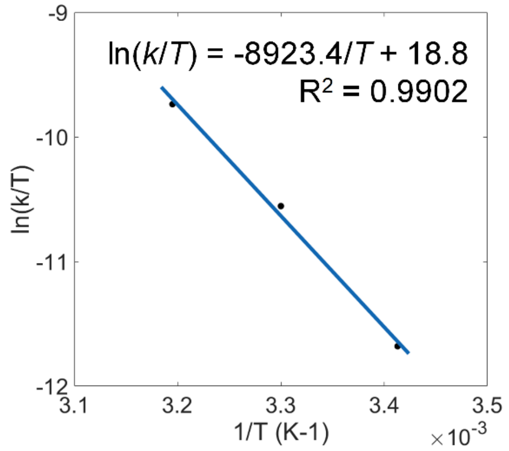


Figure S64. Eyring-Polanyi plot of helix inversion from [(*M*)-(*R*)-(**1c**)$_2$Zn][OTf]$_2$ to [(*P*)-(*R*)-(**1c**)$_2$Zn][OTf]$_2$.

## 10. Theoretical calculations

**General.** All DFT and TD-DFT calculations were carried out with ORCA version 5.0.4.[S8-11] Geometry optimization and TD-DFT calculations were carried out at the revPBE0-D3(BJ)[S12,13]/def2-SVP[S14] (for C, H, N, O), def2-TZVP[S14] (for Zn) level of theory with the resolution of RICJOSX approximation using the def2/J[S15] auxiliary basis set. TD-DFT calculations were performed using the Tamm-Dancoff approximation.

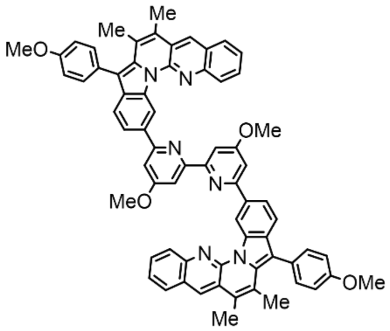


**1d**

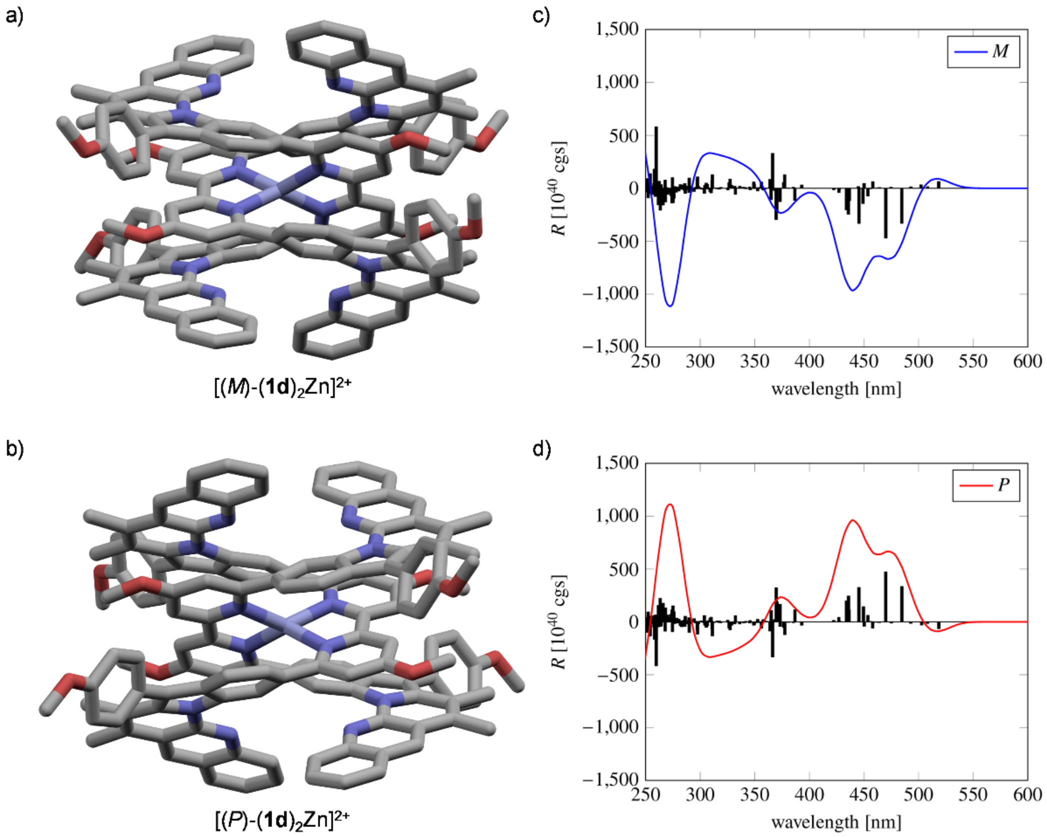


Figure S65. a) Optimized structures and b) CD spectra of [(*M*)-(**1d**)$_2$Zn]$^{2+}$, and c) Optimized structures and d) CD spectra of [(*P*)-(**1d**)$_2$Zn]$^{2+}$ calculated by TD-DFT/TDA (Orca 5.0.4, revPBE0-D3(BJ)/def2-SVP for C, H, N, O, def2-TZVP for Zn).

## 11. NMR study ($^{1}$H NMR, 2D COSY and ROESY) for [(*R*)-(1c)$_2$Zn][OTf]$_2$

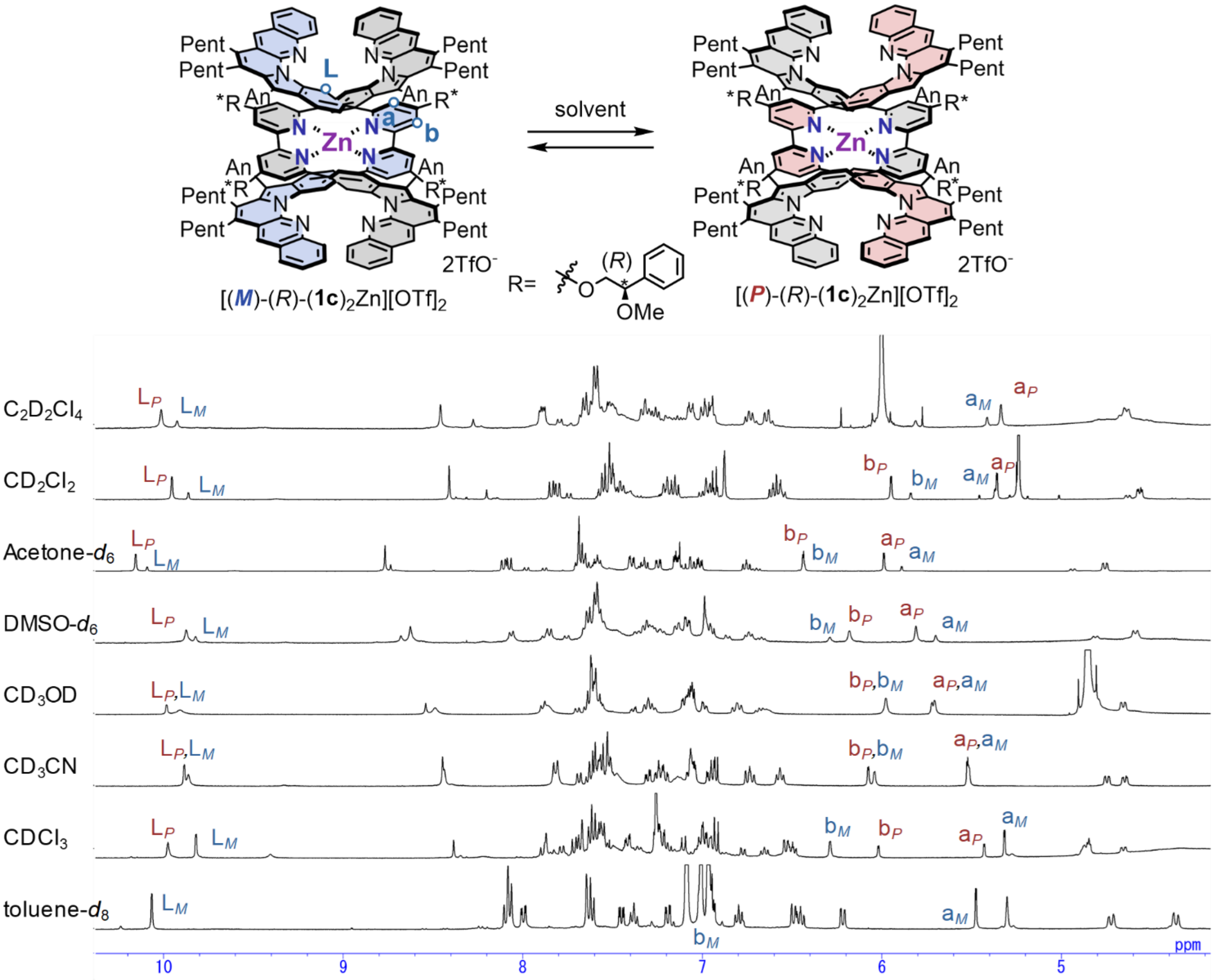


Figure S66. $^{1}$H NMR spectra of [(*R*)-(**1c**)$_2$Zn][OTf]$_2$ (1.0 mM, 400 MHz, 298 K).

**Calculation of equilibrium ratio of *M*/*P*/open forms for [(*R*)-(1c)$_2$Zn][OTf]$_2$**

The ratio of (*M*)-double-helical form to (*P*)-double-helical form to open forms of [(*R*)-(**1c**)$_2$Zn][OTf]$_2$ was calculated from $^1$H NMR using $C_2H_2Cl_4$ as the internal standard.

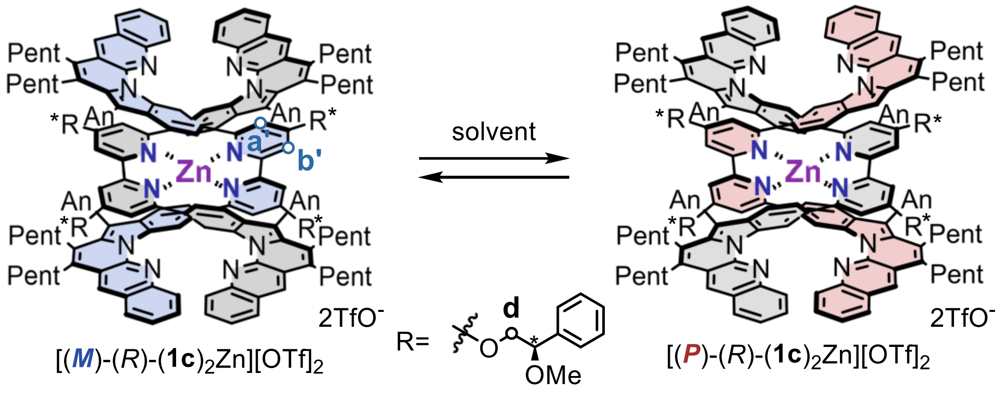


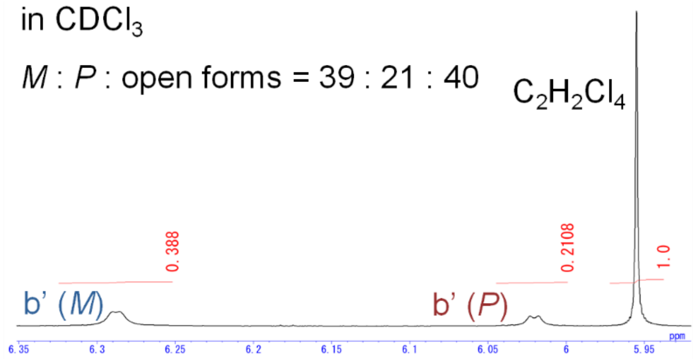


Figure S67. $^1$H NMR spectra of [(*R*)-(**1c**)$_2$Zn][OTf]$_2$ with the addition of $C_2H_2Cl_4$ ([(*R*)-(**1c**)$_2$Zn][OTf]$_2$=1.0 mM, [$C_2H_2Cl_4$] =2.0 mM, $CDCl_3$ 400 MHz, 298 K).

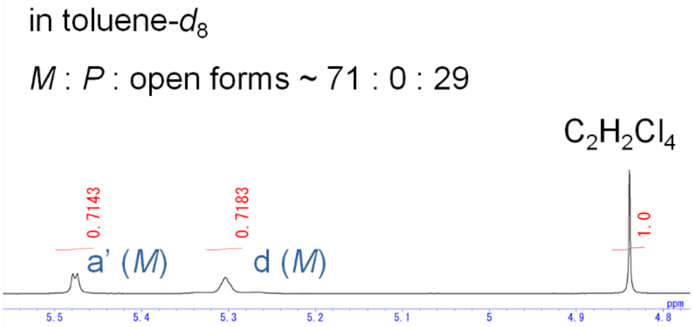


Figure S68. $^1$H NMR spectra of [(*R*)-(**1c**)$_2$Zn][OTf]$_2$ with the addition of $C_2H_2Cl_4$ ([(*R*)-(**1c**)$_2$Zn][OTf]$_2$=1.0 mM, [$C_2H_2Cl_4$] =2.0 mM, acetone-$d_6$ 400 MHz, 298 K).

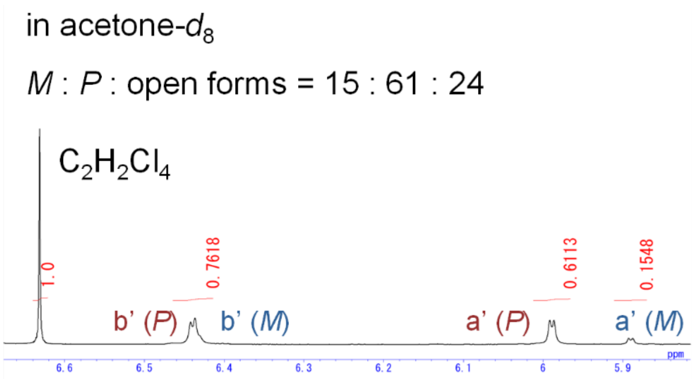


Figure S69. $^{1}$H NMR spectra of [($R$)-(**1c**)$_2$Zn][OTf]$_2$ with the addition of $C_2H_2Cl_4$ ([($R$)-(**1c**)$_2$Zn][OTf]$_2$=1.0 mM, [$C_2H_2Cl_4$] =2.0 mM, toluene-$d_8$ 400 MHz, 298 K).

**Concentration dependence of [(*R*)-(1c)$_2$Zn][OTf]$_2$**

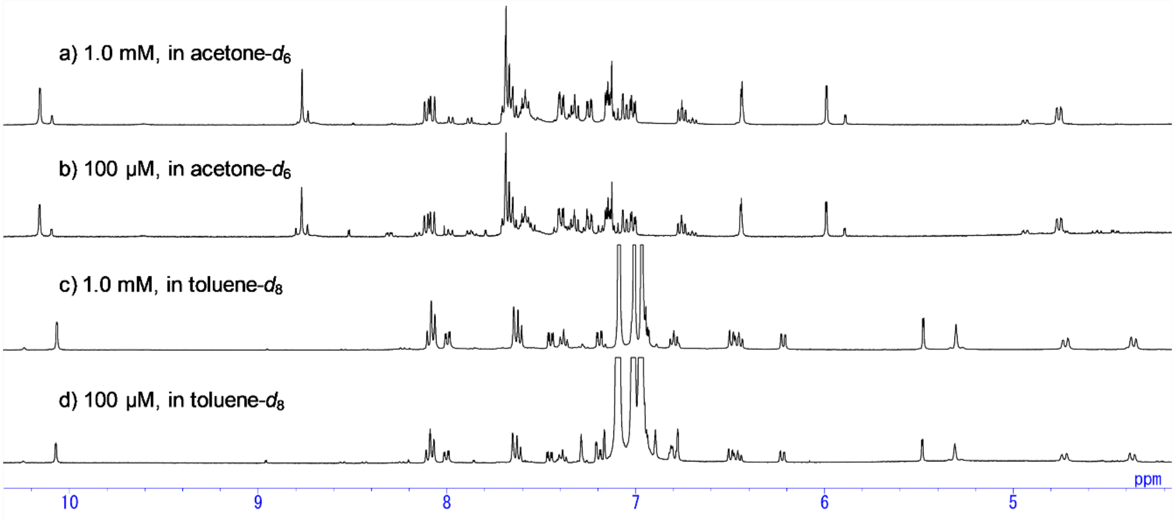


Figure S70. $^{1}$H NMR spectra (400 MHz, 298 K, 5.2-10.4 ppm) of [(*R*)-(**1c**)$_2$Zn][OTf]$_2$ a) in acetone-$d_6$ (1.0 mM), b) in acetone-$d_6$ (100 μM), c) in toluene-$d_8$ (1.0 mM) and d) in toluene-$d_8$ (100 μM).

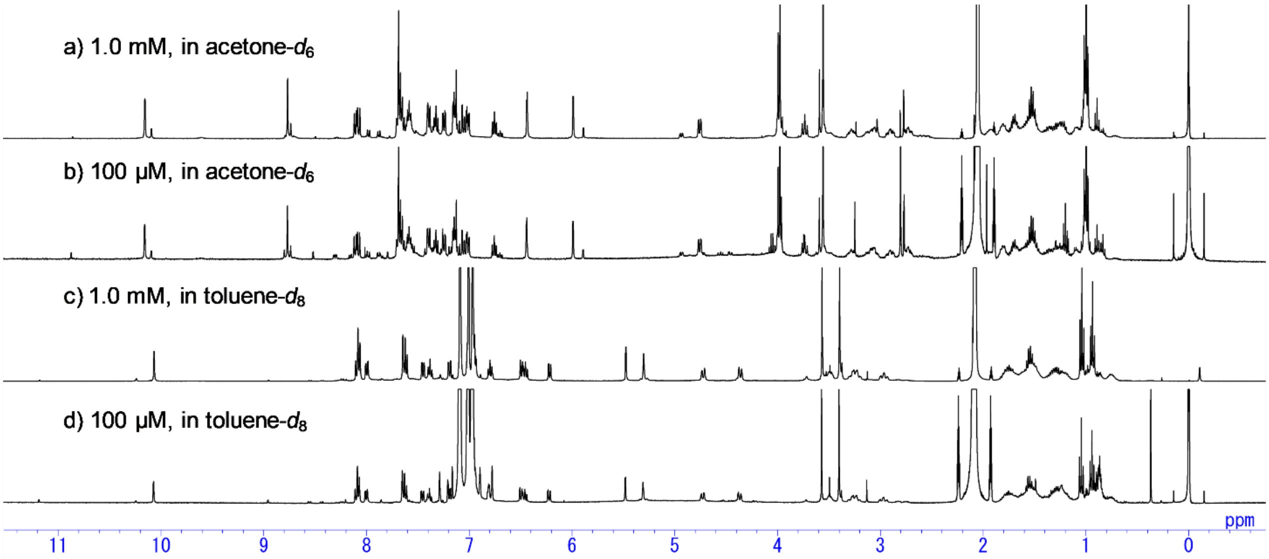


Figure S71. $^{1}$H NMR spectra (400 MHz, 298 K) of [(*R*)-(**1c**)$_2$Zn][OTf]$_2$ a) in acetone-$d_6$ (1.0 mM), b) in acetone-$d_6$ (100 μM), c) in toluene-$d_8$ (1.0 mM) and d) in toluene-$d_8$ (100 μM).

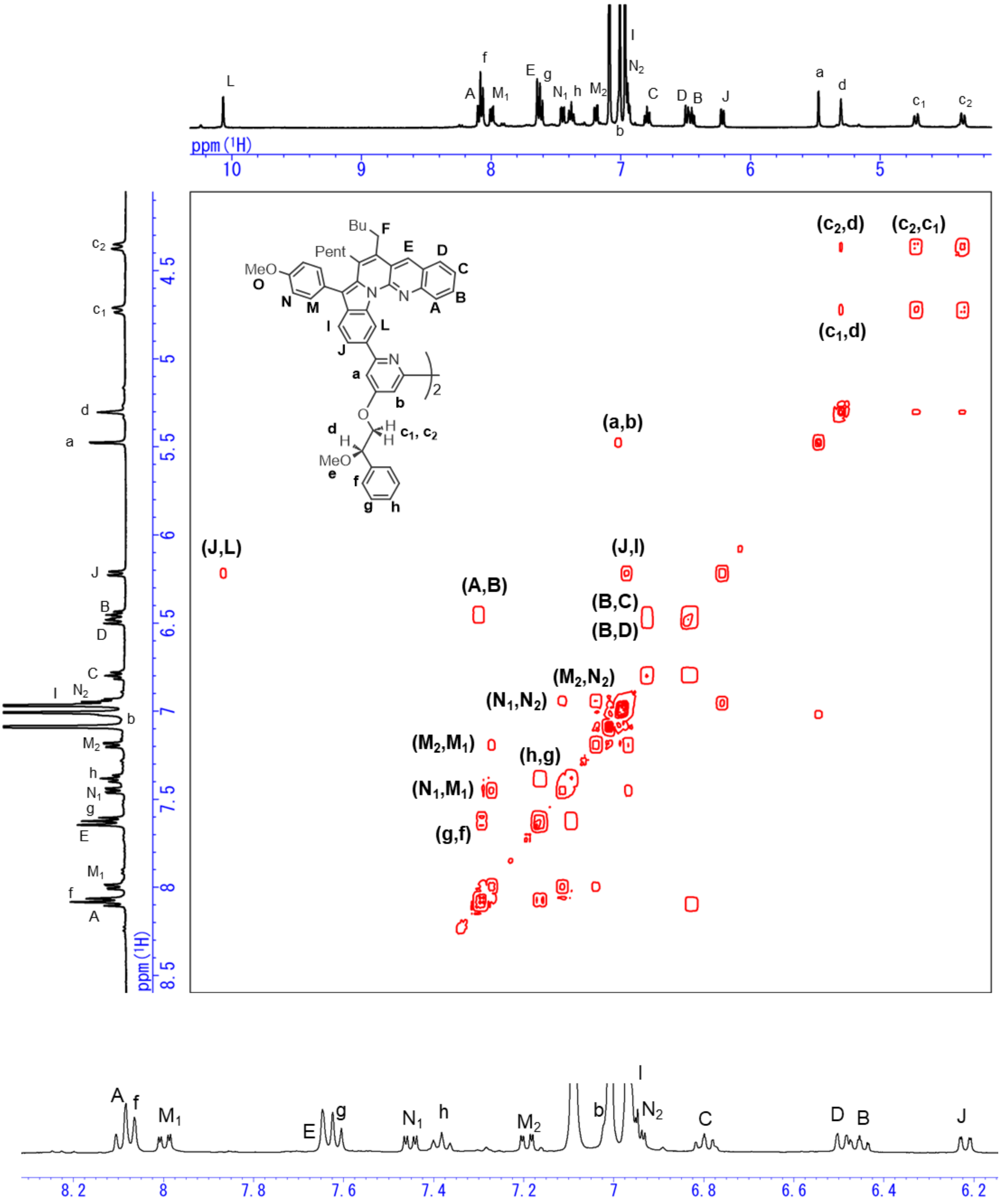

Figure S72. 2D COSY spectrum of [(*R*)-(**1c**)$_2$Zn][OTf]$_2$ (toluene-$d_8$, 298 K).

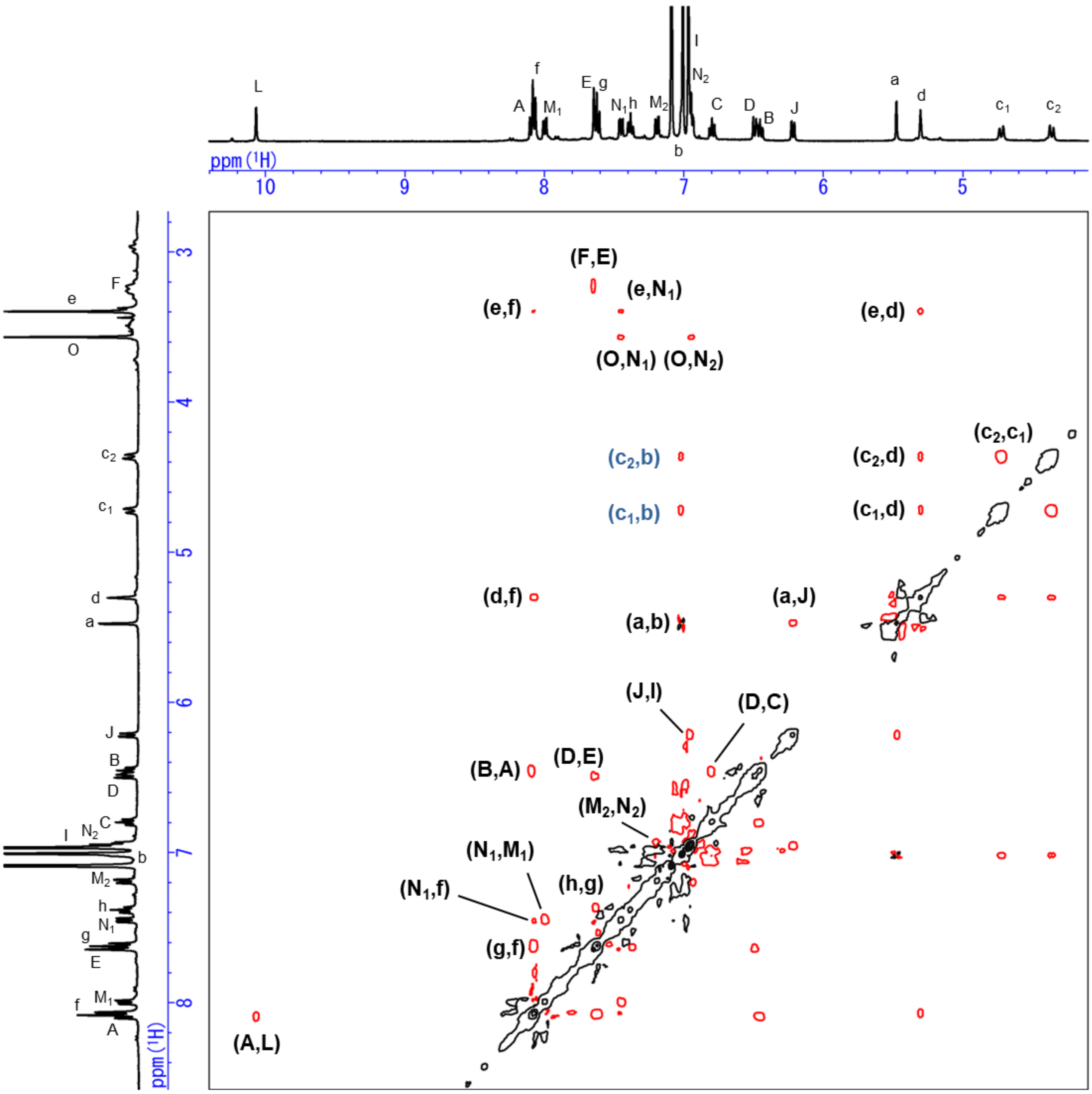

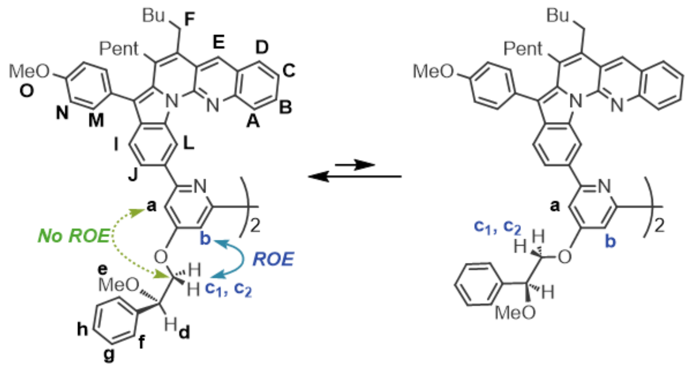


Figure S73. 2D ROESY spectrum of [(*R*)-(**1c**)$_2$Zn][OTf]$_2$ (toluene-$d_8$, 298 K, mixing time =0.2 s).

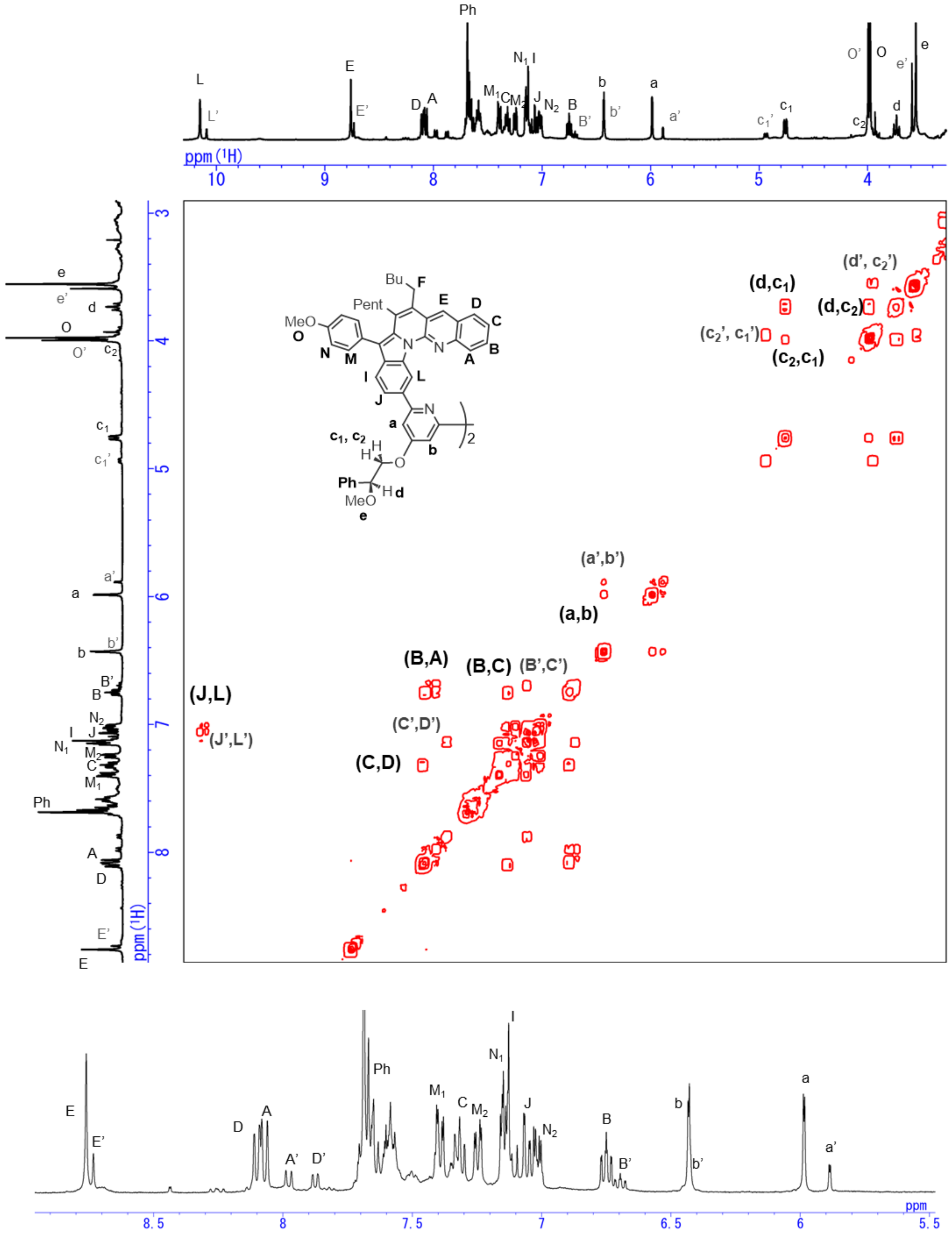

Figure S74. 2D COSY spectrum of [(*R*)-(**1c**)$_2$Zn][OTf]$_2$ (acetone-$d_6$, 298 K).

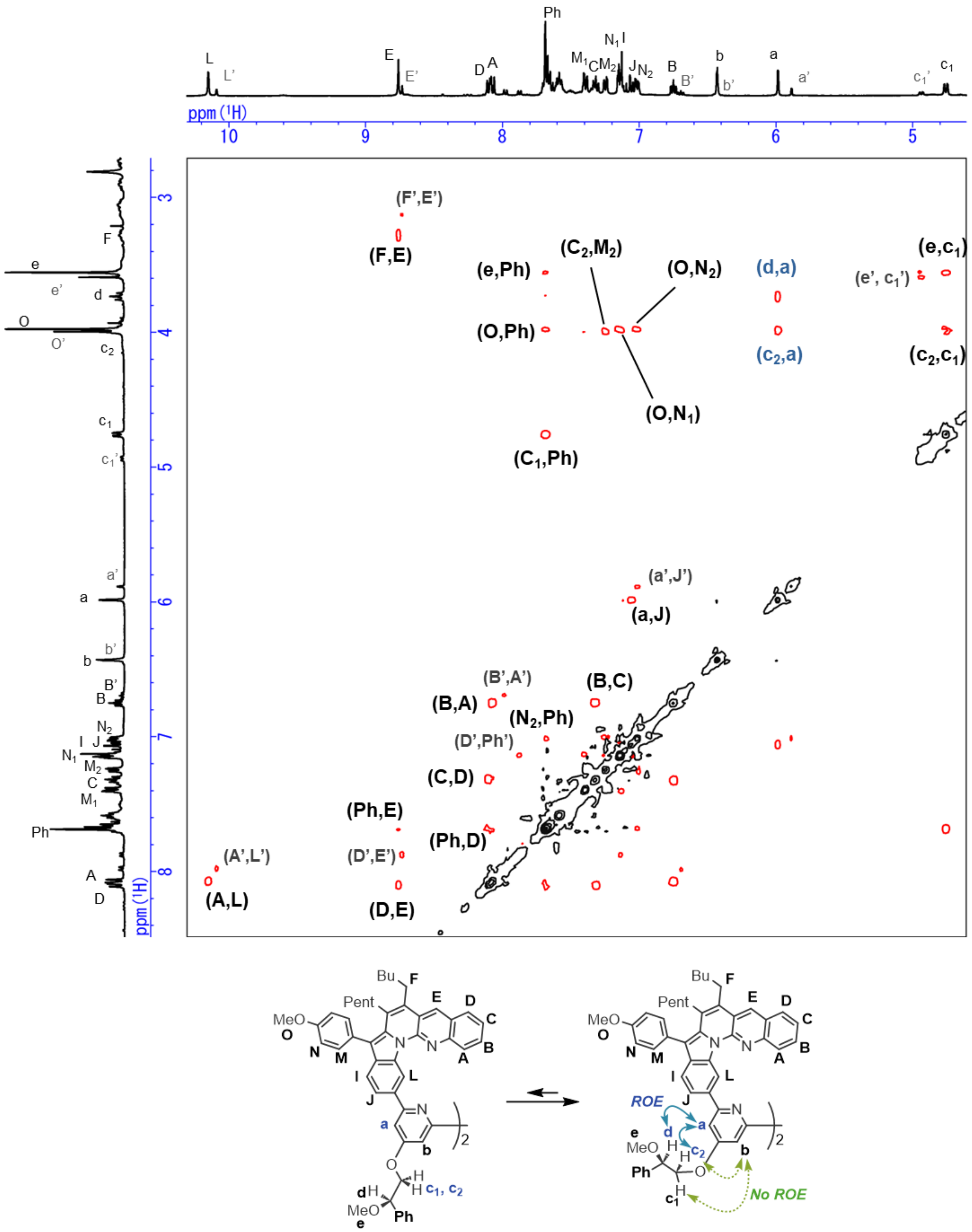


Figure S75. 2D ROESY spectrum of [(*R*)-(**1c**)$_2$Zn][OTf]$_2$ (acetone-*d*$_6$, 298 K, mixing time =0.2 s).

## 12. van't Hoff plot for the equilibrium between (*M*) and (*P*)-double-helical forms of [(*R*)-(1c)$_2$Zn][OTf]$_2$

[(*R*)-(**1c**)$_2$Zn][OTf]$_2$ : To an NMR tube was charged with **1c** (1.5 mg, 1 μmol) and $CDCl_3$ (0.50 mL) was added $Zn(OTf)_2$ in acetone-*d*$_6$ (50 mM, 10 μL, 0.5 μmol, 0.5 eq.) and MS3A (10 mg). After the NMR tube was stirred for 1 min, the $^1H$ NMR spectrum of the resulting mixture was recorded, which confirmed the formation of [(*R*)-(**1c**)$_2$Zn][OTf]$_2$. The solution was concentrated in *vacuo*. and subsequently acetone-*d*$_6$ (0.50 mL) were added to the NMR tube. The solution of [(*R*)-(**1c**)$_2$Zn][OTf]$_2$ (1.0 mM) was subjected to VT NMR analyses.

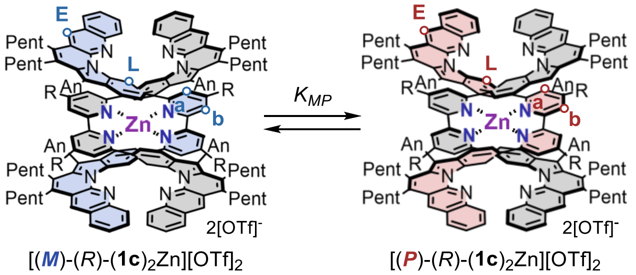


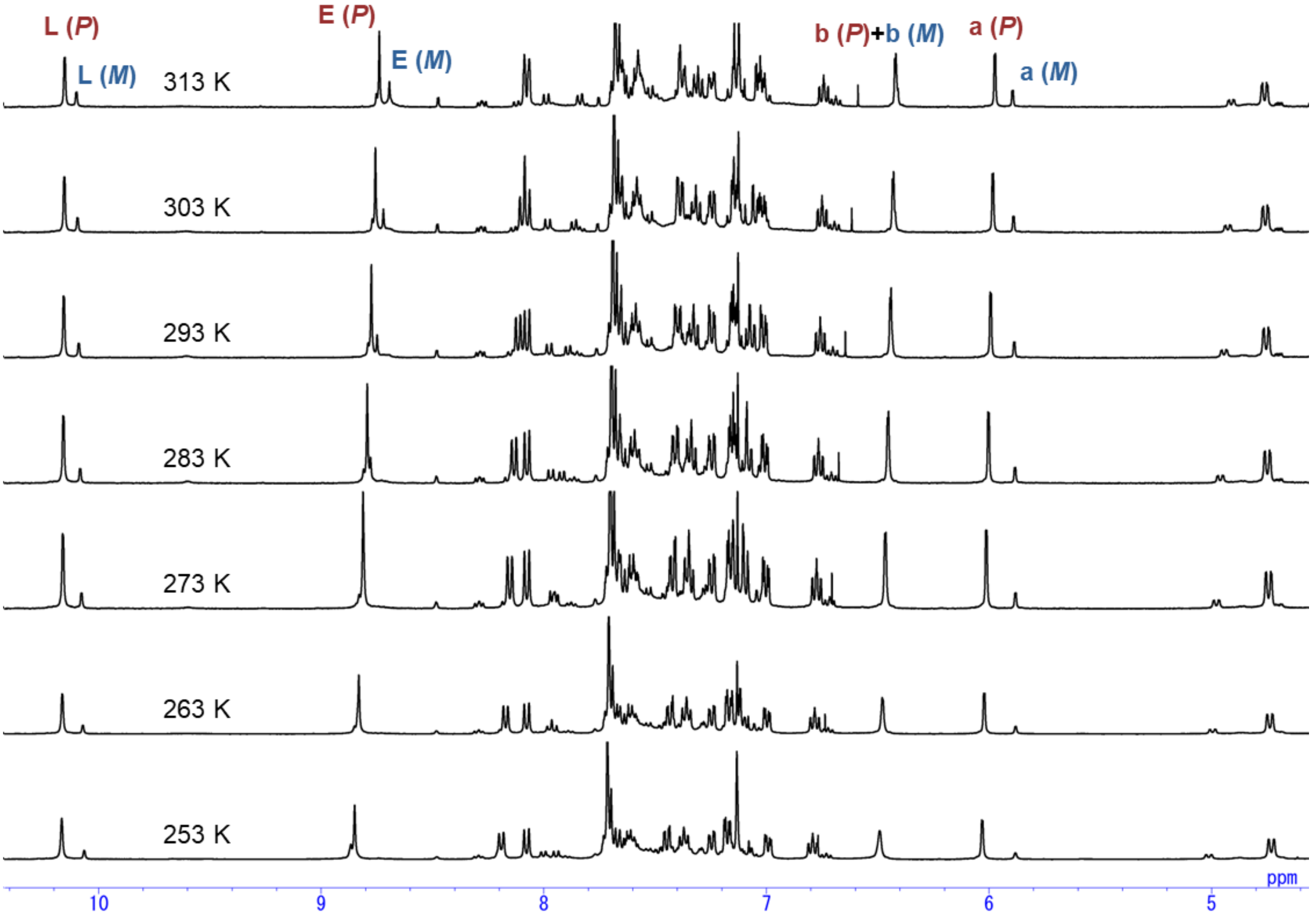


Figure S76. Comparison of representative region of the $^1H$ NMR spectra (400 MHz) of [(*R*)-(**1c**)$_2$Zn][OTf]$_2$ in acetone-*d*$_6$ (1.0 μM) at various temperatures.

Table S13. Relative ratio of [(*M*)-(*R*)-(**1c**)$_2$Zn][OTf]$_2$ and [(*P*)-(*R*)-(**1c**)$_2$Zn][OTf]$_2$, determined by the integrations of $^1$H NMR spectra at various temperatures in $CDCl_3$.

| | integration | | ratio | |
|---|---|---|---|---|
| *temp.* (K) | *P* | *M* | *P* | *M* |
| 313 | 1 | 0.30 | 0.77 | 0.23 |
| 303 | 1 | 0.27 | 0.79 | 0.21 |
| 293 | 1 | 0.24 | 0.80 | 0.20 |
| 283 | 1 | 0.23 | 0.81 | 0.19 |
| 273 | 1 | 0.21 | 0.83 | 0.17 |
| 263 | 1 | 0.20 | 0.83 | 0.17 |
| 253 | 1 | 0.18 | 0.85 | 0.15 |

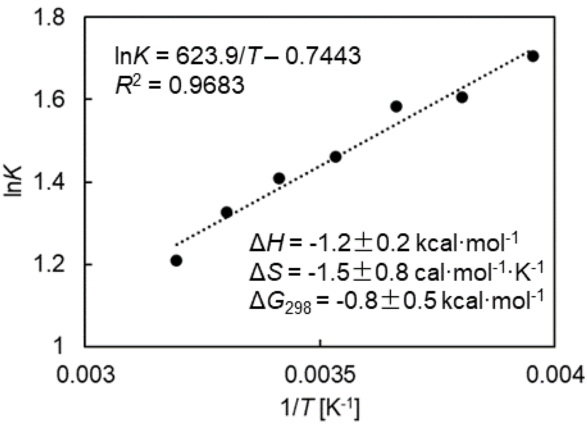


Figure S77. The van't Hoff plot for the equilibriums of [(*M*)-(*R*)-(**1c**)$_2$Zn][OTf]$_2$ and [(*P*)-(*R*)-(**1c**)$_2$Zn][OTf]$_2$ of [(**1c**)$_2$Zn][OTf]$_2$ in $CDCl_3$.

# 13. Chiral amplification via heteroleptic monometallofoldamer [(1b)(1c)Zn][OTf]$_2$

**Preparation of a mixture [(1b)$_2$Zn][OTf]$_2$/[(1b)(1c)Zn][OTf]$_2$/[(1c)$_2$Zn][OTf]$_2$ by mixing strands 1b and 1c in a 1/1 ratio**

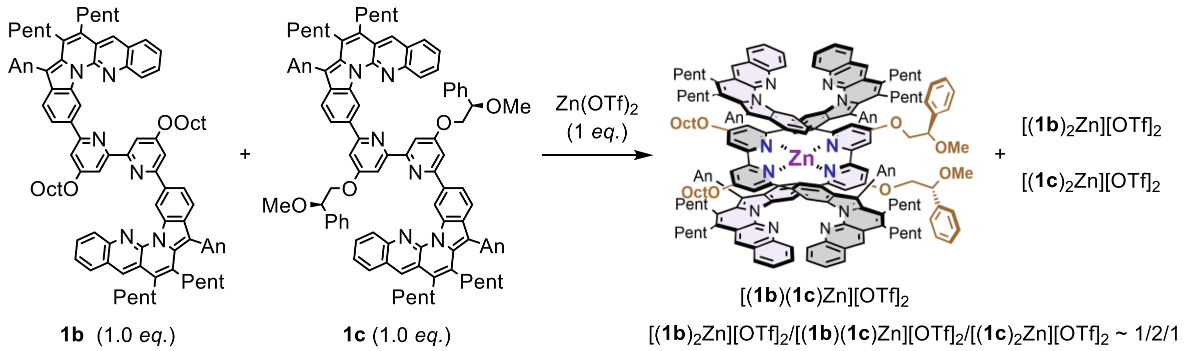


**Mixture of [(1b)$_2$Zn][OTf]$_2$/[(1b)(1c)Zn][OTf]$_2$/[(1c)$_2$Zn][OTf]$_2$**: To an NMR tube was charged with **1b** in $CHCl_3$ (2.0 mM, 0.25 mL, 0.5 μmol) and **1c** in $CHCl_3$ (2.0 mM, 0.25 mL, 0.5 μmol) was added Zn(OTf)$_2$ in acetone (50 mM, 10 μL, 0.5 μmol) and MS3A (10 mg). After the NMR tube was stirred for 1 min, the solution was concentrated to give a mixture of [(**1b**)$_2$Zn][OTf]$_2$, [(**1b**)(**1b'**)Zn][OTf]$_2$ and [(**1c**)$_2$Zn][OTf]$_2$.

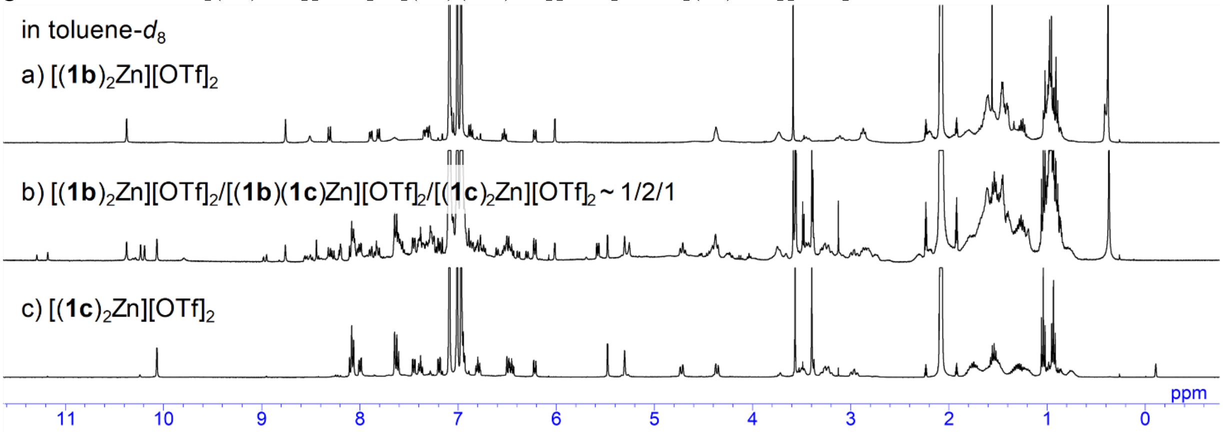


Figure S78. $^1$H NMR of a) [(**1b**)$_2$Zn][OTf]$_2$, b) [(**1b**)$_2$Zn][OTf]$_2$/[(**1b**)(**1b'**)Zn][OTf]$_2$/[(**1c**)$_2$Zn][OTf]$_2$~1/2/1, c) [(**1c**)$_2$Zn][OTf]$_2$ (toluene-$d_8$, 400 MHz, 298 K).

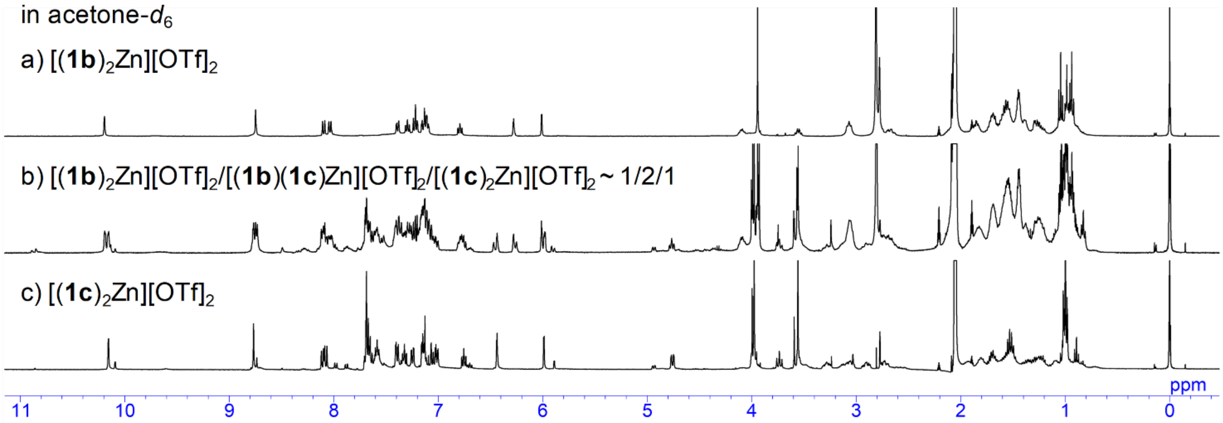


Figure S79. $^1$H NMR of a) [(**1b**)$_2$Zn][OTf]$_2$, b) [(**1b**)$_2$Zn][OTf]$_2$/[(**1b**)(**1b'**)Zn][OTf]$_2$/[(**1c**)$_2$Zn][OTf]$_2$~1/2/1, c) [(**1c**)$_2$Zn][OTf]$_2$ (acetone-$d_6$, 400 MHz, 298 K).

**Preparation of a mixture [(1b)$_2$Zn][OTf]$_2$/[(1b)(1c)Zn][OTf]$_2$/[(1c)$_2$Zn][OTf]$_2$ by mixing strands 1b and 1c in various ratio**

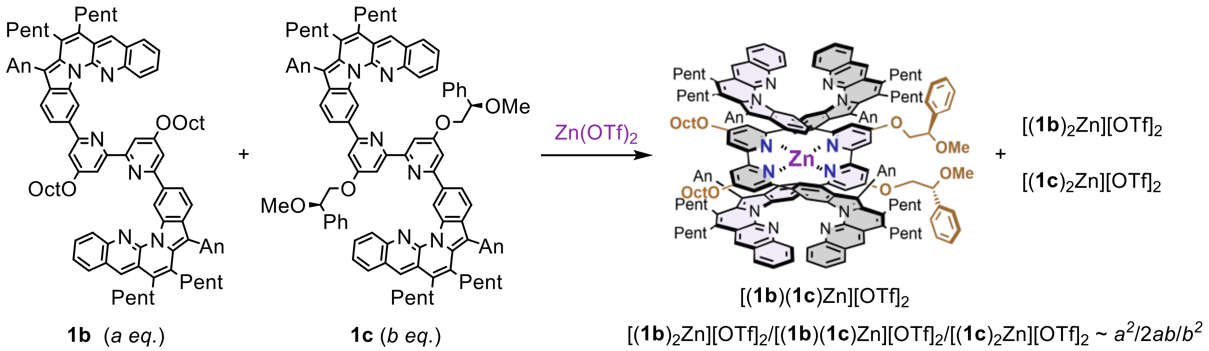


**1b** (*a eq.*) **1c** (*b eq.*) [(**1b**)$_2$Zn][OTf]$_2$/[(**1b**)(**1c**)Zn][OTf]$_2$/[(**1c**)$_2$Zn][OTf]$_2$ ~ $a^2/2ab/b^2$

**Mixtures [(1b)$_2$Zn][OTf]$_2$/[(1b)(1c)Zn][OTf]$_2$/[(1c)$_2$Zn][OTf]$_2$**: To an NMR tube was charged with various ratio of **1b** in $CHCl_3$ (2.0 mM, *A* mL) and **1c** in $CHCl_3$ (2.0 mM, *B* mL) as shown in Table S14 was added $Zn(OTf)_2$ in acetone (50 mM, 10 µL, 0.5 µmol) and MS3A (10 mg). After the NMR tube was stirred for 1 min, the solution was concentrated to give a mixture of [(**1b**)$_2$Zn][OTf]$_2$, [(**1b**)(**1b'**)Zn][OTf]$_2$ and [(**1c**)$_2$Zn][OTf]$_2$.

Table S14. Conditions for the complexation to a mixture [(**1b**)$_2$Zn][OTf]$_2$/[(**1b**)(**1c**)Zn][OTf]$_2$/[(**1c**)$_2$Zn][OTf]$_2$.

| *Entry* | *A* mL (*a eq.*) | *B* mL (*b eq.*) |
|---|---|---|
| 1 | 0.50 mL (1 *eq.*) | 0 mL (0 *eq.*) |
| 2 | 0.40 mL (0.8 *eq.*) | 0.10 mL (0.2 *eq.*) |
| 3 | 0.30 mL (0.6 *eq.*) | 0.20 mL (0.4 *eq.*) |
| 4 | 0.25 mL (0.5 *eq.*) | 0.25 mL (0.5 *eq.*) |
| 5 | 0.20 mL (0.4 *eq.*) | 0.30 mL (0.6 *eq.*) |
| 6 | 0.10 mL (0.2 *eq.*) | 0.40 mL (0.8 *eq.*) |
| 7 | 0 mL (0 *eq.*) | 0.50 mL (1.0 *eq.*) |

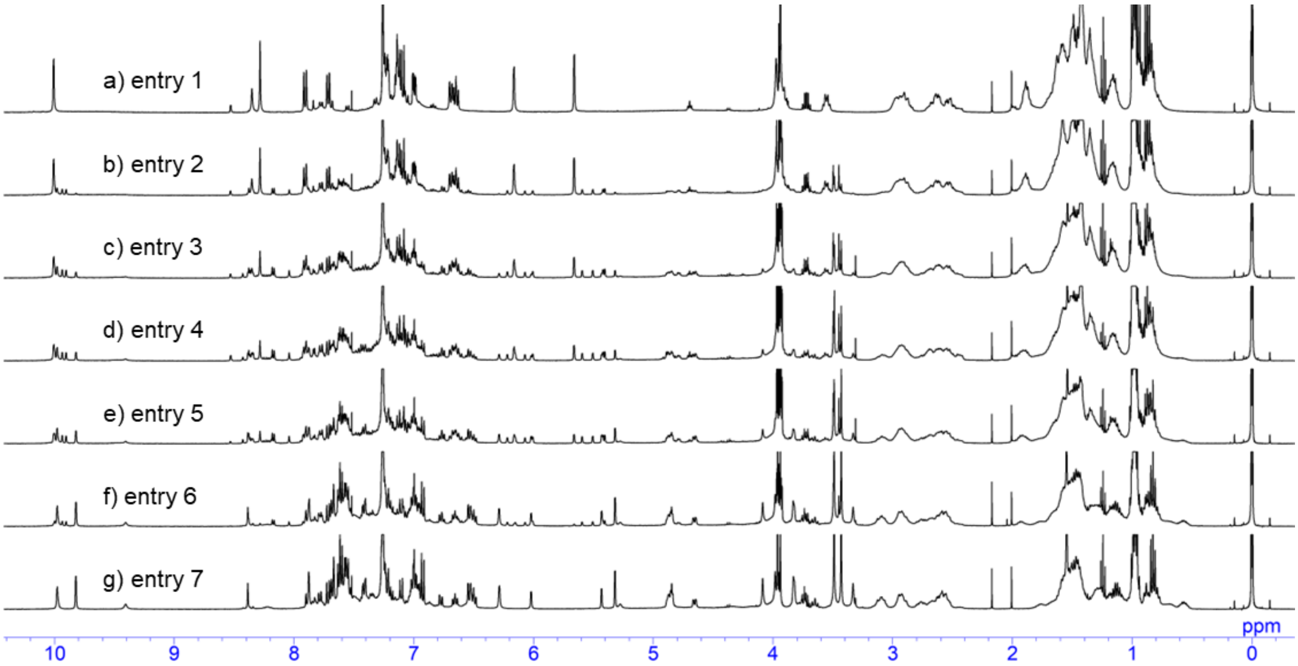


Figure S80. $^{1}$H NMR of a mixture [(**1b**)$_2$Zn][OTf]$_2$/[(**1b**)(**1b'**)Zn][OTf]$_2$/[(**1c**)$_2$Zn][OTf]$_2$ ~ a) 1/0/0, b) 16/8/1, c) 9/12/4, d) 1/2/1, e) 4/12/9, f) 1/8/16 and g) 0/0/1 ($CDCl_3$, 400 MHz, 298 K).

**Preparation and CD measurements of heteroleptic complex [(1b)(1c)Zn][OTf]$_2$ by mixing strand 1c with excess amounts of 1b**

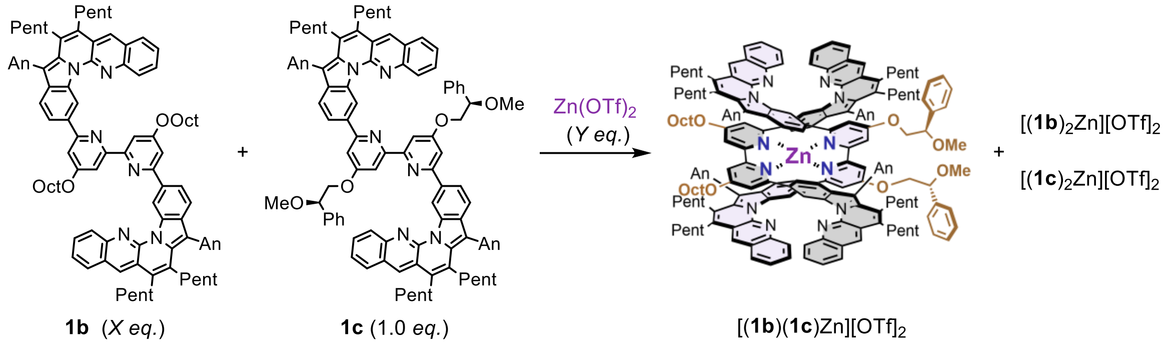


**Mixtures [(1b)$_2$Zn][OTf]$_2$/[(1b)(1c)Zn][OTf]$_2$/[(1c)$_2$Zn][OTf]$_2$**: To a mixtures of **1b** in $CHCl_3$ (1.0 mM, X *eq.*), **1c** in $CHCl_3$ (1.0 mM, 0.10 mL, 0.1 µmol) was added $Zn(OTf)_2$ in acetone (5 mM, Y *eq.*) as shown in Table S15 and MS3A. After the solution was stirred for 1 min, the solutions were concentrated to give a mixture of [(**1b**)$_2$Zn][OTf]$_2$, [(**1b**)(**1b'**)Zn][OTf]$_2$ and [(**1c**)$_2$Zn][OTf]$_2$. The solutions dissolved in toluene or acetone (the concentration of the strand **1c** was 4 µM), and CD measurements were carried out (Figure S81, Figure S82).

Table S15. Conditions for the complexation to [(**1b**)(**1c**)Zn][OTf]$_2$.

| *Entry* | *X* (equivalent of **1b**) | *Y* (equivalent of $Zn(OTf)_2$) |
|---|---|---|
| 1 | 0 *eq.* | 0.5 *eq.* |
| 2 | 1 *eq.* | 1 *eq.* |
| 3 | 2 *eq.* | 1.5 *eq.* |
| 4 | 4 *eq.* | 2.5 *eq.* |
| 5 | 8 *eq.* | 4.5 *eq.* |
| 6 | 12 *eq.* | 6.5 *eq.* |

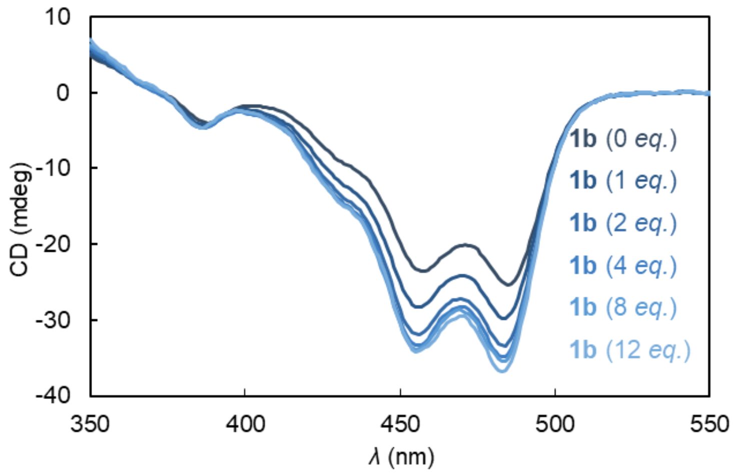


Figure S81. CD spectra of [(*R*)-(**1c**)$_2$Zn][OTf]$_2$ and mixture of [(**1b**)$_2$Zn][OTf]$_2$, [(**1b**)(**1c**)Zn][OTf]$_2$ and [(**1c**)$_2$Zn][OTf]$_2$ in toluene (r.t., the concentration of ligand **1c** was 4 μM).

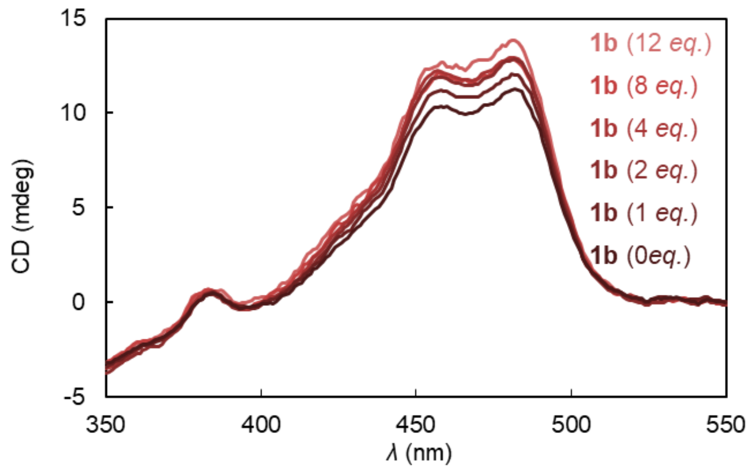


Figure S82. CD spectra of [(*R*)-(**1c**)$_2$Zn][OTf]$_2$ and mixture of [(**1b**)$_2$Zn][OTf]$_2$, [(**1b**)(**1c**)Zn][OTf]$_2$ and [(**1c**)$_2$Zn][OTf]$_2$ in acetone (r.t., the concentration of ligand **1c** was 4 μM).

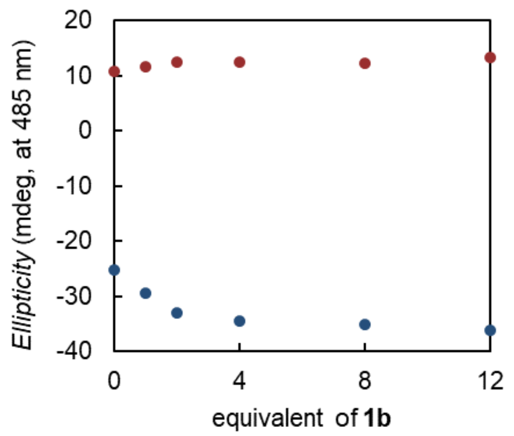


Figure S 83. Amplified Cotton effects upon adding 0-12 equivalents of **1b** and Zn(OTf)$_2$ to 1 equivalent of **1c**.

# 14. $^{1}$H and $^{13}$C NMR spectra of new compounds

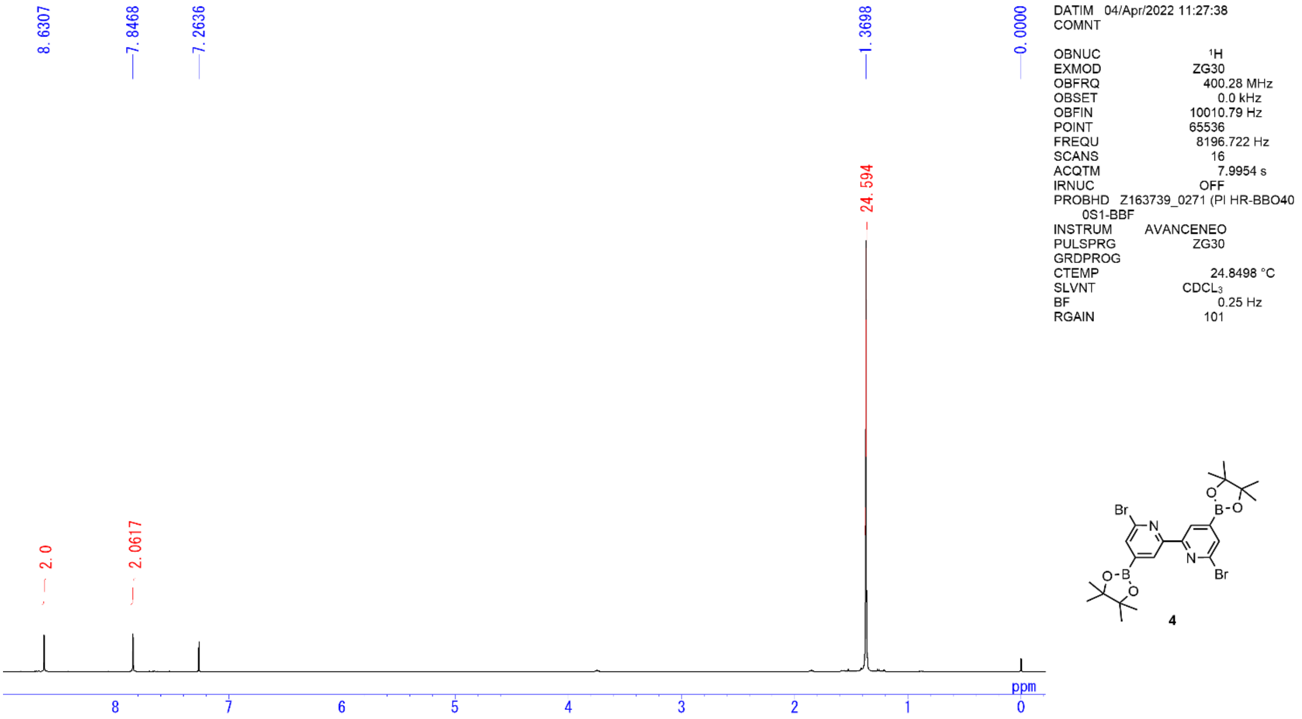


Figure S84. $^{1}$H-NMR spectrum of 4.

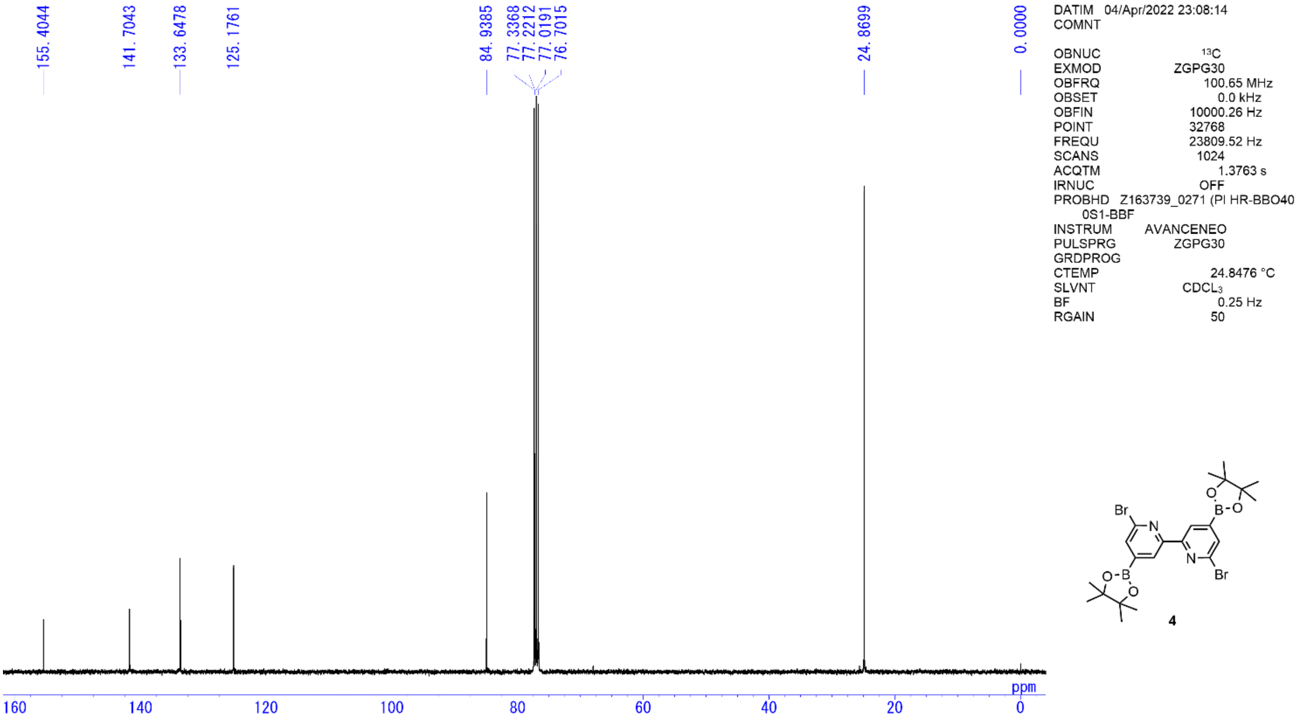


Figure S85. $^{13}$C-NMR spectrum of **4**.

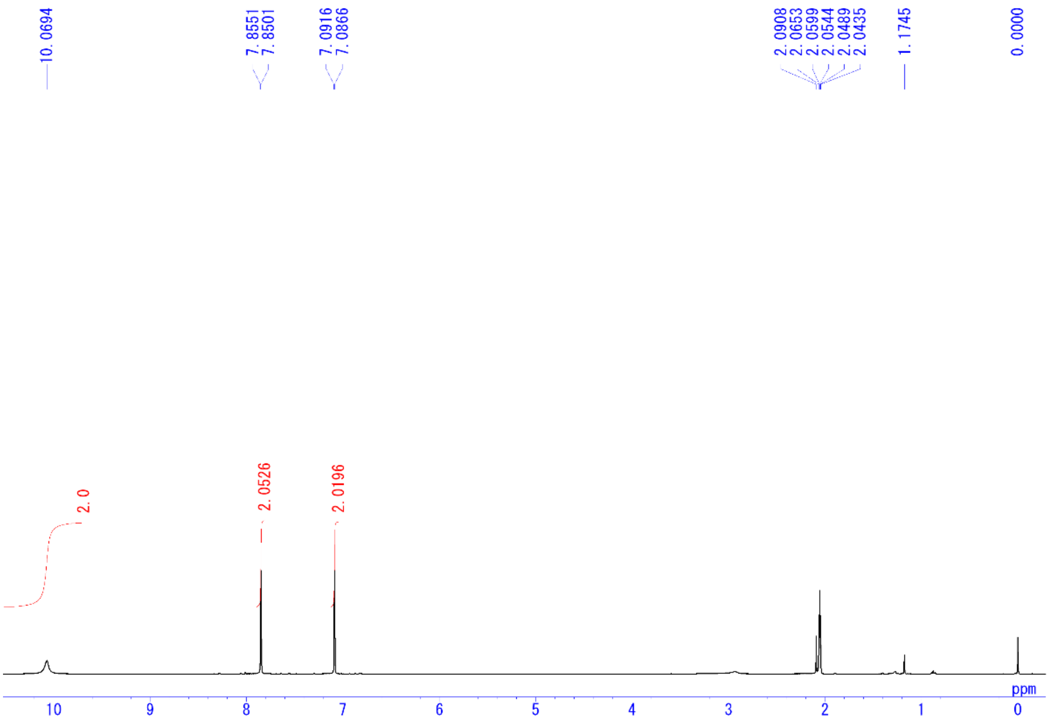


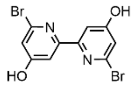


**5**

Figure S86. $^{1}$H-NMR spectrum of **5**.

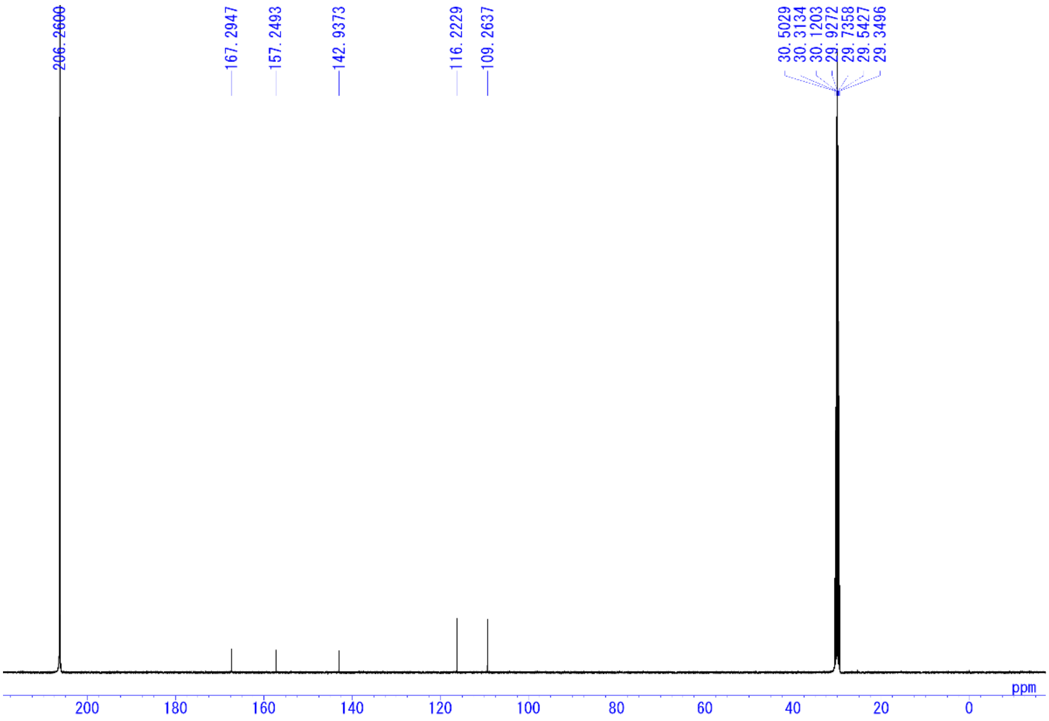


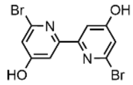


**5**

Figure S87. $^{13}$C-NMR spectrum of **5**.

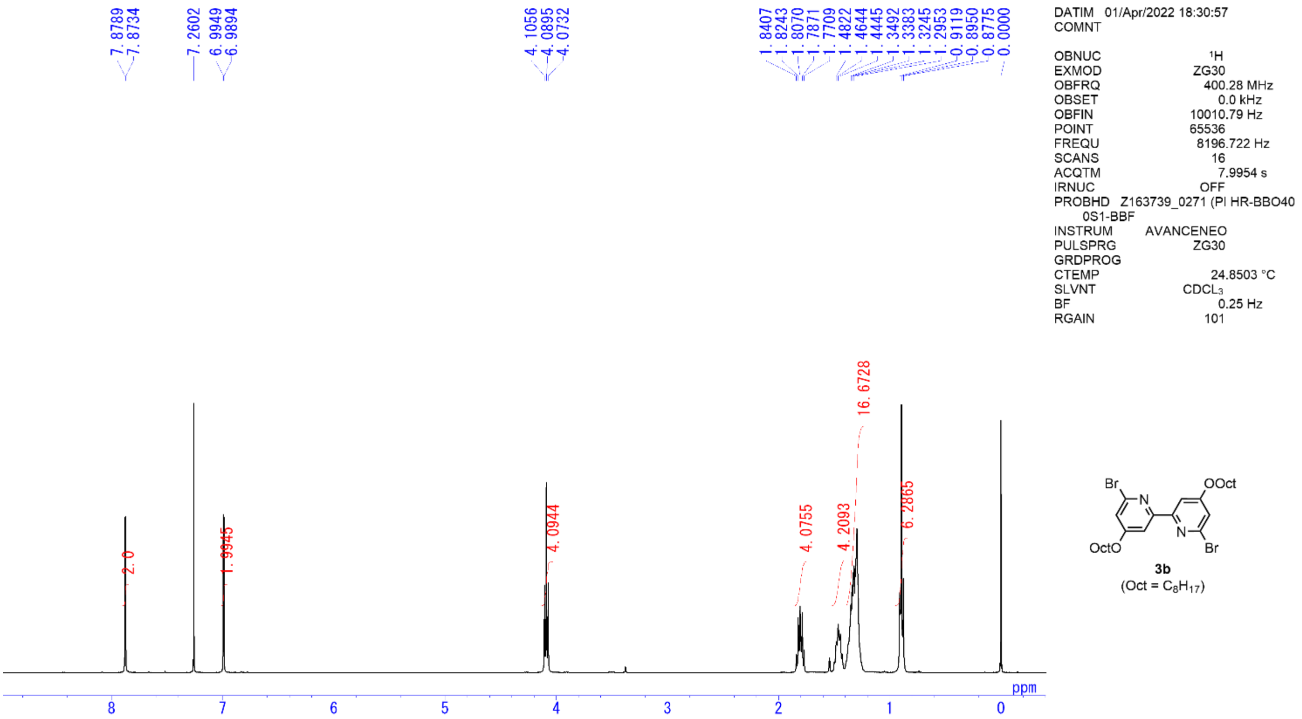


Figure S88. $^{1}$H-NMR spectrum of **3b**.

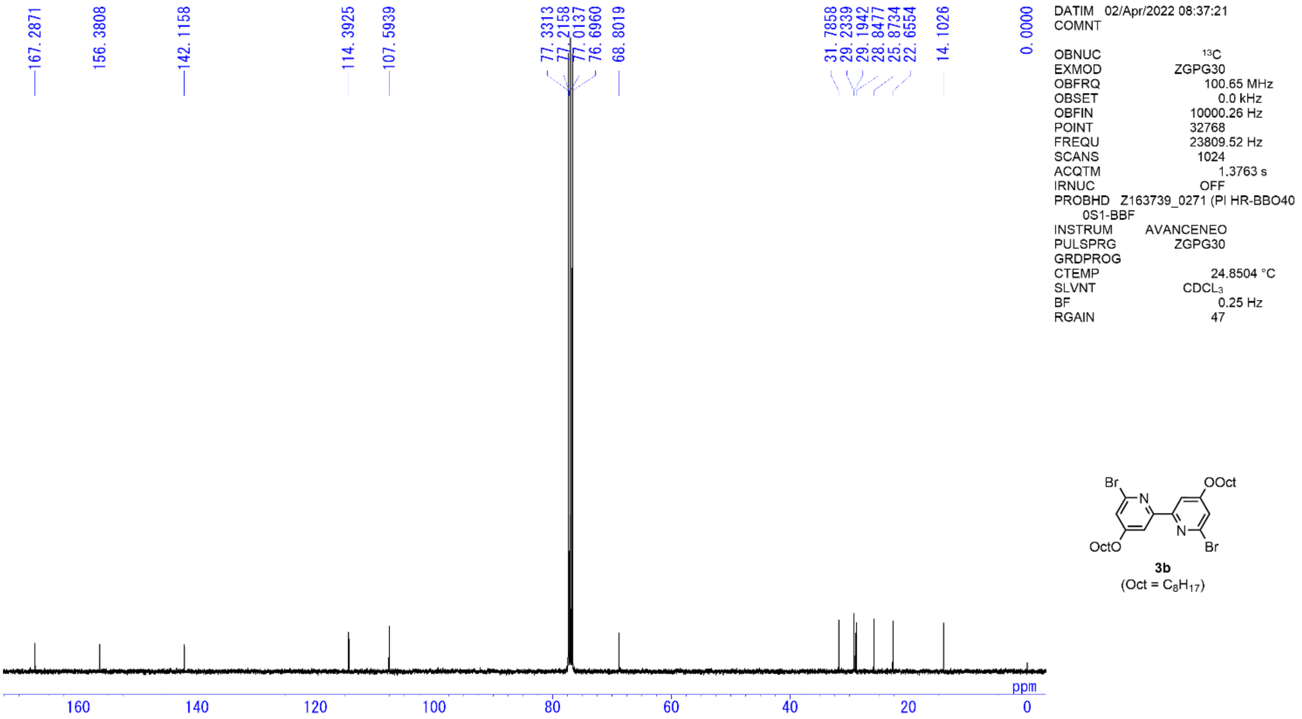


Figure S89. $^{13}$C-NMR spectrum of **3b**.

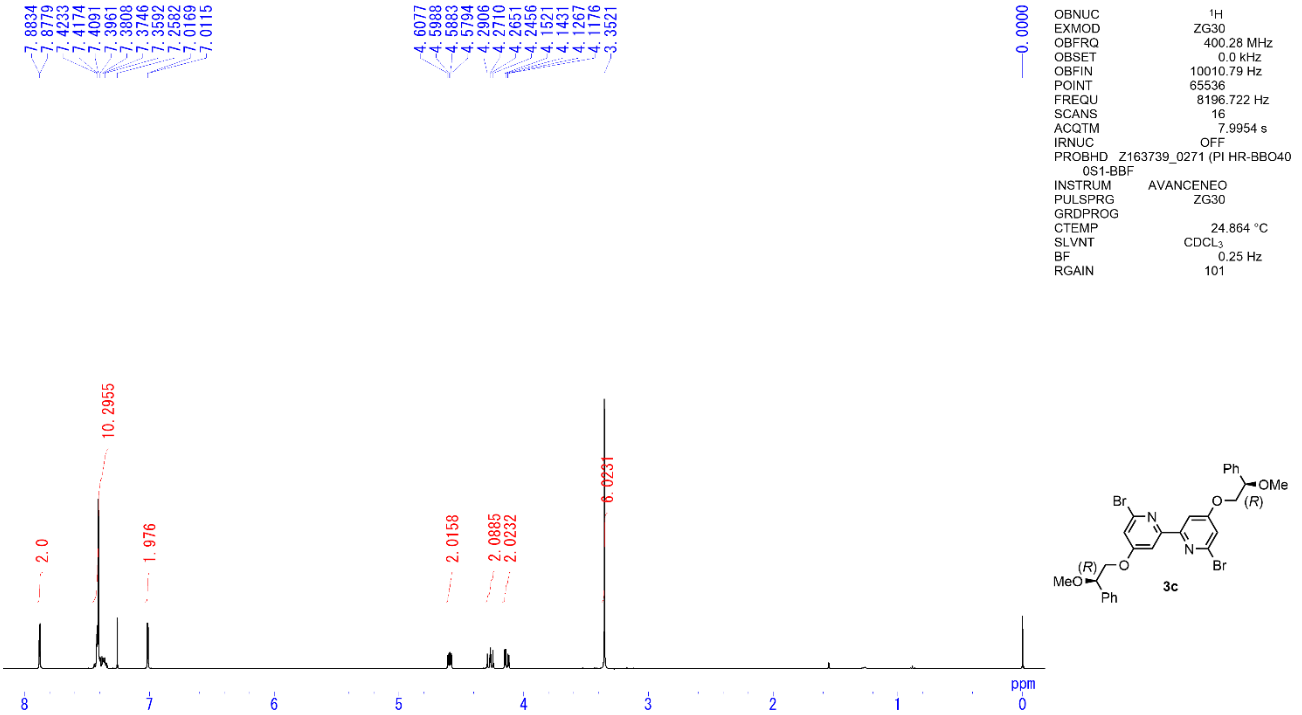


Figure S90. ¹H-NMR spectrum of **3c**.

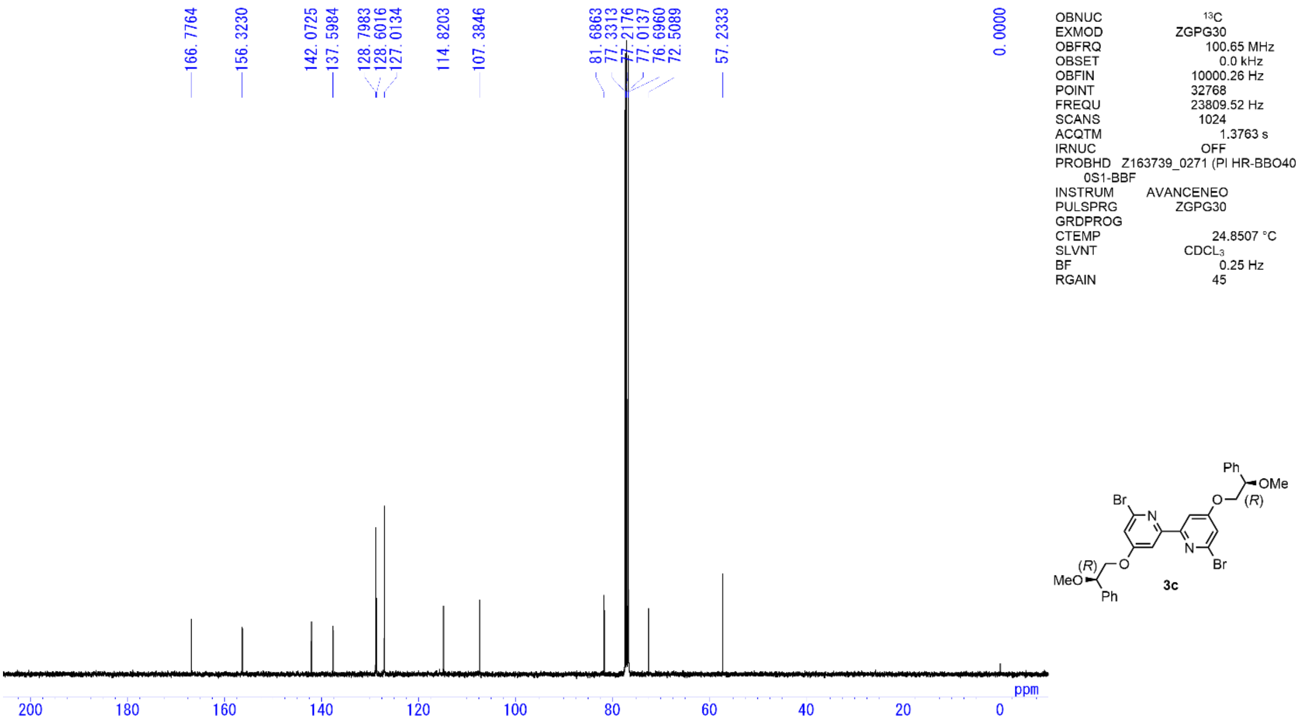


Figure S91. ¹³C-NMR spectrum of **3c**.

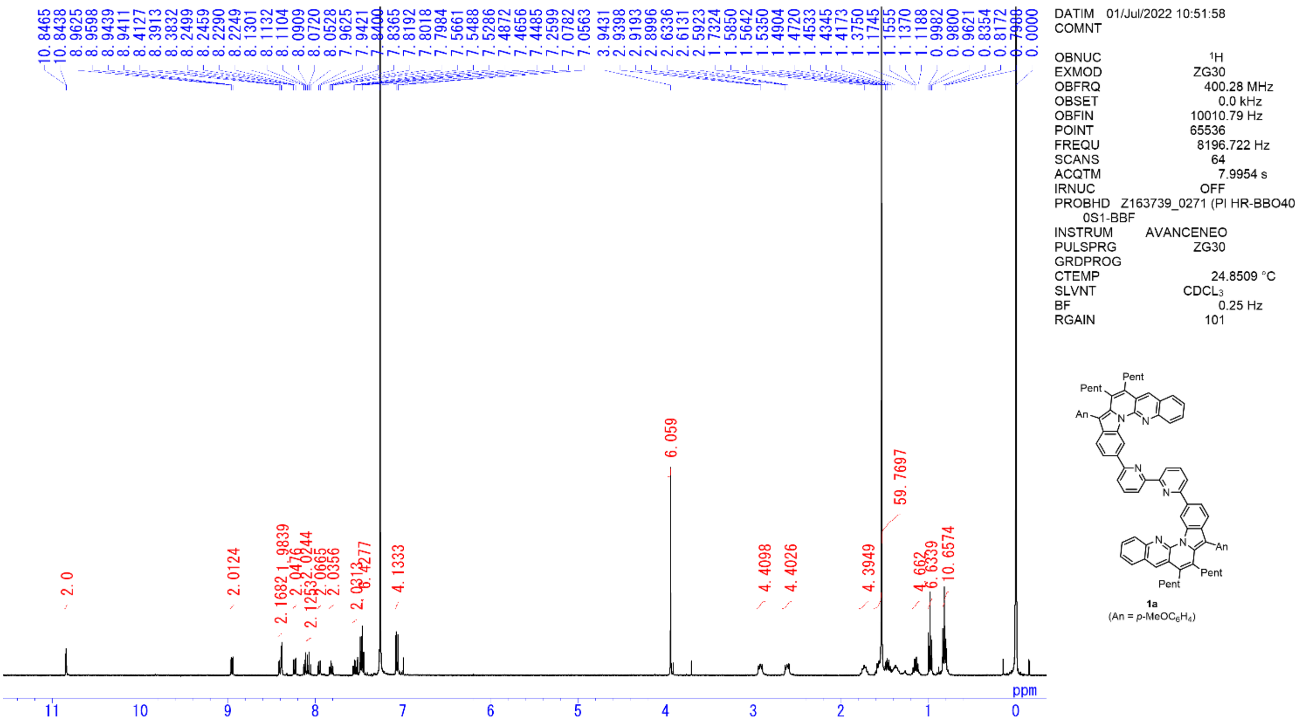

Figure S92. $^{1}$H-NMR spectrum of **1a**.

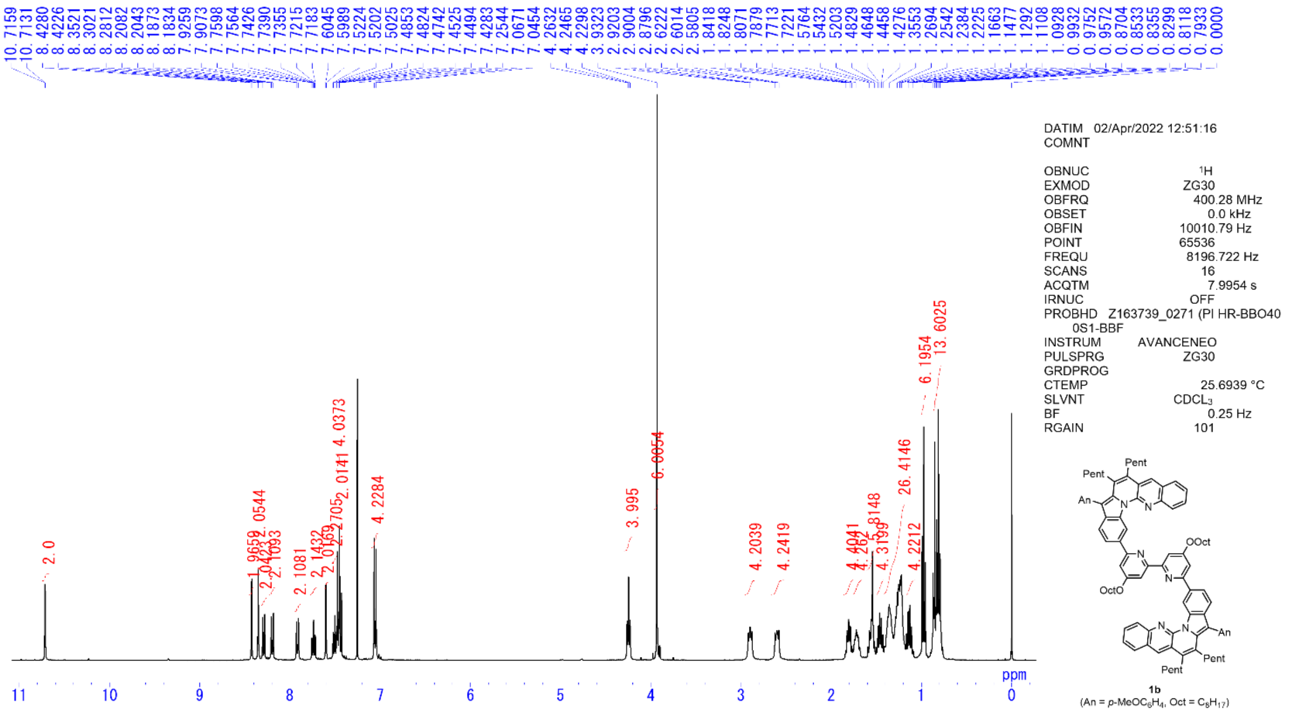


Figure S93. $^{1}$H-NMR spectrum of **1b**.

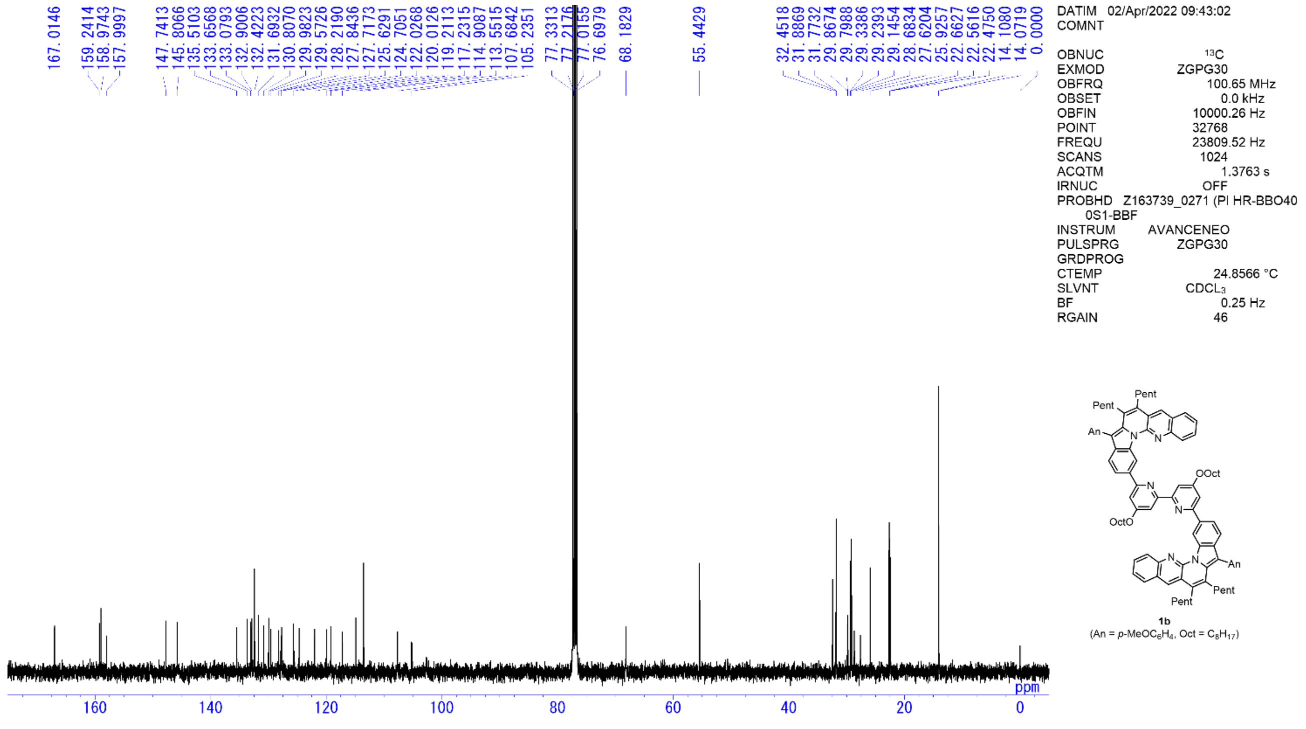


Figure S94. $^{13}$C-NMR spectrum of **1b**.

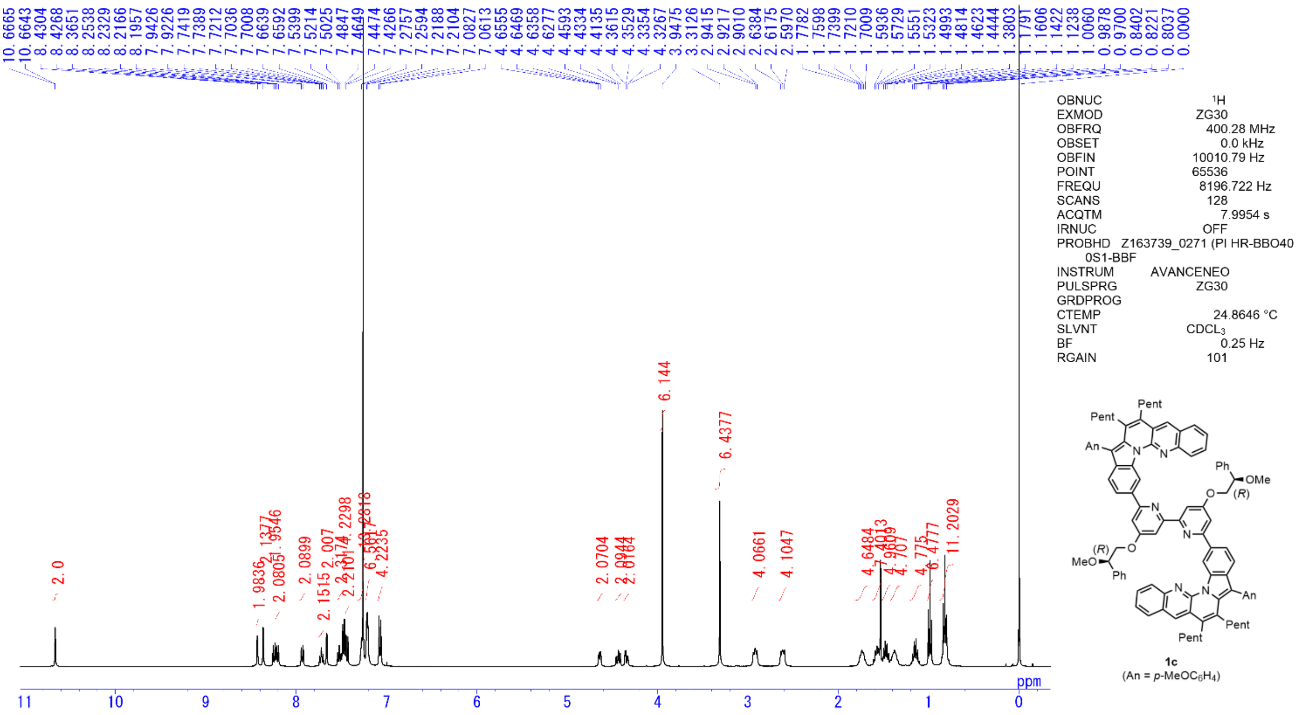


Figure S95. $^{1}$H-NMR spectrum of **1c**.

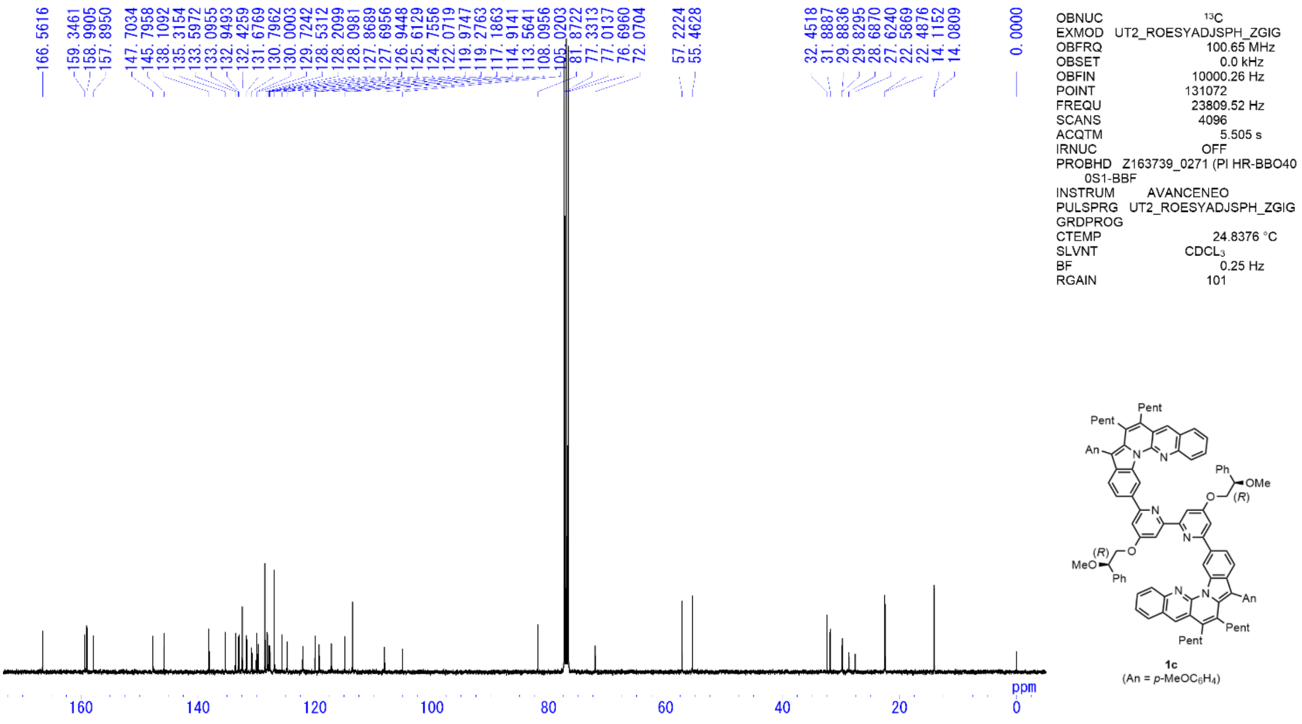


Figure S96. $^{13}$C-NMR spectrum of **1c**.

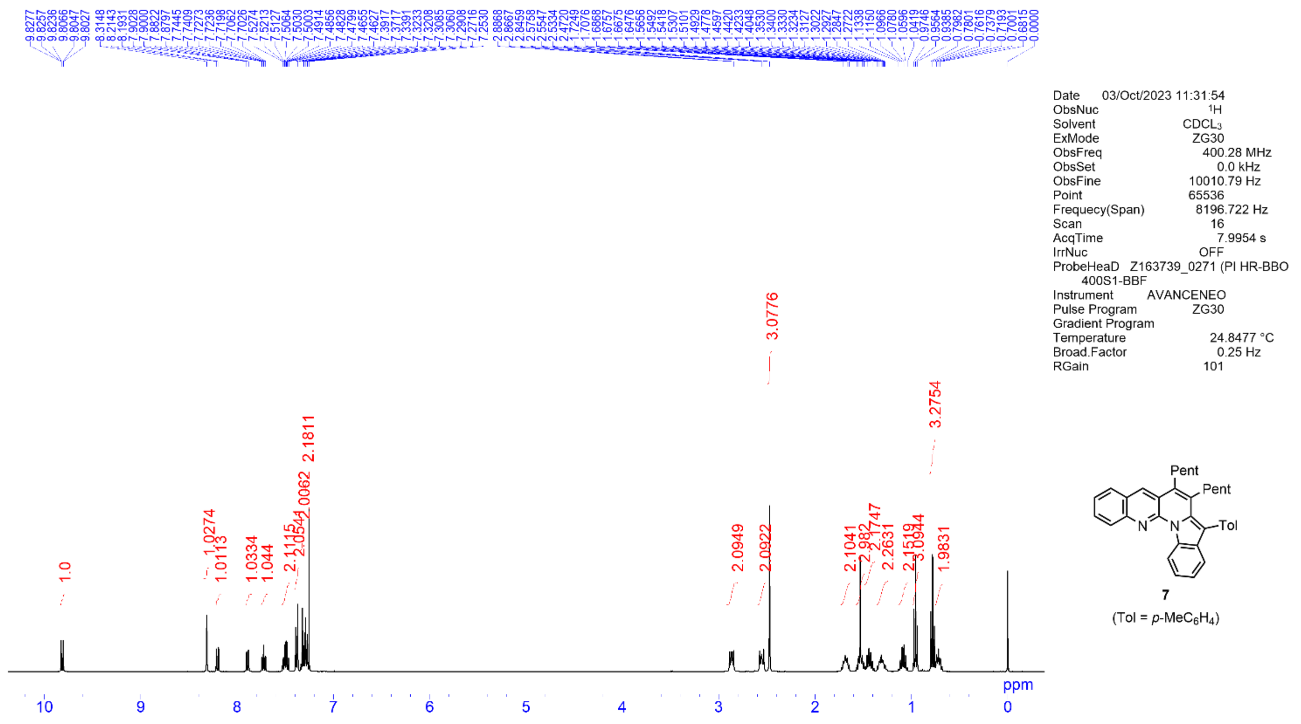


Figure S97. $^{1}$H-NMR spectrum of **7**

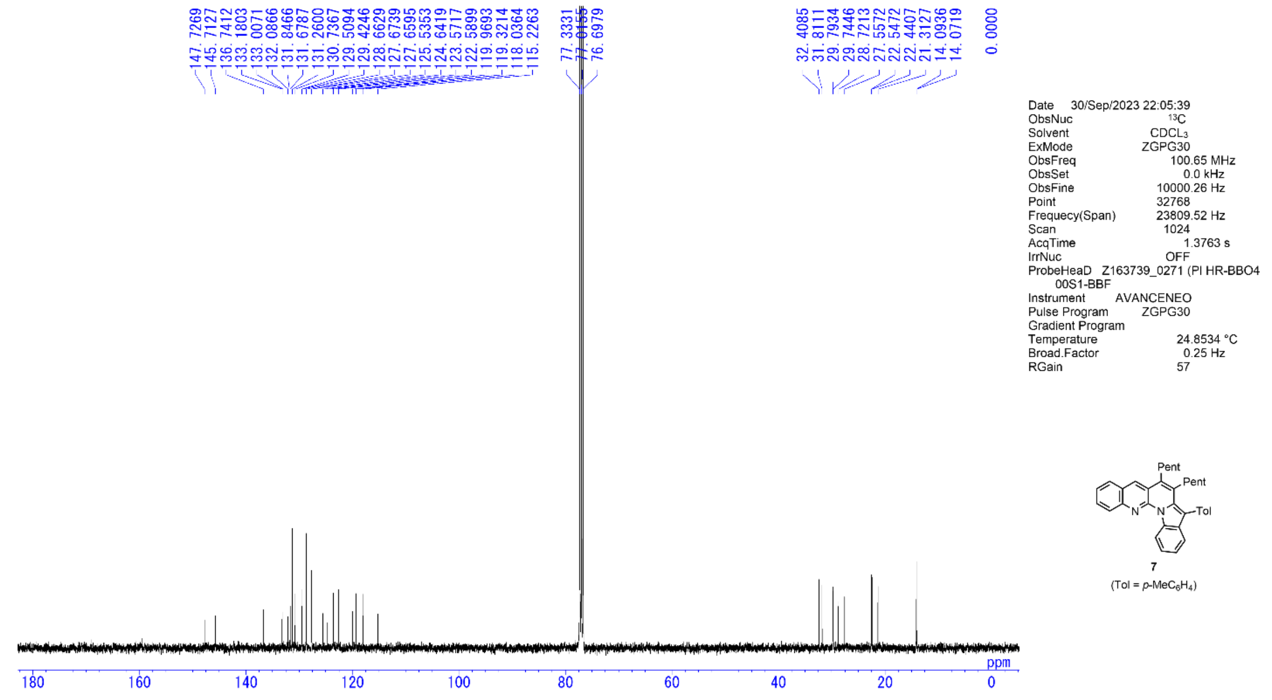


Figure S98. $^{13}$C-NMR spectrum of **7**

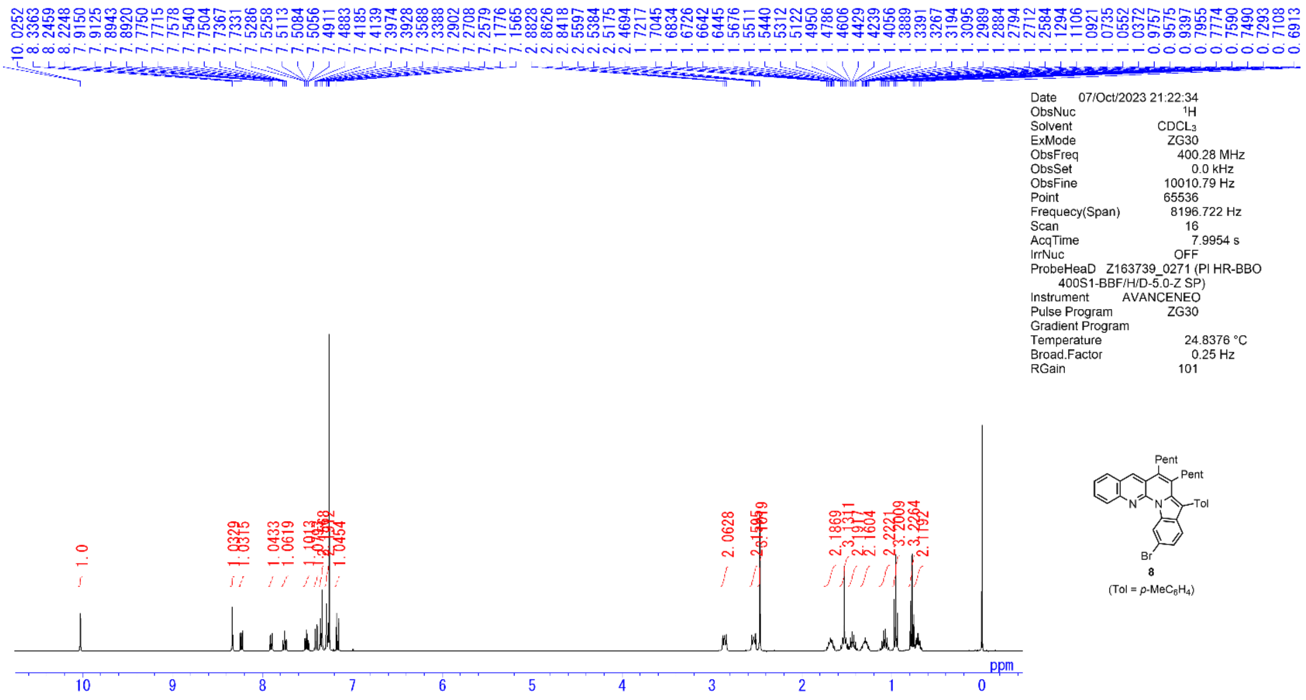


Figure S99. $^{1}$H-NMR spectrum of **8**.

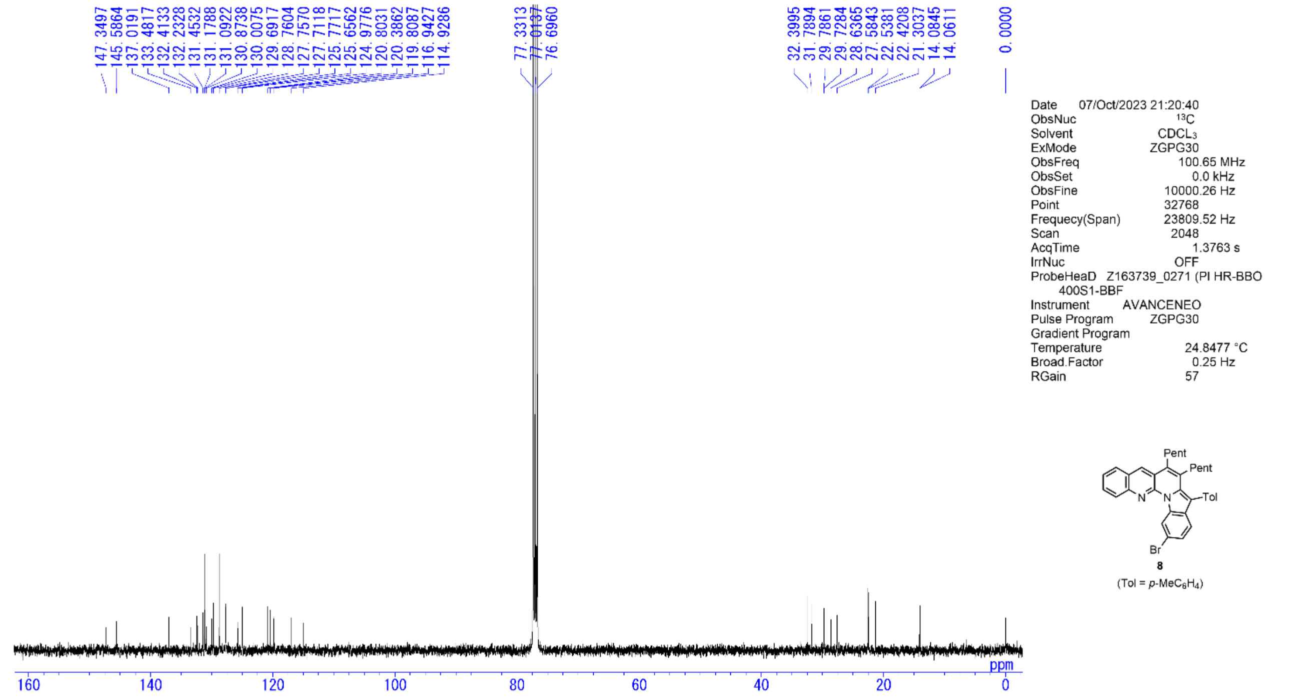


Figure S100. $^{13}$C-NMR spectrum of **8**

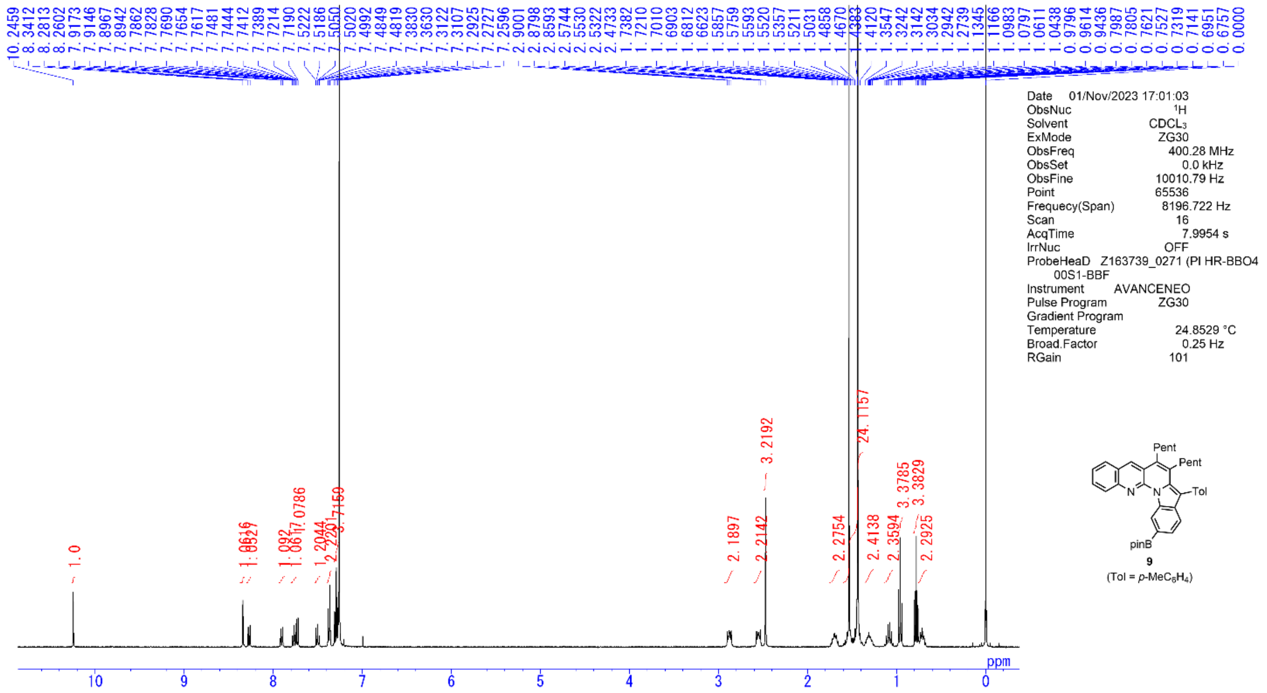


Figure S101. $^{1}$H-NMR spectrum of **9**.

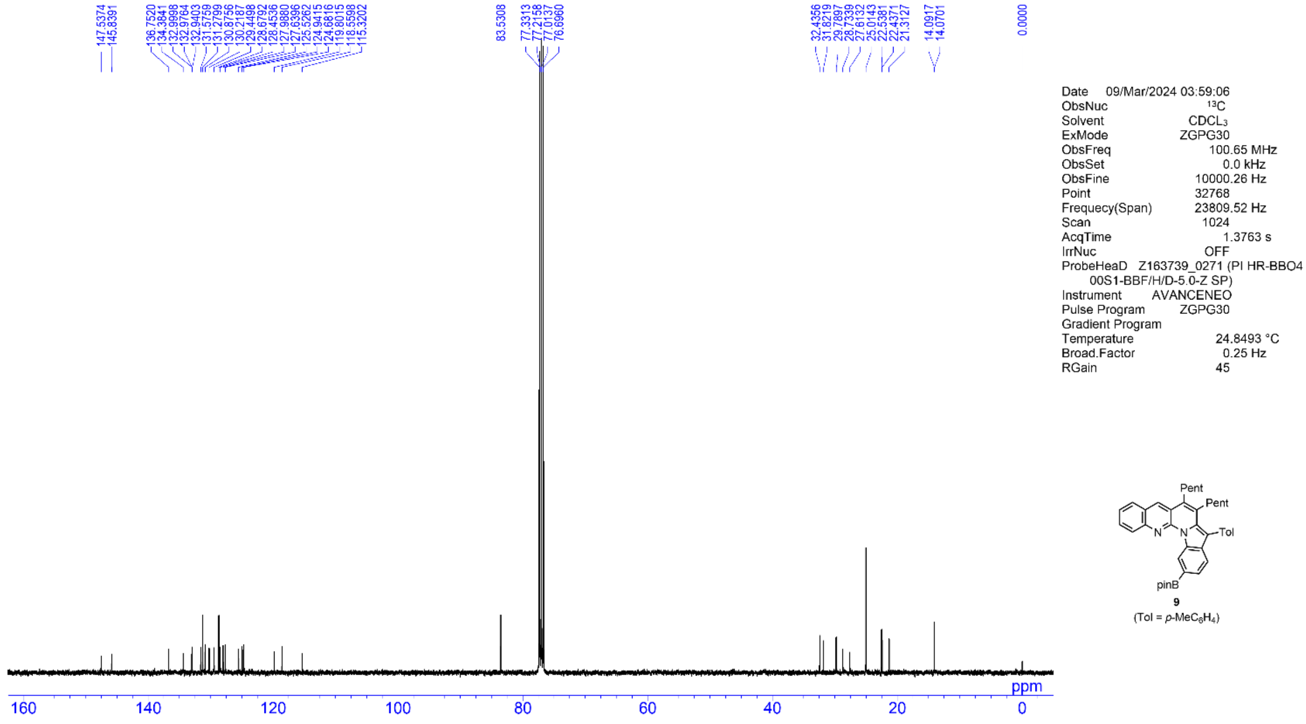


Figure S102. $^{13}$C-NMR spectrum of **9**.

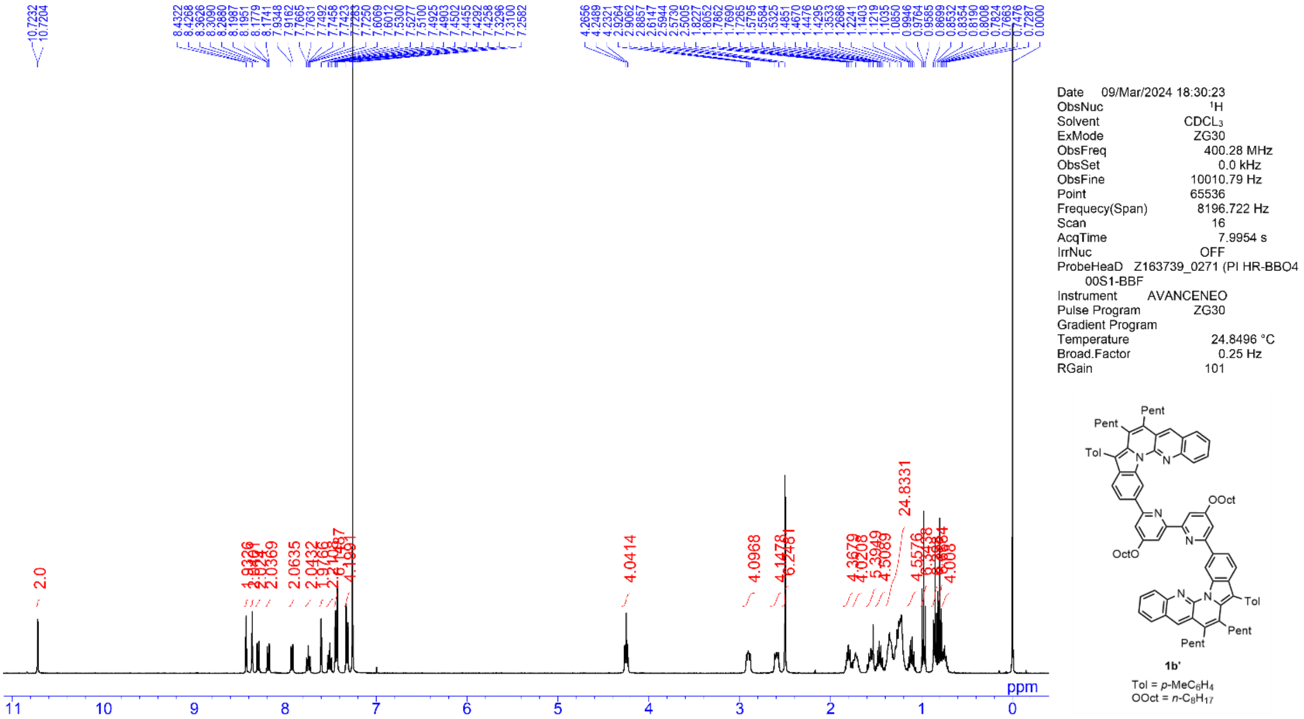


Figure S103. ¹H-NMR spectrum of **1b’**.

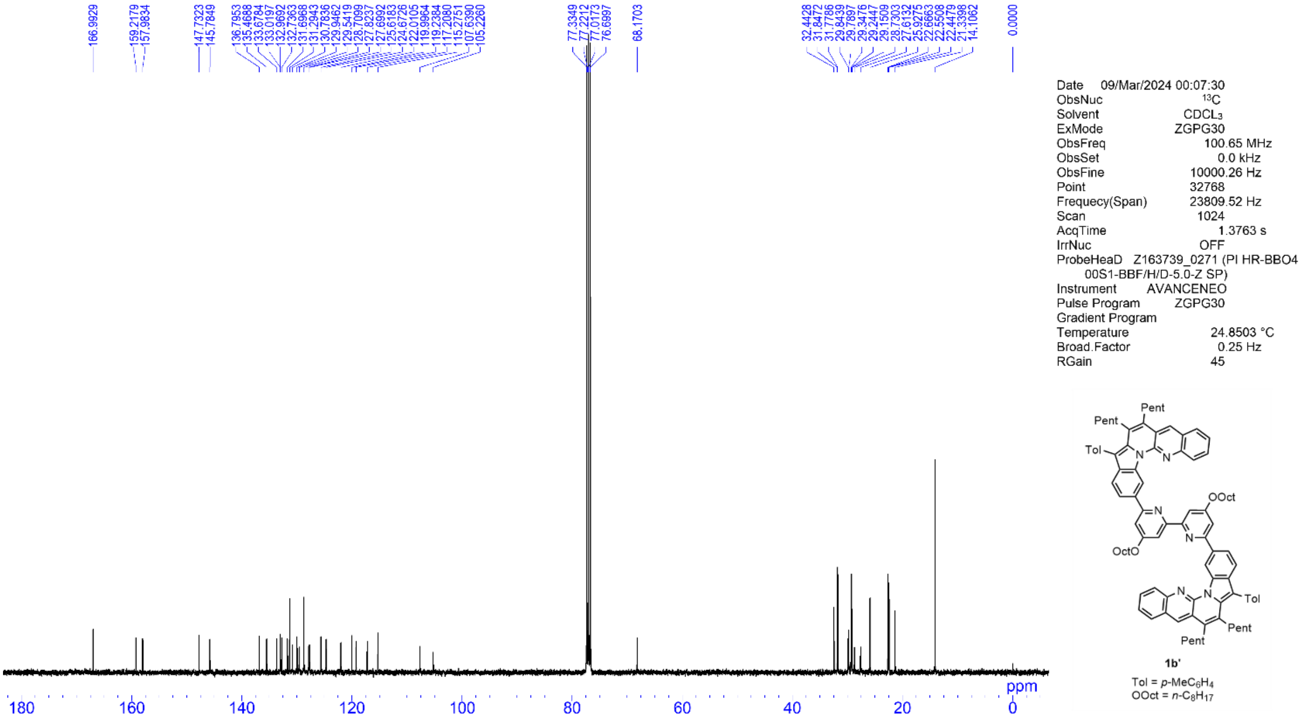


Figure S104. ¹³C-NMR spectrum of **1b’**.

## 15. References


[S1] Tateno, K.; Ono, K.; Kawai, H. Fluorescent Short-Stranded Helical Foldamers Based on L-shaped Dibenzopyrrolo[1,2-*a*][1,8]naphthyridine. *Chem. Eur. J.* **2019**, *25*,15765-15771.

[S2] Tateno, K.; Ogawa R.; Sakamoto, R.; Tsuchiya, M.; Otani, T.; Saito, T. Acid-Responsive Fluorescent Compounds Based on Nitro-Group-Substituted L-Shaped Pentacycles, Pyrrolo[1,2-*a*][1,8]naphthylidines. *Org. Lett.* **2014**, *16*, 3212–3215.

[S3] Barroso, S.; Joksch, M.; Puylaert, P.; Tin, S.; J. Bell, S.; Donnellan, L.; Duguid, S.; Muir, C.; Zhao, P.; Farina, V.; Tran, D. N.; Vries, J. G. Improvement in the Palladium-Catalyzed Miyaura Borylation Reaction by Optimization of the Base: Scope and Mechanistic Study. *J. Org. Chem.* **2021**, *86*, 103−109.

[S4] Sheldrick, G. M. A short history of SHELX. *Acta Cryst.* **2008**, *A64*, 112–122.

[S5] Sheldrick,G. M. *Acta Cryst.* **2015**, *C71*, 3–8.

[S6] Dolomanov, O. V; Bourhis, L. J; Gildea, R. J; Howard, J. A. K; Puschmann, H. *J. Appl. Cryst.* **2009**, *42*, 339–341.

[S7] Perrin,C. L; Dwyer, T. J. Application of two-dimensional NMR to kinetics of chemical exchange. *Chem. Rev.* **1990**, *90*, 935-967.

[S8] Evans, R; Deng, Z; Rogerson, A. K; McLachlan, A. S; Richards, J. J; Nilsson, M; Morris, G. A. Quantitative Interpretation of Diffusion-Ordered NMR Spectra: Can We Rationalize Small Molecule Diffusion Coefficients? *Angew. Chem. Int. Ed*. **2013**, *52*, 3199 –3202.

[S9] Neese, F. The ORCA program system. *WIREs Comput Mol Sci.* **2012**, *2*, 73–78.

[S10] Wennmohs, F; Becker, U; Riplimger, C. The ORCA quantum chemistry program package. *J. Chem. Phys*. **2020**, *152*, 224108.

[S11] Neese, F. Software update: The ORCA program system-Version 5.0. *WIREs Comput Mol Sci*. **2022**, *12*, e1606.

[S12] Grimme, S; Ehrlich, S; Goerigk, L. Effect of the Damping Function in Dispersion CorrectedDensity Functional Theory. *J Comput Chem*. **2011**, *32*, 1456–1465.

[S13] Grimme, S; Antony, J; Ehrlich, S; Krieg, H. A consistent and accurate ab initio parametrization of density functional dispersion correction (DFT-D) for the 94 elements H-Pu. *J. Chem. Phys*. **2010**, *132*, 154104.

[S14] Weigend, F; Ahlrichs, R. Balanced basis sets of split valence, triple zeta valence and quadruple zeta valence quality for H to Rn: Design and assessment of accuracy. *Phys. Chem. Chem. Phys.* **2005**, *7*, 3297-3305.

[S15] Weigend, F. Accurate Coulomb-fitting basis sets for H to Rn. *Phys. Chem. Chem. Phys.* **2006**, *8*, 1057-1065.
